# Where ppm Quantities of Silsesquioxanes Make a Difference—Silanes and Cage Siloxanes as TiO_2_ Dispersants and Stabilizers for Pigmented Epoxy Resins

**DOI:** 10.3390/ma15020494

**Published:** 2022-01-10

**Authors:** Dariusz Brząkalski, Robert E. Przekop, Miłosz Frydrych, Daria Pakuła, Marta Dobrosielska, Bogna Sztorch, Bogdan Marciniec

**Affiliations:** 1 Faculty of Chemistry, Adam Mickiewicz University in Poznań, 8 Uniwersytetu Poznańskiego, 61-614 Poznań, Poland; d.brzakalski@gmail.com (D.B.); frydrych@amu.edu.pl (M.F.); darpak@amu.edu.pl (D.P.); 2 Centre for Advanced Technologies, Adam Mickiewicz University in Poznań, 10 Uniwersytetu Poznańskiego, 61-614 Poznań, Poland; bogna.sztorch@amu.edu.pl; 3 Faculty of Materials Science and Engineering, Warsaw University of Technology, 141 Wołoska, 02-507 Warsaw, Poland; Marta.Dobrosielska@pw.edu.pl

**Keywords:** silsesquioxane, spherosilicate, cage siloxane, silane, POSS, composite, titanium white, coating, surface treatment, coupling agent

## Abstract

In this work, silsesquioxane and spherosilicate compounds were assessed as novel organosilicon coupling agents for surface modification of TiO_2_ in a green process, and compared with their conventional silane counterparts. The surface-treated TiO_2_ particles were then applied in preparation of epoxy (EP) composites and the aspects of pigment dispersion, suspension stability, hiding power, as well as the composite mechanical and thermal properties were discussed. The studied compounds loading was between 0.005–0.015% (50–150 ppm) in respect to the bulk composite mass and resulted in increase of suspension stability and hiding power by over an order of magnitude. It was found that these compounds may be an effective alternative for silane coupling agents, yet due to their low cost and simplicity of production and manipulation, silanes and siloxanes are still the most straight-forward options available. Nonetheless, the obtained findings might encourage tuning of silsesquioxane compounds structure and probably process itself if implementation of these novel organosilicon compounds as surface treatment agents is sought for special applications, e.g., high performance coating systems.

## 1. Introduction

In polymers industry, additives are applied to fulfil one or more of multiple tasks at once: to cut costs (extending fillers, mostly), to improve mechanical properties (plasticizers, reinforcing fillers, e.g., fibres, nanoparticles), to reduce flammability (fire retardants), to improve the processability for the given processing technology or the final surface properties (lubricants, defoamers), or to add colour to the material (pigments). For inorganic additives, their price is one of the main decisive factors behind considering a given additive both an extending filler and a special purpose additive (functional filler), or just the latter [1,2]. The more expensive additives need to be applied in reasonable quantities in order to keep the production of the desired material economically viable. In case of pigments, there are more and less economical options available, differing also in terms of pigmenting efficiency and environmental friendliness. Regarding white pigments, the most commonly used white pigment is titanium white (TiO_2_), characterized by high pigmenting efficiency thanks to its nanostructure and quite good dispersibility, and comprising 69% of the worldwide market of inorganic pigments in year 2000, and together with carbon black and iron oxides, for over 90% of the world market in 2009 [3,4]. At the same time, titanium white is relatively expensive (around 2500 EUR as of 2020 [5]). Although being an effective pigment, it naturally agglomerates in polymer matrix, which results in need for using it at higher loadings, especially in thin foils, to achieve the desired opacity/pigmentation. Therefore, achieving good dispersion of TiO_2_ within polymer translates to reduction of the amount of the pigment used, while obtaining the same pigmentation level and thus reducing the production price for the given plastic material, as well as saving the natural resources used. Titanium white is not considered a high performance pigment due to its cost and agglomeration tendency, therefore treatment procedures are applied in industry and being studied in R&D [3,4].

Epoxy resins are available on the market in different colours for two main reasons. The first one is obviously the aesthetic value, as they are often used for decoration/artistic purposes. Secondly, epoxy resins are commonly slightly yellow in colour, and even more often are the curing components (hardeners) thereof, especially the most common amine type ones. Upon storage, this yellow colour becomes more intense. Therefore, colouration/pigmentation of the epoxy allows to hide this discolouration and make the resin colour more reproducible from batch to batch during production. In case of storing the resin, it is important for the pigment to form a stable mixture with the epoxy base and not to separate from it upon time. Therefore, white-pigmented epoxy resins of satisfactory shelf-life are of high demand and increasing this shelf-life is sought [4,6]. Also, improved dispersion translates into increased hiding power, understood as the coating ability of optically blocking the surface it covers [7].

Organosilicon compounds are nowadays the most common agents for surface treatment of inorganic materials, the older and discontinued agents being chromium and titanium-based [8,9]. For over 20 years now, silsesquioxane derivatives have been studied as a novel class of organosilicon compounds suitable as polymer processing additives. In comparison to traditional silane coupling agents, their polarity, melting temperatures and vapour pressures are much lower, allowing them to be directly processed with a polymer, which is strongly limited for organofunctional silanes. Also, for a couple of years now, silsesquioxanes have been studied as a new class of silane coupling agents for treatment of inorganic fillers and other particles/nanoparticles. In terms of serving as direct polymer additives for epoxy resins, silsesquioxane compounds were introduced both in a reactive and non-reactive (physical blending) manner. For reactive additives, iOc_7_SSQ-(CH_2_)_3_NH_2_ [10], *i*Bu_7_SSQ-(CH_2_)_3_NH_2_ [11], 3-glycidoxypropylhepta(isobutyl)octasilsesquioxane [12], 3-glycidoxypropylhepta(isooctyl)octasilsesquioxane and 3-glycidoxypropylheptaphenyloctasilsesquioxane [13] epoxycyclohexylhepta(isobutyl)octasilsesquioxane [14], (3-glycidoxypropyl)silsesquioxane cage mix [14,15], tris(glycidyldimethylsiloxy)hepta(isobutyl)silsesquioxane [16,17], octakis(aminophenyl)octasilsesquioxane [18], octakis(3-aminopropyl)octasilsesquioxane [19] mixed substituent hexyl/4-glycidylbutyloctaspherosilicates [13], and hybrids of DGEBA with SSQ molecules attached as a side group [13]. For non-reactive additives, Ph_7_SSQ-3OH (phenyltrisilanol) and *i*Bu_7_SSQ-3OH (isobutyltrisilanol) [20], 9,10-dihydro-9-oxa-10-phosphaphenanthrene-10-oxide (DOPO)-modified silsesquioxanes [21], methyl- and methyl-vinyl polysilsesqioxanes [22] were used. Additionally, in recent years, green chemical approach towards preparation of hybrid materials is sought and the examples of the first silsesquioxane-containing materials prepared in aqueous conditions were given [23]. Silsesquioxanes, as well as polysilsesquioxanes, have been studied as additives moderating dielectric properties of nanocomposites and nanocomposite films [24].

For improved dispersion and interaction with the matrix polymer, TiO_2_ has been treated with Ph_7_SSQ-3OH [25,26] and *i*Bu_7_SSQ-3OH [27,28], for processing of polyolefin nanocomposites; *i*Bu_7_SSQ-(CH_2_)_3_NH_2_ (for preparation of hybrid graphene oxide-TiO_2_ material and cyanate ester composite containing thereof [29]), as to verify these compounds as novel coupling agents for surface treatment of inorganic nanoparticles.

In this work, an approach towards application of well-defined organosilicon compounds, that is, silsesquioxane and spherosilicate derivatives, as dispersants and stabilizers of TiO_2_ nano- and microparticles is presented. More importantly, they were used at low loadings for the studied solution to be economically viable, as well as technologically feasible during standard procedures of pigment manufacturing or pigmented epoxy preparation, without no additional chemical processing steps or organic solvents involved during surface treatment. The organosilicon compounds are compared to much cheaper and readily available, conventional silane coupling agents (trialkoxysilanes) to assess any possible advantages of the former. 

## 2. Materials and Methods

### 2.1. Materials and Instrumentation

The chemicals were purchased from the following sources: Tetraethoxysilane (TEOS), tetrachlorosilane, chlorodimethylsilane, tetramethylammonium hydroxide (TMAH) 25% methanol solution from ABCR, *iso*butyltrimethoxysilane, triethylamine, allyl-glycidyl ether, vinyltrimethoxysilane, chloroform-d and Karstedt’s catalyst xylene solution from Aldrich, P_2_O_5_, tetrahydrofuran (THF), methanol, hydrochloric acid, toluene, acetonitrile, and acetone from Avantor (Poland). Toluene was degassed and dried by distilling it from P_2_O_5_ under argon atmosphere. TiO_2_ was obtained from Grupa Azoty Z.Ch. “Police” S.A. (Poland), as a 50% *w*/*w* water slurry with no additives. Epidian 5 was obtained from Ciech Sarzyna (Poland) and according to the manufacturer’s data, was characterized by density of 1.17 g/cm^3^ and viscosity of 20,000–30,000 mPa·s at 25 °C, and epoxide number of 0.48–0.51 mol/100 g. Curing agent, triethylenetetramine (branded as Z-1 hardener), was also purchased from Ciech.

Octahydrospherosilicate was prepared according to a literature procedure [30]. Chlorohepta(isobutyl)octasilsesquioxane was prepared according to literature procedures [31,32].

^1^H, ^13^C, and ^29^Si Nuclear Magnetic Resonance (NMR) spectra were recorded at 25 °C on a Bruker Ascend 400 and Ultra Shield 300 spectrometers using CDCl_3_ as a solvent. Chemical shifts are reported in ppm with reference to the residual solvent (CHCl_3_) peaks for ^1^H and ^13^C.

Fourier Transform-Infrared (FT-IR) spectra were recorded on a Nicolet iS 50 Fourier transform spectrophotometer (Thermo Fisher Scientific) equipped with a diamond ATR unit with a resolution of 0.09 cm^−1^.

Differential scanning calorimetry (DSC) was performed using a NETZSCH 204 F1 Phoenix calorimeter. Samples of 6 ± 0.2 mg were placed in an aluminium crucible with a punctured lid. The measurements were performed under nitrogen in the temperature range of −30–230°C and at a 5 °C/min heating rate. T_gu_ was measured from the first heating cycle, T_g_ was measured from the second heating cycle.

MALDI-TOF mass spectra were recorded on a UltrafleXtreme mass spectrometer (Bruker Daltonics), equipped with a SmartBeam II laser (355 nm) in the 500–4000 m/z range. 2,5-Dihydroxybenzoic acid (DHB, Bruker Daltonics, Bremen, Germany) served as matrix. Mass spectra were measured in reflection mode. The data were analysed using the software provided with the Ultraflex instrument—FlexAnalysis (version 3.4).

SEM/EDS analyses were recorded on a Quanta FEG 250 (FEI) instrument; SEM at 5 kV and EDS at 30 kV, respectively. The samples were frozen in liquid nitrogen and fractured with pliers to reveal satisfactory surface for analysis.

Rheological measurements were performed on Anton Paar MCR302 dynamic mechanical thermal (DMTA) rheometer, working in plate-plate configuration, using 25 mm plates.

Contact angle analyses were performed by the sessile drop technique at room temperature and atmospheric pressure, with a Krüss DSA100 goniometer. Three independent measurements were performed for each sample, each with a 5 µL water drop, and the obtained results were averaged to reduce the impact of surface nonuniformity.

For tensile and flexural strength tests, a Universal testing machine Instron 5969 was used, in accordance to the norm EN ISO 527-2:1996. The speed of traverse was set to 2 mm/min for both tensile strength and flexural strength tests.

The measurements of hiding power were performed by placing the samples of prepared resin systems in the optical path between the light source (an LED)and a UV-NIR spectrophotometer, AvaSpec-Mini2048CL (Avantes, Louisville, CO, USA). The amount of light transmitted was through the sample was measured and on this basis, the relative hiding strength was determined, 1Ti(s) sample being used as a reference of the lowest value.

**Figure 1:**
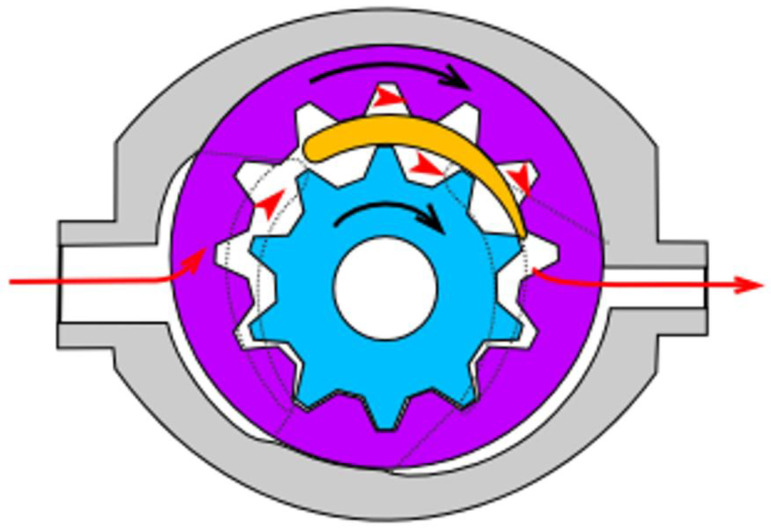
Internal gear pump (crescent type) design scheme. Red arrows present the pathway of the pumped medium, the black ones—the rotation direction of the gears. Source: https://commons.wikimedia.org/w/index.php?curid=38795, Creative Commons license CC BY-SA 3.0. Accessed on 2 September 2021.

**Figure 2:**
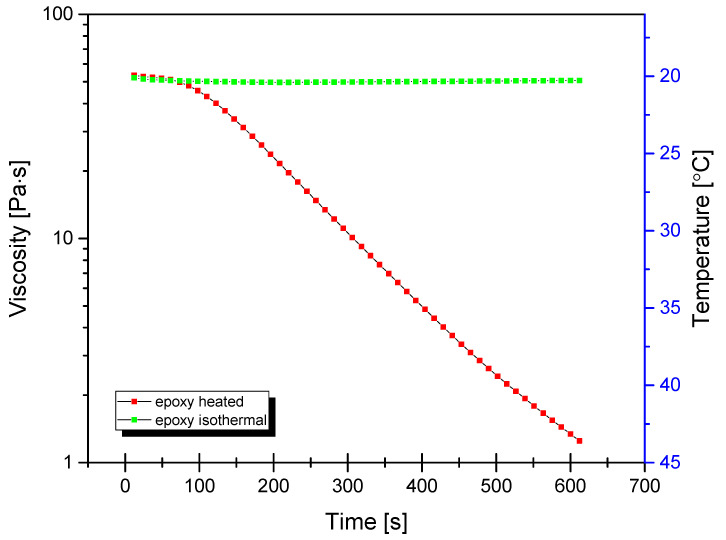
Dynamic viscosity curves of neat epoxy under isothermal and non-isothermal conditions under constant shear rate of 100/s.

For mixing of base epoxy resin with TiO_2_, a self-made mixing device was used (see Supplementary Information, Figure S1). The machine was based on a high-torque Internal gear (Crescent type, Figure 1) rotary positive displacement pump (Yildiz Pompa YKF 1, Turkey) powered by a 0.75 kW electrical engine (Marelli Motori D6C 90 S6 B3, Italy). The setup was characterized by 900 rpm of maximum operating speed. The pump was connected to a reservoir in a closed circuit setup, resulting in constant circulation of the resin under high shear stress, inflicting mechanical breakdown of TiO_2_ particles and improved dispersion of TiO_2_ within the epoxy resin. During the mixing cycle, the resin would heat up to ~45 °C due to internal friction, which resulted in a significant viscosity drop (as simulated by DMTA analysis under constant shear and temperature gradient conditions, presented in Figure 2), which additionally helped with nanoparticle dispersion.

### 2.2. Procedure for Hydrosilylation of Octaspherosilicates

All hydrosilylation syntheses were conducted under argon atmosphere in round-bottom flasks equipped with condensers, gas bubblers, and magnetic stirrers, the general approach being reported earlier [33]. In a typical procedure, a 500 mL three-neck, round-bottom flask was charged with 25 g of octahydrospherosilicate, 250 mL of dry toluene and a mixture of olefins (allyl-glycidyl ether and vinyltrimethoxysilane, 6:2 molar ratio for SS-6GP-2TMOS, 5:3 ratio for SS-5GP-3TMOS). A thermometer and condenser equipped with an argon inlet and oil bubbler were attached, the flask placed in a heating mantle and the system was purged with argon. The reaction mixture was set on 110 °C and before reaching boiling, Karstedt’s catalyst solution (10^−5^ eq Pt/mol SiH) was added, which resulted in quick increase of temperature and the system starting to reflux. The reaction mixture was kept at the boiling temperature and samples were taken for FT-IR control until full Si-H group consumption was observed. Then, the solvent was evaporated under vacuum to dryness to obtain an analytically pure sample. The products appeared as low-viscosity oils.

### 2.3. Procedure for Preparation of iBu_7_SSQ-OEt

The synthesis was conducted under argon atmosphere in a round-bottom flask equipped with a condenser, a gas bubbler, and a magnetic stirrer. A 500 mL three-neck, round-bottom flask was charged with 15 g of chlorohepta(isobutyl)octasilsesquioxane. A thermometer and condenser equipped with an argon inlet and oil bubbler were attached and the system was purged with argon. After that, the flask was further charged with 250 mL of dry THF and 4.9 mL of NEt_3_ (2 eq in correspondence to Si-Cl group), the flask was placed in a heating mantle, and 50 mL of dry EtOH was slowly added. The mixture was heated to 50 °C and stirred for 3 h. Then, the reaction was transferred to a rotary evaporator, the solvent mixture removed almost to dryness, 250 mL of hexane was added to the remaining solid, and the suspension was sonicated for 30 min. The obtained mixture, comprised of the main product solution and triethylammonium hydrochloride suspension, was filtered through a sintered glass funnel to eliminate the ammonium hydrochloride solid, the flask being washed with additional 150 mL of hexane, and the obtained clear hexane solution was evaporated to dryness to obtain analytically pure sample. The product appeared as a white solid.

### 2.4. Procedure for Treatment of TiO_2_ with Organosilicon Compounds

In a typical procedure, a 2.5 L milling jar was charged with 750 g of 50% TiO_2_ water slurry, 100 mL of demineralized water (to reduce the mixture viscosity), 20 ceramic milling balls of 20 mm diameter, and either 1.875 g or 5.625 g (corresponding to either 0.5% or 1.5% *w*/*w* of dry TiO_2_) of a chosen organosilicon coupling agent (see Table 1 and Table 2 and Figure 2). The milling jar was closed and placed on the ball mill, and rotated at 30 rpm for 20 h. After that, the slurry was transferred to a container, dried at 105 °C to constant weight and milled again in a ball mill for 20 h. The obtained modified TiO_2_ was then sampled for water contact angle measurements and used for preparation of TiO_2_/EP composites. Tests for treatment of TiO_2_ with 3% and 5% loadings of organosilicon coupling agents were also performed, but the resulting materials were waxy and/or sticky and difficult to handle, which was a sign of the coupling agents being used in excess. Therefore, only the samples treated with 0.5% and 1.5% of coupling agents were used for further studies.

### 2.5. Procedure for Preparation of TiO_2_/EP Composites

In a typical procedure, the mixing pump was charged with around 1 kg of base epoxy resin and the stirring was engaged. After 5 min, TiO_2_ sample of a given modification grade (see Table 2) was added in an amount corresponding to either 1 wt% or 2 wt% of the final composition and stirred for further 15 min at 800 rpm. After that, the resin mixture was transferred to a container, chilled to room temperature, and sampled for pigment stability tests. Next, the resin mixture was mixed with 12 wt% of the curing agent (Z-1) using a mechanical stirrer, degassed for 5 min in a vacuum chamber, and cast into plates of 4 mm thickness. After 16 h, the plates were removed from the moulds and set for seven days for further curing (see Supplementary Information, Figure S2). After that time, the plates were cut into standardized test specimens for mechanical analysis (dumbbells and bars) with the aid of a CNC milling plotter. The milled specimens were additionally post-cured for 17 h at 70 °C to ensure the most uniform curing state for all tested samples. Such prepared samples were used for mechanical analyses. 

For the samples prepared with the mechanical stirrer (Table 2), the resin was mixed with TiO_2_ for the same amount of time with the aid of the mechanical stirrer instead of the dissolver, and the rest of the procedure remained unchanged. 

**Figure 3:**
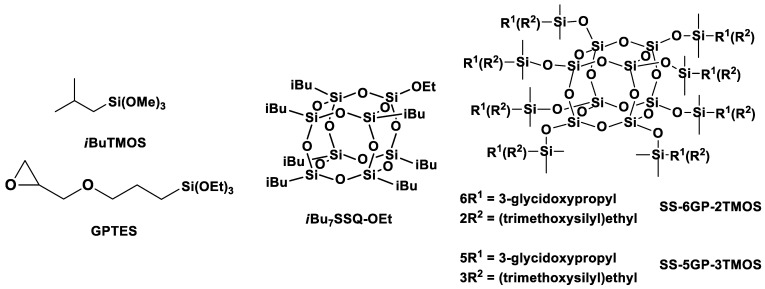
Structures of the silane and cage siloxane compounds studied in this work.

The composite sample codes are as follows:

x Ti y CODE (z)(1)

where x—% loading of TiO_2_ in the composite; y—% loading of a given silane coupling agent in correspondence to TiO_2_; CODE—the type of organosilicon coupling agent used (see Table 1, Figure 3); z—mixing method: ‘s’ for mechanical stirrer, ‘p’ for mixing pump

## 3. Results and Discussion

### 3.1. Characterization of the Obtained Products

**Figure 4:**
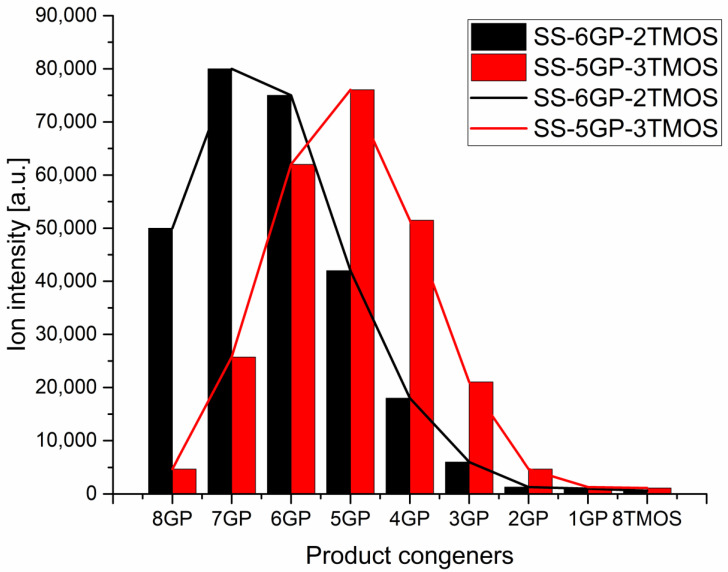
Distributions of congener products for SS-6GP-2TMOS and SS-5GP-3TMOS syntheses presented as MS signal intensity.

The products were characterized by ^1^H, ^13^C, ^29^Si NMR spectroscopy and MALDI-TOF mass spectrometry to verify the obtained molecular structures (see Supplementary Information). While iBu_7_SSQ-OEt was obtained as a single product, for SS-6GP-2TMOS and SS-5GP-3TMOS, it was observed on MALDI-TOF spectrograms that due to random introduction of the olefin substrates into the spherosilicate cage, a mixture of products was obtained in both cases and nine molecules were formed instead of the one that a given synthesis is designated with. Although the macroscopic stoichiometry of the systems was controlled by using the proper amounts of the olefin reagents, the intramolecular stoichiometry cannot be controlled and the synthesis affords a family of nine congeners, each containing a different internal ratio of organic substituents coming from the parent olefins, that is, vinyltrimethoxysilane and allyl-glycidyl ether. On the basis of the normalized signal intensity of the congener ions (in a form of sodium ion adducts) recorded by MS, normal distributions similar to Gaussian model were obtained, with the maximum visible near the intended system stoichiometry (Figure 4). Also, the peaks on the left side of the maximum are higher than those on the right for both systems, which can be explained on the basis of different ionization efficiency of glycidyl and alkoxysilyl groups, the glycidyl groups showing higher ionization efficiency. The effect of chemical structure on the ionization efficiency has been studied and discussed by other groups [34,35]. Therefore, this distribution should not be mistakenly considered as a direct depiction of the actual quantitative composition of the studied systems, and rather a qualitative one. It can be seen that for SS-6GP-2TMOS, the maximum is at the SS-7GP-1TMOS congener, which is likely due to the effect discussed above.

### 3.2. Surface Properties of the Obtained Modified TiO_2_ Pigments

Modified TiO_2_ pigments were analysed for their surface properties by measuring their water contact angle values (Table 3). Water contact angle is a non-direct measure of surface polarity of materials, as water is repelled by hydrophobic (non-polar) materials, thus resulting in formation of droplets with high contact angle on the surface of the measured samples. TiO_2_, as an inorganic nanomaterial, shows a strongly polar surface character, which is a reason for the particles thereof to agglomerate in polymer matrices, as they are usually characterized by substantially lower polarity. Introduction of organic (or organosilicon) groups onto the surface of TiO_2_ nanoparticles allows for controlling of their surface character, that is chemistry and polarity. It then translates into better filler-matrix interaction, without which a filler (in this case the pigment) tends to agglomerate [36]. 

**Figure 5:**
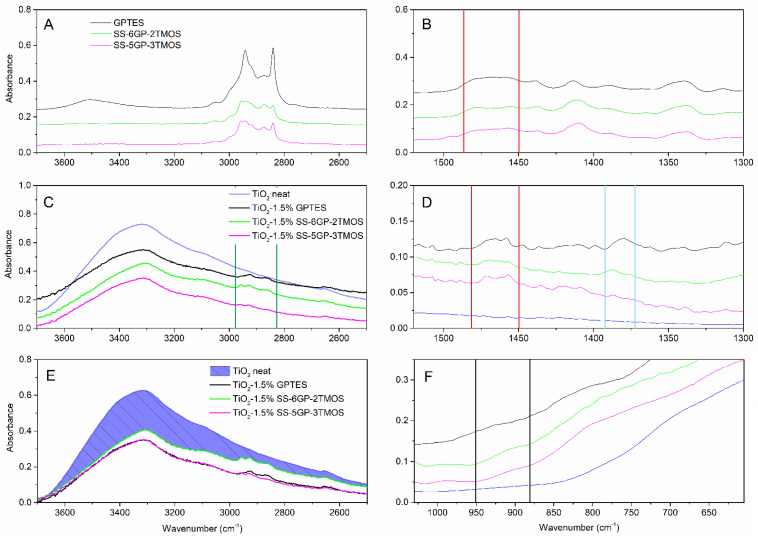
FT-IR of glycidyl-derived silane coupling agents (**A**,**B**), pristine TiO_2_ and TiO_2_ modified thereof (**C**–**F**). Red lines represent the region of oxirane ring vibrations, the blue lines—the diol vibrations, green ones—C-H vibrations, black ones—Ti-O-Si vibrations.

The samples treated with any of the glycidyl ether-derived compounds (GPTES, SS-6GP-2TMOS, SS-5GP-3TMOS) showed retainment of their hydrophilic character independently of the amount of the silane agent used, the water droplets being quickly absorbed by the material. It might be either due to polar character of glycidyl group itself, or its partial hydrolysis to even more hydrophilic diol group on the surface of TiO_2_. Kochkar et al. reported that oxirane moiety hydrolyzed to diol one partially when studying epoxidation of cyclohexene on a TiO_2_-SiO_2_-mixed catalyst [37]. This hypothesis is supported by FT-IR spectra of the glycidyl-derived compounds used and the TiO_2_ samples modified with those. The IR spectra allow to observe the effect of capping TiO_2_ hydroxyl groups, as the −OH stretching band is smaller for modified pigments than for the neat titanium white (Figure 5E, the reduction of absorption intensity marked with shaded area) as well as Ti-O-Si moiety vibrations being visible as a small bulge at ~910 cm^−1^ (Figure 5F), which is in agreement with the literature reports [38,39] Additionally, C-H vibrations are visible, coming from organosilicon additives grafted on the pigment particles surface (Figure 5A,C, green lines). However, while the absorption bands at 1450–1490 cm^−1^, characteristic for C-C oxirane ring vibrations are visible for both the organosilicon compounds and for the obtained pigments (Figure 5B,D, red lines), confirming the successful functionalization thereof, the FT-IR spectra of the latter also show additional absorption bands matching the vibration of the diol C-OH bonds (Figure 5D, blue lines, the oxirane ring opening presented on Figure 8). It is the most clear for the TiO_2_–1.5%GPTES sample, with an absorption band with the maximum at ~1380 cm^−1^, however the remaining modified TiO_2_ samples also show similar absorption at ~1385 cm^−1^. 

**Figure 6:**
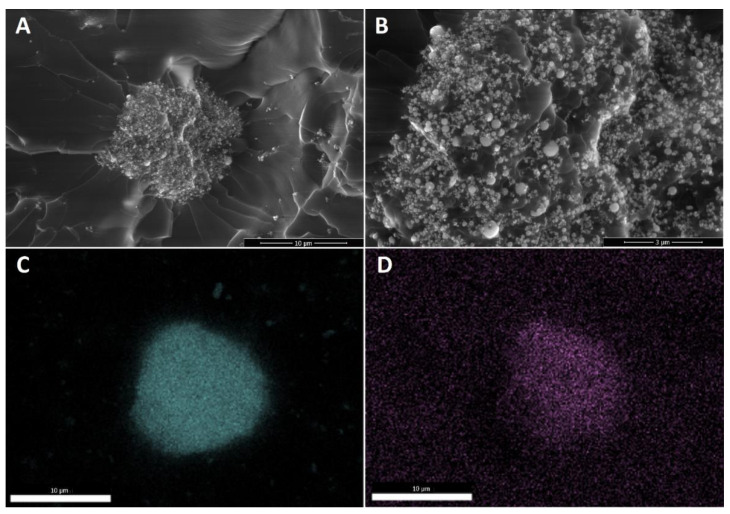
Microdroplets of condensed SS-6GP-2TMOS together with TiO_2_ particles visible for 1Ti15SS-6GP-2TMOS. (**A**)—10,000× magnification; (**B**)—30,000× magnification; (**C**)—titanium (Ti) EDS; (**D**)—silicon (Si) EDS.

**Figure 7:**
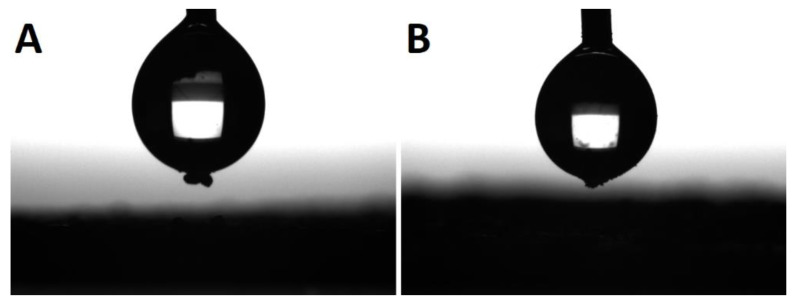
Water droplets during sessile drop analysis. Particles of modified TiO_2_ visible on the bottom of each droplet after an attempt at placing the droplet on the pigment surface. (**A**)—TiO_2_-1.5% *i*BuTMOS; (**B**)—TiO_2_-1.5% *i*Bu_7_SSQ-OEt.

The last effect considered is the gelation of the silane agent, thus reducing the coating effect of the silane agent. SS-6GP-2TMOS was observed on SEM images to form microdroplets accompanying TiO_2_ particles in the final composite (Figure 6A,B visualizing the microdroplets on TiO_2_, Figure 6D confirming the silicon-rich area on the TiO_2_ microaggregate). On the other hand, butylated agents (both *i*BuTMOS and *i*Bu_7_SSQ-OEt) showed strong hydrophobicizing action, as the obtained materials were superhydrophobic. The droplets formed were completely repulsed by TiO_2_, making it impossible to lay a droplet for the measurement, and when dropped from air, they would immediately roll off even at the angle of ~0° (Figure 7, particles of TiO_2_ visible on the bottom surface of the water droplets after attempts of placing the droplets on the samples’ surface). 

**Figure 8:**
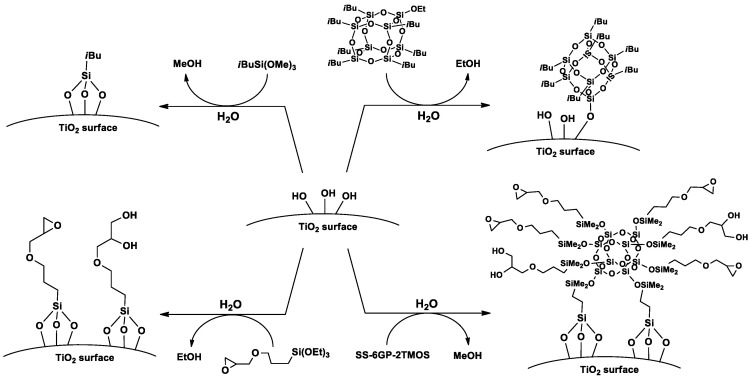
A proposed mechanism of TiO_2_ surface silanization with *i*Bu_7_SSQ-OEt, SS-6GP-2TMOS and silane coupling agents.

Based on the FT-IR spectra and water contact angle measurements, proposed mechanisms of TiO_2_ surface functionalization with the studied organosilicon coupling agents is presented on Figure 8.

### 3.3. Microscopic Analysis and the Effect of Processing Methodology on TiO_2_ Dispersion

**Figure 9:**
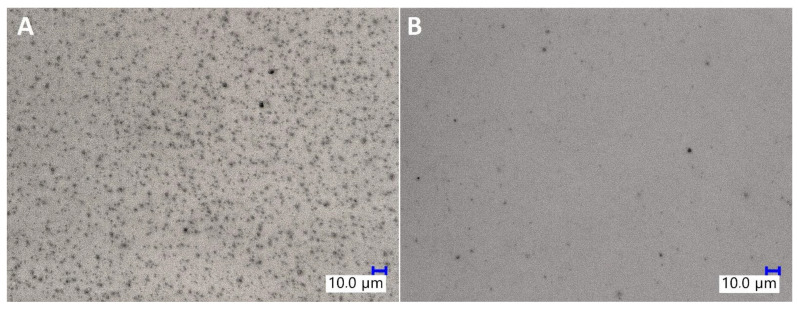
Digital optical microscopic images of neat TiO_2_/EP (**A**) and 1.5% *i*Bu_7_SSQ-OEt TiO_2_/EP (**B**).

SEM imaging was used to assess the dispersion of TiO_2_ particles in the epoxy matrix (see Supplementary Information Section 4: SEM images of the TiO_2_/EP composites). It was observed that the sample prepared with untreated TiO_2_ by mechanical stirring showed the largest agglomeration effect, as not only the particles of TiO_2_ formed multi-micron aggregates of size up to 50 μm, but also these aggregates were surrounded by barely no well-dispersed microparticles. On the other hand, both high-torque pump stirring and application of organosilicon coupling agents improved the dispersion of the pigment. It is visible on the SEM images of samples containing pristine TiO_2_ that after pump mixing, the primary aggregates observed are of size up to approx. 20 μm, and that they are accompanied by a fraction of particles of <2 μm. Also, for all the sample series, the increase in TiO_2_ loading from 1% to 2% resulted in a higher number of aggregates visible on the SEM images. At 0.5%, *i*BuTMOS was a highly effective dispersing agent both under mechanical stirring and pump stirring conditions, the latter resulting in almost complete elimination of TiO_2_ aggregates above 2 μm, when the pigment was at the 1% loading. At 1.5%, *i*BuTMOS and 1% TiO_2_ loading, all the pigment was dispersed below 1 μm after pump mixing, which proved the successful homogenization of the system components. In comparison, GPTES was slightly less effective in terms of the size of the aggregates observed, which was especially visible under mechanical stirring. *_i_*Bu_7_SSQ-OEt was highly effective at dispersing TiO_2_ even when the pigment was at the 2% loading. When compared to simple silane coupling agents (trialkoxysilanes), *_i_*Bu_7_SSQ-OEt does not have the ability to form gel products (polysilsesquioxanes), which, depending on the silane: filler ratio and processing conditions, may cause secondary aggregation of TiO_2_ by binding the particles together with polysilsesquioxane gel. SS-6GP-2TMOS effectively dispersed the pigment at 0.5% additive loading for both 1% and 2% of TiO_2_ used, while at the higher loading of silane agent, aggregation was observed due to the agent gelation. The gelling effect was more visible for SS-5GP-3TMOS, however the additive still allowed for elimination of larger (>10 μm) primary agglomerates. In addition, the dispersing effect of a selected modifier, *i*Bu_7_SSQ-OEt, was visualized with aid of digital optical microscopy (Figure 9), where diluted samples containing 0.0625% TiO_2_ (see *Section 3.4. Pigment Stability and Hiding Power Studies*) were observed in light transmission mode. For the samples containing modified TiO_2_, a significant drop in abundance of larger pigment agglomerates was observed, and in their place, even graininess effect was visible, which corresponded to the presence of finely dispersed pigment particles, as confirmed with detailed SEM imaging discussed above (for SEM images, see Supplementary Information Section 4: SEM images of the TiO_2_/EP composites).

### 3.4. Pigment Stability and Hiding Power Studies

**Figure 10:**
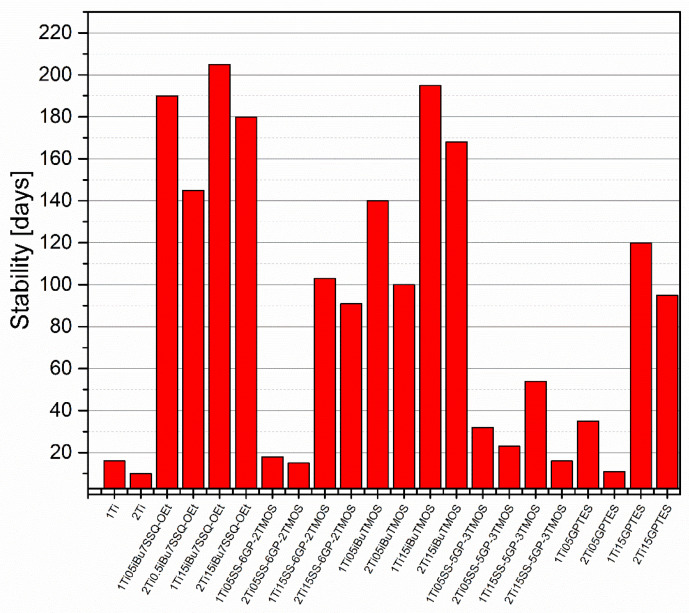
Time-dependent stability of TiO_2_ suspensions in liquid epoxy systems prepared by pump mixing.

**Figure 11:**
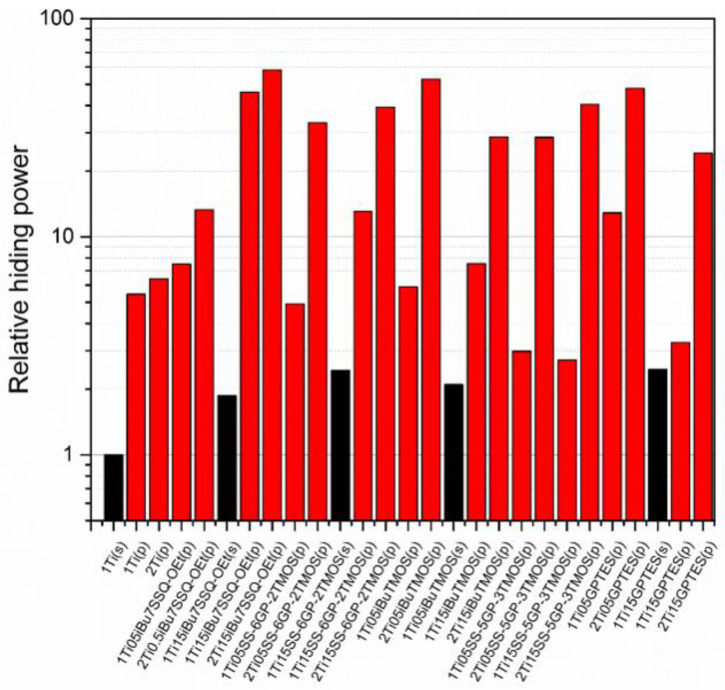
Relative hiding power of the pigmented epoxy systems.

The stability of modified and pristine TiO_2_/liquid epoxy resin suspensions was assessed by pouring samples of the suspensions in screw-capped centrifuge vials and leaving them on standing. Then, they were checked every couple of days for any signs of phase separation, including pigment precipitation on the vial walls or bottom, resin cloudiness, or discoloration near the surface. The samples prepared by mechanical stirring all underwent heavy phase separation within two weeks from preparation, as the larger agglomerates of TiO_2_ were characterized by poor stability in the suspension. It correlates with the SEM images (Supplementary Information), as these larger agglomerates were captured. On the other hand, the samples prepared by high shear pump mixing showed much higher suspension stability, the improved dispersing method increasing the stability time by over an order of magnitude for some of the studied systems (Figure 10). The main conclusion obvious from the results obtained is that, independently of the discussed TiO_2_ system used, increasing pigment loading from 1% to 2% resulted in more or less severe reduction in the suspension stability. It can be understood as higher concentration of TiO_2_ particles in the epoxy suspension causing their faster aggregation over storage time; however, the SEM images of TiO_2_/EP composites made from freshly prepared suspensions also revealed increased agglomeration in the composites containing 2% of TiO_2_. These results suggest that for the TiO_2_ pigments of limited stability, formation of primary agglomerates (<10 μm) is a fast process, after which these agglomerates grow at the rate depending on the pigment concentration in the suspension and the surface physicochemistry of the particles, up to the point of spontaneous sedimentation. This idea is further supported by the results of hiding power study (Figure 11). Interestingly, some of the studied coupling agents provided virtually no stabilization to the pigment over prolonged time, that is, SS-6GP-2TMOS at 0.5% loading and SS-5GP-3TMOS at both loadings. It may be due to the protic character of the diol groups generated during surface coupling of TiO_2_, resulting in interparticle attraction, but also less effective particle coating with the coupling agent and the gelling thereof, as discussed earlier (*Section 3.2. Surface Properties of the Obtained Modified TiO_2_ Pigments*). GPTES showed behaviour comparable to that of SS-6GP-2TMOS. On the other hand, *_i_*Bu_7_SSQ-OEt at 0.5% loading provided stabilization superior to all the other coupling agents studied, and at 1.5% loading, slightly higher than that of *i*BuTMOS. It is due to the apolar character of the isobutyl group, which both the SSQ and silane compounds share in common.

The studies of hiding power based on the samples’ visible light transmittance revealed the effect of both processing technique and coupling agent choice on the pigmenting efficacy of the resulting epoxy systems. While application of modified TiO_2_ via mechanical stirring resulted in slight improvement of hiding power (approximately two-fold on the average of the studied systems), and the implementation of pump mixing allowed for a more significant improvement (over five-fold for neat TiO_2_ at 1% loading), the most impressive results were obtained when both surface treatment and pump mixing were applied. It proves that chemical modification of the pigment particles results not only in enhanced dispersability thereof in the epoxy during suspension preparation, but also stabilizes these particles in the suspension, hampering their secondary self-aggregation. For neat TiO_2_, increasing the pigment loading from 1% to 2% resulted in only 17% increase of hiding power, making little change in the overall performance of the composition. On the other hand, for the systems containing TiO_2_ treated with 0.5% of *_i_*Bu_7_SSQ-OEt it was 59% increase; for 0.5% and 1.5% of SS-6GP-2TMOS, 679% and 300%, respectively; for 0.5% and 1.5% of SS-5GP-3TMOS, 958% and 1490%, respectively. Interestingly, despite showing moderate to poor stability over storage time, the discussed spherosilicate-based modifiers still performed well at enhancing pigmentation efficacy of the TiO_2_ treated thereof. The best performing pigment system was pump-mixed TiO_2_ treated with 1.5% of *i*Bu_7_SSQ-OEt, where at 1% TiO_2_ loading, the relative hiding power was over ten times higher than that of the sample containing pump-mixed neat TiO_2_; at 2% loading, the hiding power was nine times higher than that of the neat pigment counterpart. 

**Figure 12:**
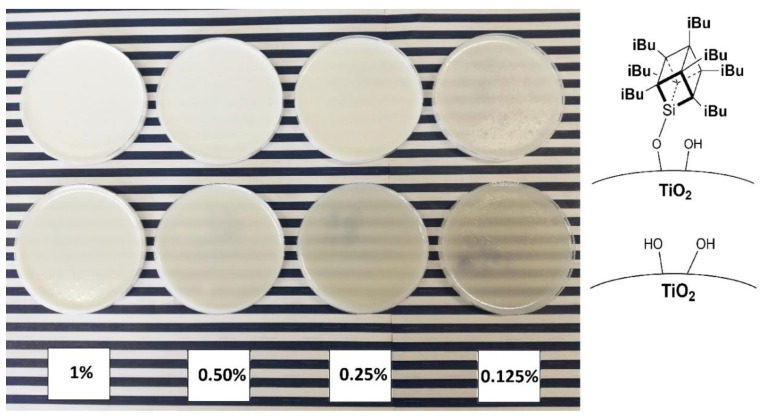
Serial dilution samples of mixing pump-prepared 1.5% *i*Bu_7_SSQ-OEt TiO_2_/EP (top) and neat TiO_2_/EP (bottom).

To visualise the practical effect of improved TiO_2_ dispersion within the epoxy base, an additional experiment was performed where a sample of a chosen epoxy containing 1% of modified TiO_2_ and a series of dilutions was prepared with neat epoxy to obtain samples of 0.5%, 0.25%, 0.125%, and 0.0625% of TiO_2_ (Figure 12, the last concentration omitted due to poor pigmentation). After that, pigmentation was evaluated comparatively between the series of samples by assessing their visual opaqueness on a colourful background. While the samples containing neat TiO_2_ were already visibly transparent at 0.5% loading, the samples containing 1.5% *i*Bu_7_SSQ-OEt-modified TiO_2_ provided satisfactory pigmentation up to the 0.25% dilution.

### 3.5. Mechanical Studies

**Figure 13:**
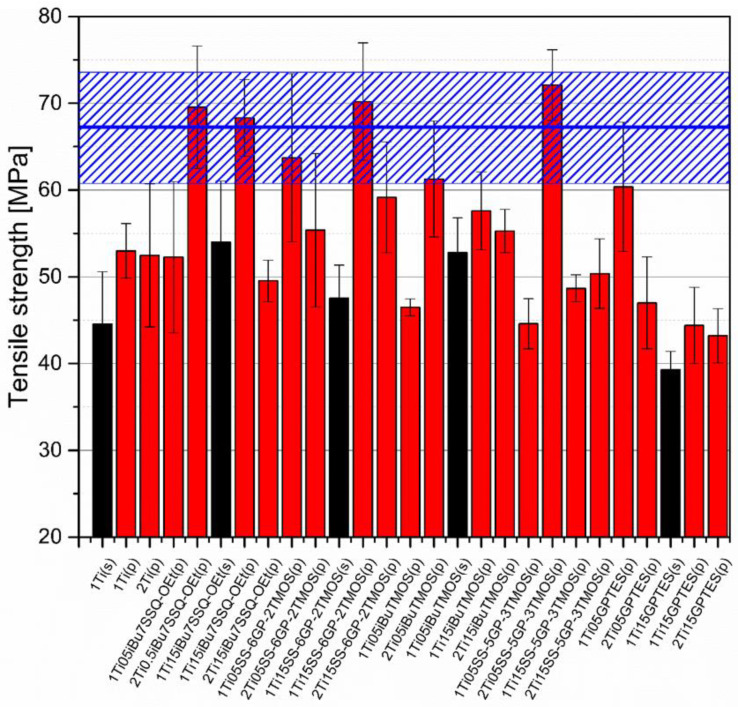
Tensile strength of neat and modified TiO_2_/EP composites.

**Figure 14:**
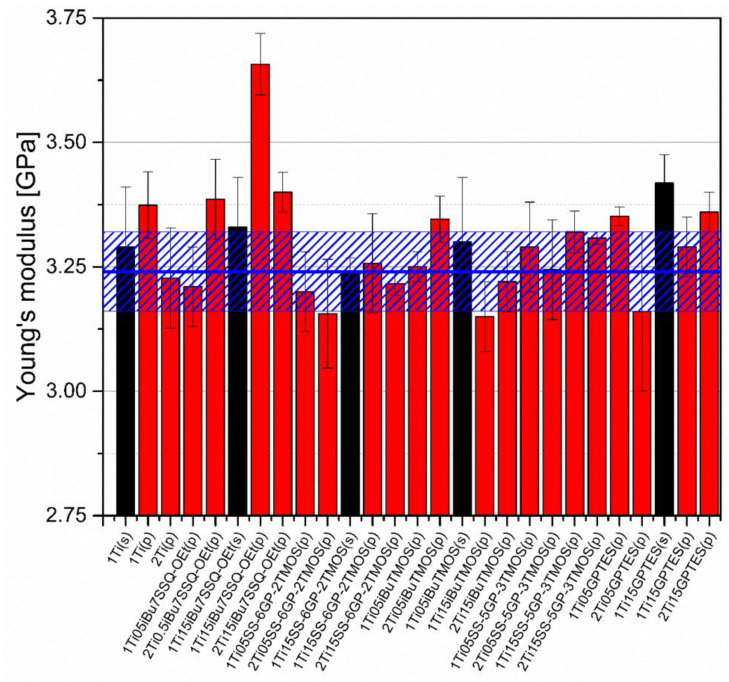
Young’s modulus of neat and modified TiO_2_/EP composites.

**Figure 15:**
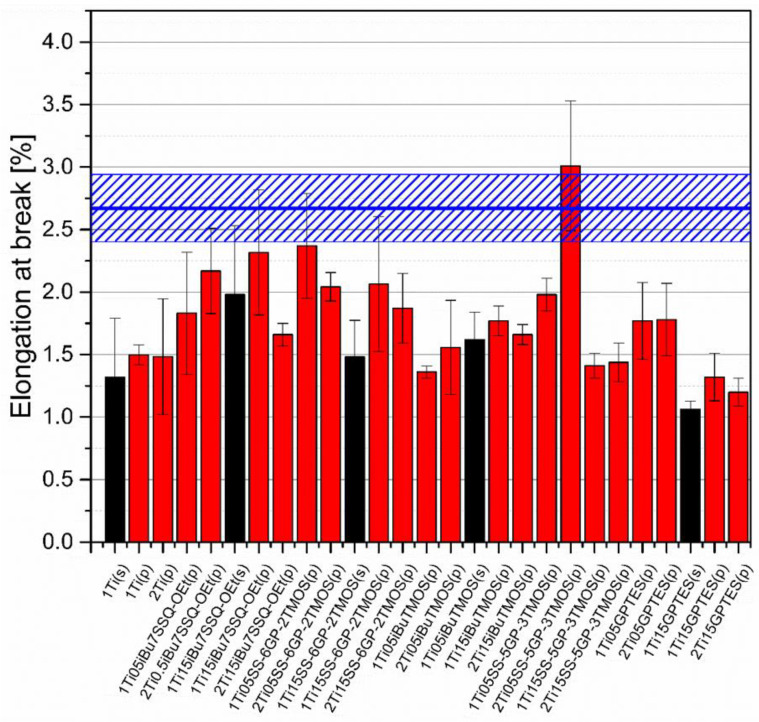
Elongation at break of neat and muodified TiO_2_/EP composites.

**Figure 16:**
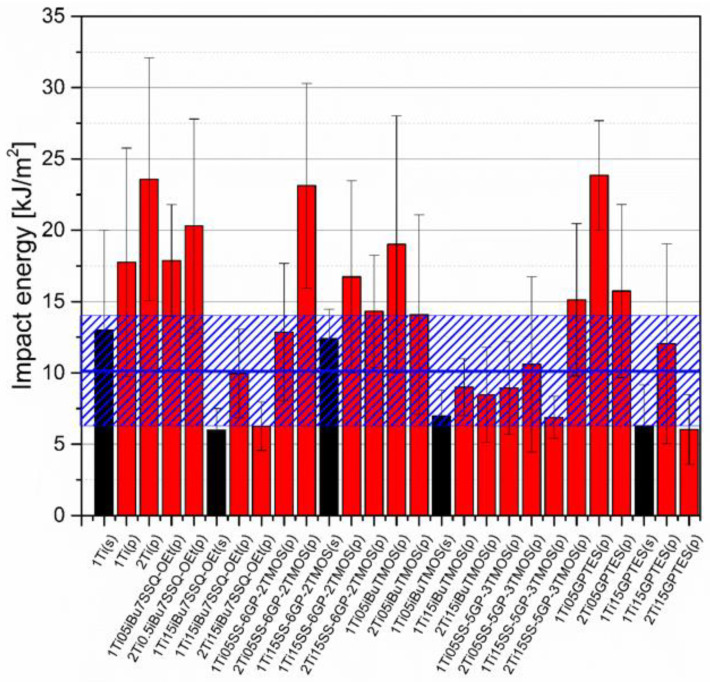
Impact energy of neat and modified TiO_2_/EP composites.

Mechanical analysis revealed subtle differences between the TiO_2_/epoxy systems studied, even despite low loading of TiO_2_ in the composites (Figure 13, Figure 14, Figure 15 and Figure 16). For Young’s modulus, it was observed that the systems present stiffness comparable to the neat epoxy within the limits of the standard deviation (Figure 14). Also, for the samples containing either 1% or 2% of the same type of prepared TiO_2_ (either pristine or modified), there was a tendency towards Young’s modulus increase along with the pigment concentration for well-dispersed systems (e.g., 0.5% *_i_*Bu_7_SSQ-OEt/TiO_2_, 0.5% *i*BuTMOS/TiO_2_), while for the agglomerating pigments, the value would drop (e.g., pristine TiO_2_, 0.5% GPTES/TiO_2_). Due to low loadings of the pigmenting filler used, retention of tensile properties (tensile strength and elongation at break, that is) should rather be considered instead of their improvement, as the loading of the filler is too small to provide enough reinforcement and the mechanical failure may propagate throughout the bulk polymer. For tensile strength, mechanical stirrer-prepared composites were the weakest from each series, as the agglomerated particles provided spots of structural imperfection, causing material failure. Some of the highest values of tensile strength and elongation at break were recorded for systems loaded with TiO_2_ modified with 1.5% of SS-6GP-2TMOS and 0.5% of SS-5GP-3TMOS due to high dispersion and well-developed particle-polymer interphase. Also, *_i_*Bu_7_SSQ-OEt/TiO_2_, performed slightly better than its silane counterpart system, iBuTMOS/TiO_2,_ suggesting that silsesquioxane- and spherosilicate-based coupling agents provide better surface treatment agents, likely due to less gelation side reactions.

Impact resistance tests revealed that some of the prepared systems were characterized by up to over 100% increase in durability described by the mean impact energy (Figure 16). Among those, samples containing pristine TiO_2_ showed satisfactory performance and that the systems of the highest dispersion of the pigment are not the ones of the highest impact resistance. This proves that the agglomerates and particles of moderate size may work as crack propagation interrupters. Findings supporting such statement were reported earlier when studying diatomite-filled epoxy composites [40]. Triethylenetetramine-cured epoxy resins under low temperatures and high strain rates tend to undergo continuous crack propagation and such heterogeneous regions introduced in the polymer bulk may help to discontinue this propagation [41].

### 3.6. Thermal Studies

**Figure 17:**
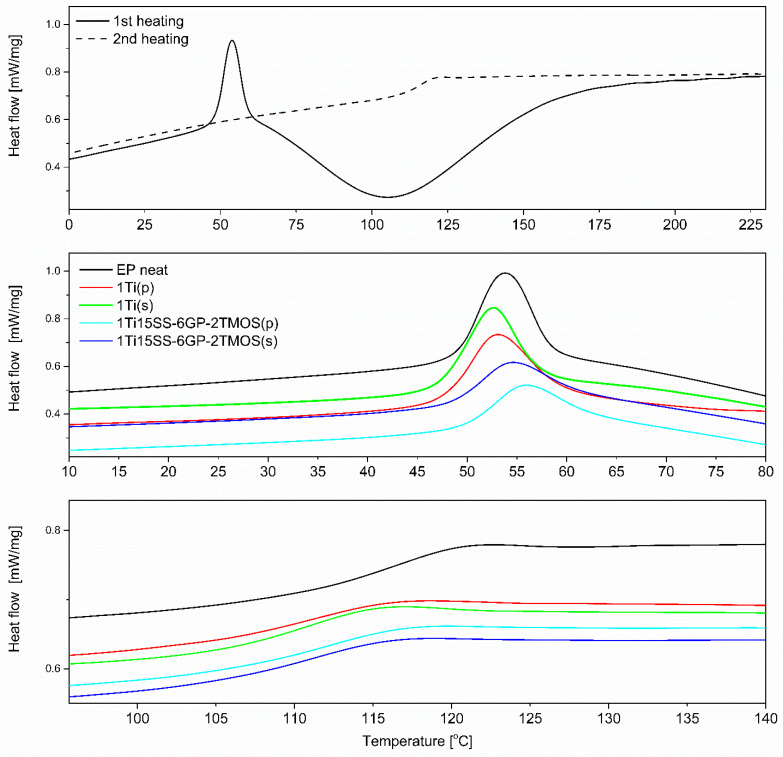
DSC plots of selected epoxy systems; neat EP (**top**); T_gu_ region (**middle**), T_g_ region (**bottom**).

Although the studied materials share the same polymer matrix and the base pigmenting filler, the preparation methodology applied and the coupling agent used cause subtle structural and interfacial differences that can be elucidated with DSC (Figure 17, top). Epoxy systems of similar polymer matrix were studied earlier [40,42]. It can be seen that for the second heating cycle, the T_g_ of most of the samples appears at ~120 °C, being close to that of the neat epoxy (Figure 17, bottom). However, for the mechanical stirrer-prepared compositions, the T_g_ was observed to be slightly lowered for all examples, both compared to the mixing pump-prepared ones and the neat epoxy. It has been a subject of discussion that fillers presenting weak interaction with the polymer tend to reduce T_g_ by creating an interface where due to poor filler surface wetting action, a fraction of polymer is formed, characterized by more freedom than that of the bulk polymer [43,44,45]. By this thesis, it can be explained that the poorly dispersed TiO_2_ particles create such interface due to their reduced surface/volume ratio. Another observation was made on the change of T_g_ over time. Two measurements were taken 21 days apart, after seven and 30 days of resin casting. By the time of the second measurement, T_g_ would increase by average of two degrees Celsius for most samples, as additional diffusion between the epoxy and TiO_2_ occurred over time. The effect was less prominent for the samples containing GPTES-treated TiO_2_, suggesting stronger and less elastic grafting of the epoxy chains on the modified particles in the first place. Similar behaviour of nanocomposites containing fillers with reactive group-containing coupling agents was described by Ash, Schadler et al. [45]. Additionally, the glass transition of epoxy composites with no thermal treatment was studied (glass transition of residually uncured resin, T_gu_, Figure 17, middle). In this case, the phenomenon of low-temperature glass transition is caused mostly by the bulk resin, with polymer domains containing highly flexible chains containing either unreacted oxirane or amine groups. These chains undergo further partial cross-linking upon sample aging, but for more complete curing, samples need to be heated above that T_gu_ temperature for the unreacted chains to regain mobility and undergo further cross-linking. As was mentioned, this phenomenon is caused by the bulk material and by so, the filler has a very small and unclear effect on the T_gu_ values. For the studied systems, T_gu_ was in a 52–54 °C range for all the samples after seven days from casting, and after 30 days the value would increase by average of 6 °C. The highest ΔT_gu_ were observed for the samples containing TiO_2_ treated with 1.5% of GPTES and spherosilicate agents, and prepared by mixing pump, supporting the abovementioned hypothesis that the well-dispersed, modified nanoparticles undergo grafting of epoxy chains on their surface, which introduces regions of decreased chain mobility within the matrix. The effect was less prominent for the remaining modifiers, suggesting more mobile nanoparticle-matrix interphase caused by the non-covalent interactions.

## 4. Conclusions

The conclusions to be drawn from this study, are:

(1) A novel method for preparation of highly dispersed TiO_2_/epoxy systems with the aid of a custom high-shear gear pump was presented. The impact of the pigment dispersing method on the properties of epoxy systems has been discussed in detail, including suspension stability and composite hiding power. It was shown that physical means of dispersing the nanoparticles are as much important as the chemical ones for their surface treatment, and together, a synergistic effect is obtained.

(2) An effective and green method for TiO_2_ surface treatment in aqueous media was described. Herein the method may be introduced into the production line of TiO_2_ pigments for coating/paint industry, if professional application of such systems was to be considered.

(3) Improved mechanical properties were observed in terms of impact resistance and Young’s modulus, when silsesquioxane coupling agent was used, due to good dispersion of the nanoparticles within the epoxy matrix.

(4) High hiding power was obtained with the described methodology including both chemical surface treatment of the TiO_2_ pigment and high-shear dispersing procedure thereof, proving the synergistic effect of the two. The proposed methodology may serve for preparation of novel, high-performance coating systems. Additionally, improved dispersion and particle stability may allow for reduction of pigment loading for the epoxy base to obtain the satisfactory hiding power.

(5) TiO_2_ dispersions of long shelf life were obtained, the selected cage siloxane additives showing superior stabilizing effect over silane coupling agents.

(6) Superhydrophobic effect for TiO_2_ surface was obtained for two coupling agents—*_i_*BuTMOS and *_i_*Bu_7_SSQ-OEt, showing the great possibilities in terms of modification of the surface properties of TiO_2_ with the application of silsesquioxane agents.

(7) The chemical reactions occurring during TiO_2_ surface modification were proposed and confirmed with FT-IR. The hydrolytic opening of oxirane ring to the corresponding diol has been confirmed, possibly catalysed by TiO_2_ itself, which is an important finding concerning surface treatment of similar materials in water-based media.

The obtained findings show that the silsesquioxane- and spherosilicate-based coupling agents are somehow similar to their silane counterparts, but have their advantages over simple silanes. It might encourage tuning of silsesquioxane compounds structure and the process of their application, if their use as surface treatment agents is sought for special applications, e.g., high performance coating systems.

## Figures and Tables

**Table 1:** Silsesquioxane, spherosilicate, and silane coupling agents used in this study.

Name	Abbreviation
Isobutyltrimethoxysilane	*i*BuTMOS
3-glycidoxypropyltriethoxysilane	GPTES
ethoxyhepta(isobutyl)octasilsesquioxane	*i*Bu_7_SSQ-OEt
hexa(3-glycidoxypropyl)di(trimethoxysilylethyl)octaspherosilicate	SS-6GP-2TMOS
penta(3-glycidoxypropyl)tri(trimethoxysilylethyl)octaspherosilicate	SS-5GP-3TMOS

**Table 2:** The TiO_2_/epoxy resin composites prepared in this study.

Sample Name	Organosilicon Coupling Agent Type	Organosilicon Coupling Agent Amount [%] ^1^	TiO_2_ Amount [%]	Mixing Method	Sample Code
1	none	-	1	Mechanical stirrer	1Ti(s)
2	none	-	1	Mixing pump	1Ti(p)
3	none	-	2	Mixing pump	2Ti(p)
4	*i*BuTMOS	0.5	1	Mixing pump	1Ti05*i*BuTMOS(p)
5	*i*BuTMOS	0.5	2	Mixing pump	2Ti05*i*BuTMOS(p)
6	*i*BuTMOS	1.5	1	Mechanical stirrer	1Ti15*i*BuTMOS(s)
7	*i*BuTMOS	1.5	1	Mixing pump	1Ti15*i*BuTMOS(p)
8	*i*BuTMOS	1.5	2	Mixing pump	2Ti15*i*BuTMOS(p)
9	GPTES	0.5	1	Mixing pump	1Ti05GPTES(p)
10	GPTES	0.5	2	Mixing pump	2Ti05GPTES(p)
11	GPTES	1.5	1	Mechanical stirrer	1Ti15GPTES(s)
12	GPTES	1.5	1	Mixing pump	1Ti15GPTES(p)
13	GPTES	1.5	2	Mixing pump	2Ti15GPTE (p)
14	*i*Bu_7_SSQ-OEt	0.5	1	Mixing pump	1Ti05*i*Bu_7_SSQ-OEt(p)
15	*i*Bu_7_SSQ-OEt	0.5	2	Mixing pump	2Ti05*i*Bu_7_SSQ-OEt(p)
16	*i*Bu_7_SSQ-OEt	1.5	1	Mechanical stirrer	1Ti15*i*Bu_7_SSQ-OEt(s)
17	*i*Bu_7_SSQ-OEt	1.5	1	Mixing pump	1Ti15*i*Bu_7_SSQ-OEt(p)
18	*i*Bu_7_SSQ-OEt	1.5	2	Mixing pump	2Ti15*i*Bu_7_SSQ-OEt(p)
19	SS-6GP-2TMOS	0.5	1	Mixing pump	1Ti05 SS-6GP-2TMOS (p)
20	SS-6GP-2TMOS	0.5	2	Mixing pump	2Ti05 SS-6GP-2TMOS (p)
21	SS-6GP-2TMOS	1.5	1	Mechanical stirrer	1Ti15 SS-6GP-2TMOS (s)
22	SS-6GP-2TMOS	1.5	1	Mixing pump	1Ti15 SS-6GP-2TMOS (p)
23	SS-6GP-2TMOS	1.5	2	Mixing pump	2Ti15 SS-6GP-2TMOS (p)
24	SS-5GP-3TMOS	0.5	1	Mixing pump	1Ti05 SS-5GP-3TMOS (p)
25	SS-5GP-3TMOS	0.5	2	Mixing pump	2Ti05 SS-5GP-3TMOS (p)
26	SS-5GP-3TMOS	1.5	1	Mixing pump	1Ti15 SS-5GP-3TMOS (p)
27	SS-5GP-3TMOS	1.5	2	Mixing pump	2Ti15 SS-5GP-3TMOS (p)

^1^ Silane coupling agent amount is given in correspondence to TiO_2_, as *w*/*w* % of TiO_2._

**Table 3:** Water contact angles of TiO_2_ modified with the studied silane coupling agents.

Silane Coupling Agent Type	Silane Coupling Agent Amount [%] ^1^	Water Contact Angle [°]
None	-	0
*_i_*BuTMOS	0.5	0
*_i_*BuTMOS	1.5	Superhydrophobic ^2^
GPTES	0.5	0
GPTES	1.5	0
*_i_*Bu_7_SSQ-OEt	0.5	0
*_i_*Bu_7_SSQ-OEt	1.5	Superhydrophobic ^2^
SS-6GP-2TMOS	0.5	0
SS-6GP-2TMOS	1.5	0
SS-5GP-3TMOS	0.5	0
SS-5GP-3TMOS	1.5	0

^1^ Silane coupling agent amount is given in correspondence to TiO_2_, as *w/w* % of TiO_2_; ^2^ roll-off observed at each attempt at measuring the contact angle.

## Data Availability

All the data were collected and provided either in the main manuscript file or in the Supplementary Materials File.

## Supplementary Materials

The following are available online at https://www.mdpi.com/article/10.3390/ma15020494/s1, Figure S1. Internal gear pump setup used for preparation of TiO2/epoxy dispersions; Figure S2. A sample of cured TiO_2_/epoxy composite prepared; NMR spectroscopy of the obtained compounds; MALDI-TOF mass spectra of obtained spherosilicate compounds; SEM images of the TiO_2_/EP composites.
