# Supplementary material for: Where ppm Quantities of Silsesquioxanes Make a Difference—Silanes and Cage Siloxanes as TiO2 Dispersants and Stabilizers for Pigmented Epoxy Resins
Source: Materials (Basel). 2022 Jan 10;15(2):494. doi: 10.3390/ma15020494 (PMC8779391; doi:10.3390/ma15020494)
Supplement: Supplementary file 1 [file materials-15-00494-s001.zip › materials-1481178-supplementary.pdf]

materials MDPI

Supplementary Information

Where ppm Quantities of Silsesquioxanes Make a Difference—Silanes and Cage Siloxanes as TiO_2 Dispersants and Stabilizers for Pigmented Epoxy Resins

Dariusz Brząkalski [1], Robert E. Przekop [2,*], Miłosz Frydrych [1], Daria Pakuła [1], Marta Dobrosielska [3], Bogna Sztorch [2], Bogdan Marciniec [1,2,*]

1 Faculty of Chemistry, Adam Mickiewicz University in Poznań, 8 Uniwersytetu Poznańskiego, 61−614 Poznań, Poland; d.brzakalski@gmail.com (D.B.), frydrych@amu.edu.pl (M.F.); darpak@amu.edu.pl (D.P.), bogdan.marciniec@amu.edu.pl (B.M.)
2 Centre for Advanced Technologies, Adam Mickiewicz University in Poznań, 10 Uniwersytetu Poznańskiego, 61-614 Poznań, Poland; rprzekop@amu.edu.pl (R.E.P.), bogna.sztorch@amu.edu.pl, bogdan.marciniec@amu.edu.pl (B.M.)
3 Faculty of Materials Science and Engineering, Warsaw University of Technology, 141 Wołoska, 02-507 Warsaw, Poland; Marta.Dobrosielska@pw.edu.pl (M.D.);
* Correspondence: r.przekop@gmail.com, rprzekop@amu.edu.pl (R.E.P.), bogdan.marciniec@amu.edu.pl (B.M.)

Table of contents

1. Pictures of the equipment and samples 2
2. NMR spectroscopy of the obtained compounds 3
3. MALDI-TOF mass spectra of obtained spherosilicate compounds 9
   SS-6GP-2TMOS 9
   SS-5GP-3TMOS 14
4. SEM images of the TiO_2/EP composites 19

1. Pictures of the equipment and samples

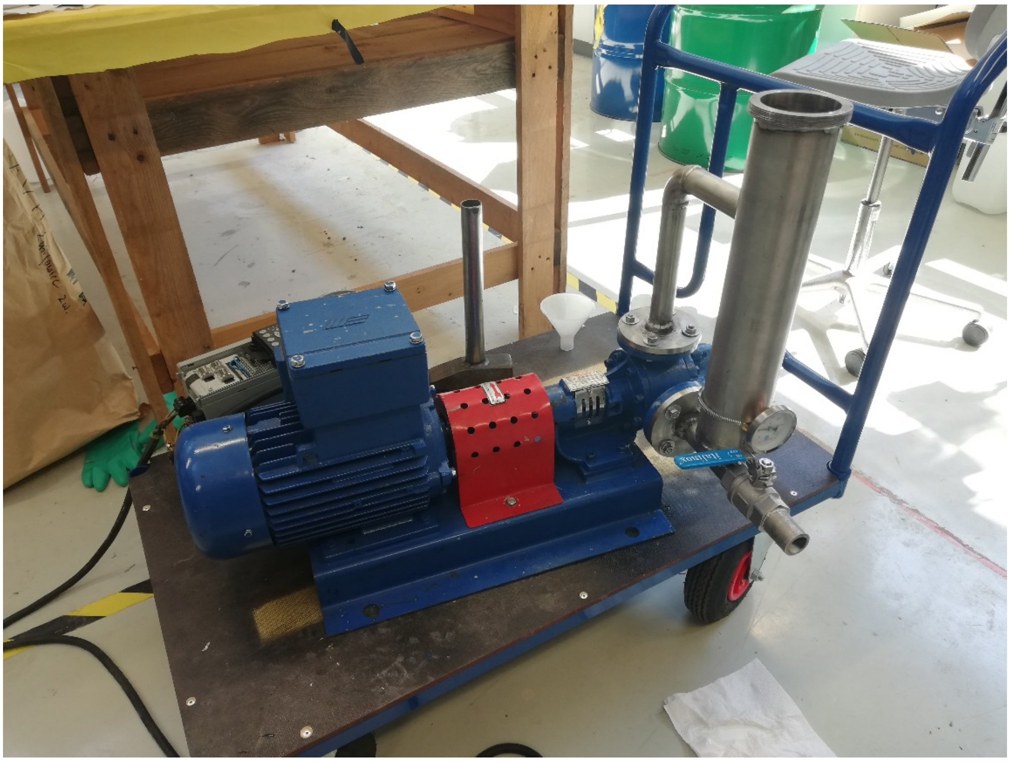

Figure S1. Internal gear pump setup used for preparation of TiO_2/epoxy dispersions

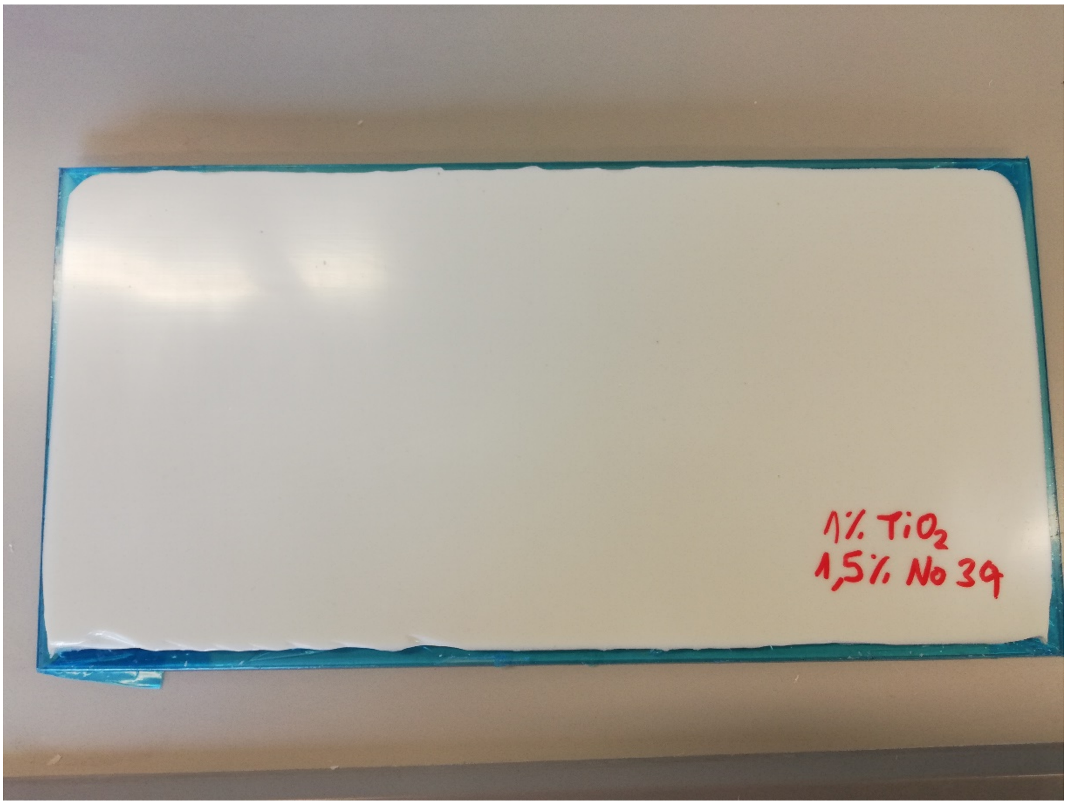

Figure S2. A sample of cured TiO_2/epoxy composite prepared

2. NMR spectroscopy of the obtained compounds

1,3,5,7,9,11,13,15-hexa((3-glycidoxypropyl)dimethylsiloxy)-di((trimethoxysilyl)dimethylsilloxy) pentacyclo[9.5.1.1^{3,9}.1^{5,15}.1^{7,13}]octasiloxane (SS-6GP-2TMOS)

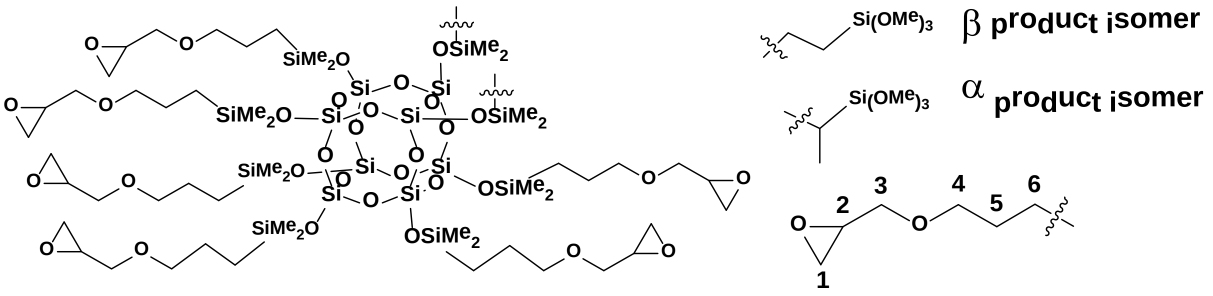

^{1}H NMR (400 MHz, $CDCl_3$): δ (ppm) = 3.70-3.67 (m, 6H position 3), 3.55 (s, 18H, OMe), 3.48-3.40 (m, 12H, position 4), 3.39-3.36 (m, 6H, position 3), 3.14-3.11 (m, 6H, position 2), 2.79-2.77 (m, 6H, position 1), 2.60-2.59 (m, 6H, position 1), 1.65-1.60 (m, 12H, position 5), 1.11 (d, alpha product $-CH_3$), 0.62-0.59 (m, 20H, Si<u>CH_2CH_2</u>Si, position 6), 0.14 (s, 48H, $SiMe_2$); **α** and **β** isomers were obseved in 8 : 92 ratio;

^{13}C NMR (101 MHz, $CDCl_3$): δ (ppm) = 74.25, 71.59, 71.58 (glycidoxy group), 50.97, 50.78, 50.69 (OMe), 44.46 (propyl-3-CH_2), 23.32, 13.81, 13.80 (propyl-1 and propyl-2-CH_2), 8.62, 8.60 (Si-<u>CH_2CH_2</u>-Si), 7.41, 5.29 (Si<u>$CH(CH_3)$</u>Si), 0.44 (Si-<u>CH_2CH_2</u>-Si), -0.25, -0.28, -0.31 (glycidoxypropyl $SiMe_2$), -0.95, -0.98, -1.01 (trimethoxysilylethyl $SiMe_2$);

^{29}Si NMR (79,5 MHz, $CDCl_3$): δ (ppm) = 13.23-13.18 (Si-trimethoxysilylethyl), 12.97-12.91 (Si-glycidyloxypropyl), -41.67 ($Si(OMe)_3$), -109.09 (core).

^{1}H NMR

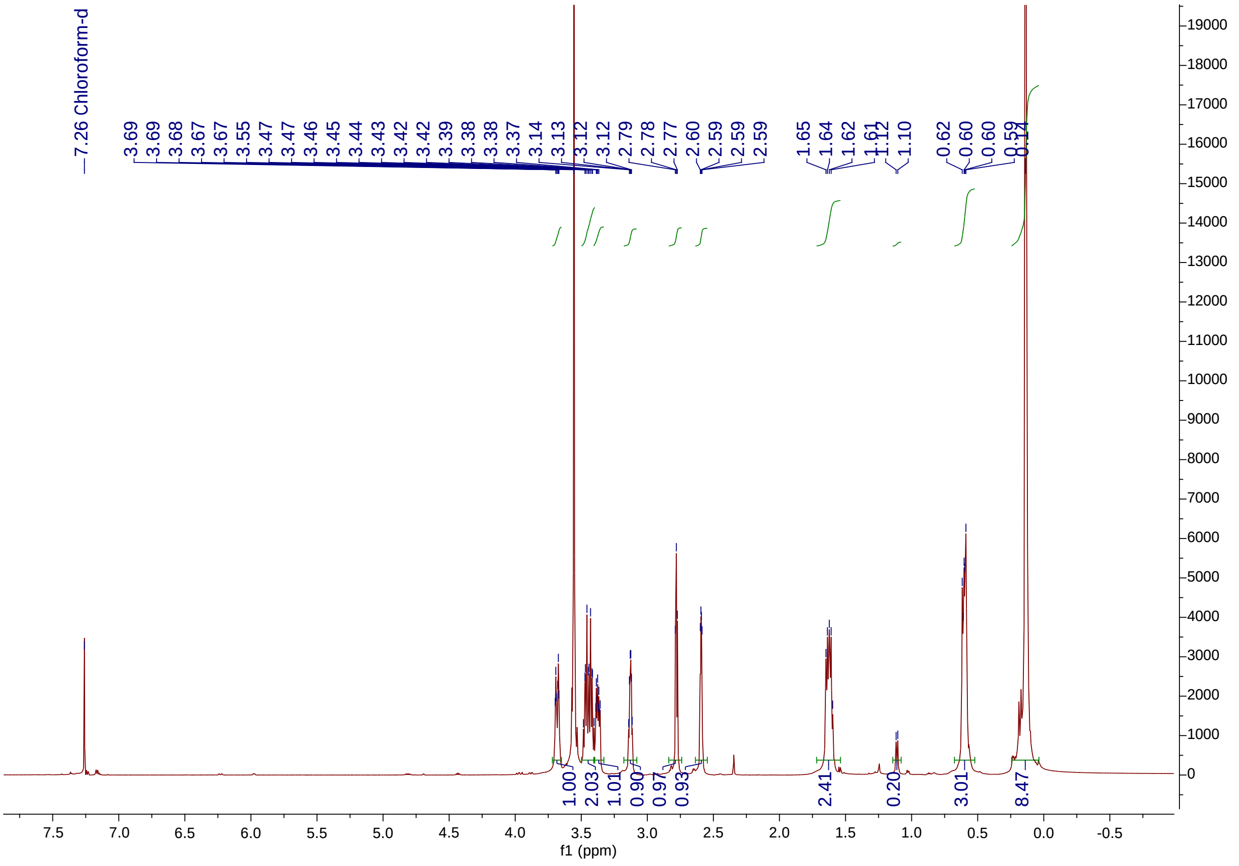

^{13}C NMR

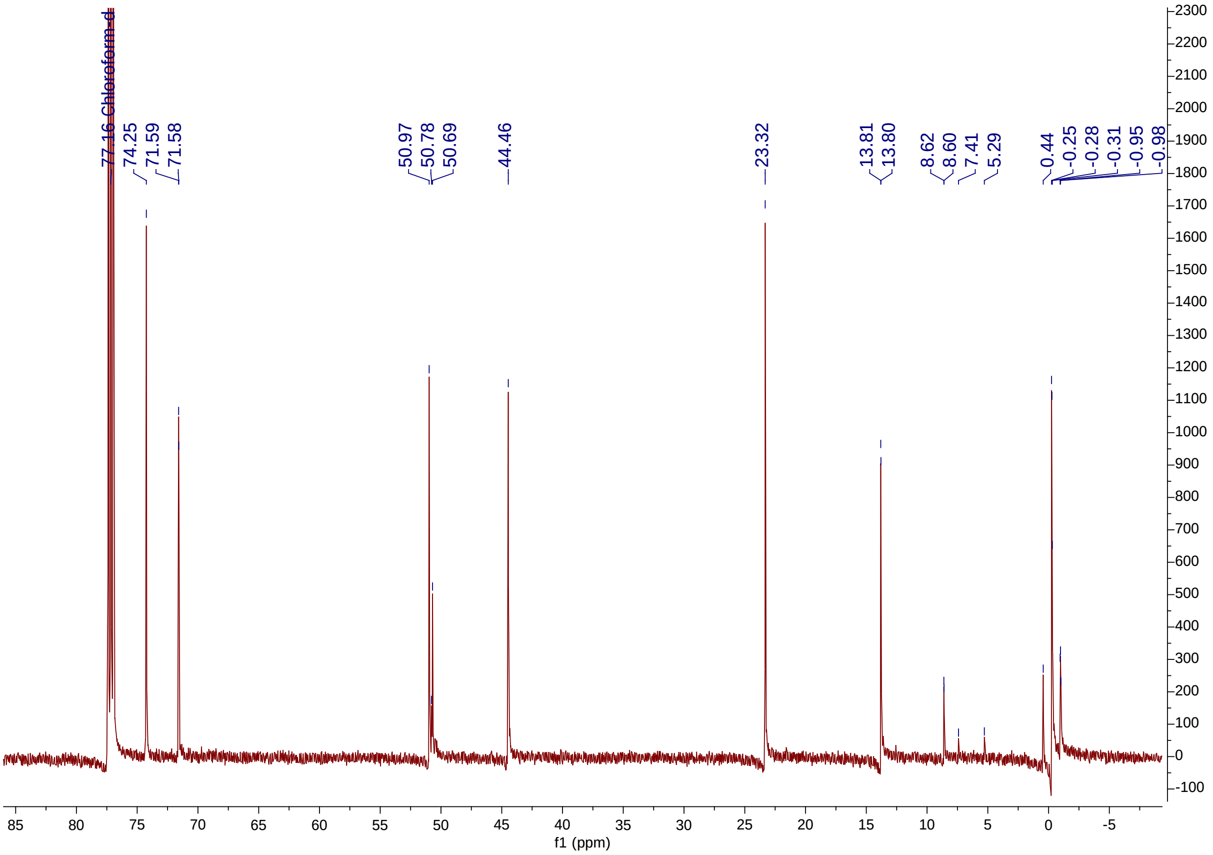

^{29}Si NMR

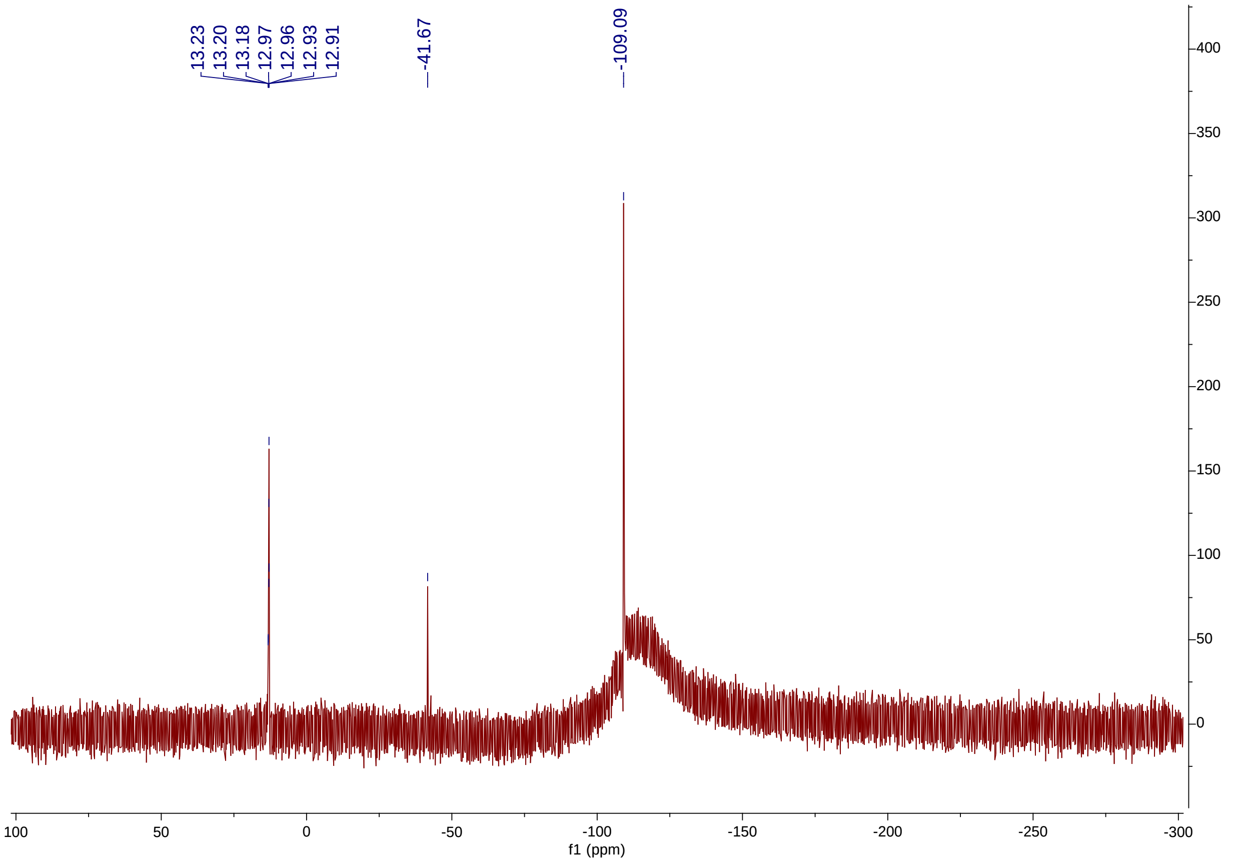

1,3,5,7,9,11,13,15-penta((3-glycidoxypropyl)dimethylsiloxy)-tri((trimethoxysilyl)dimethylsilloxy) pentacyclo[9.5.1.1^{3,9}.1^{5,15}.1^{7,13}]octasiloxane (SS-5GP-3TMOS)

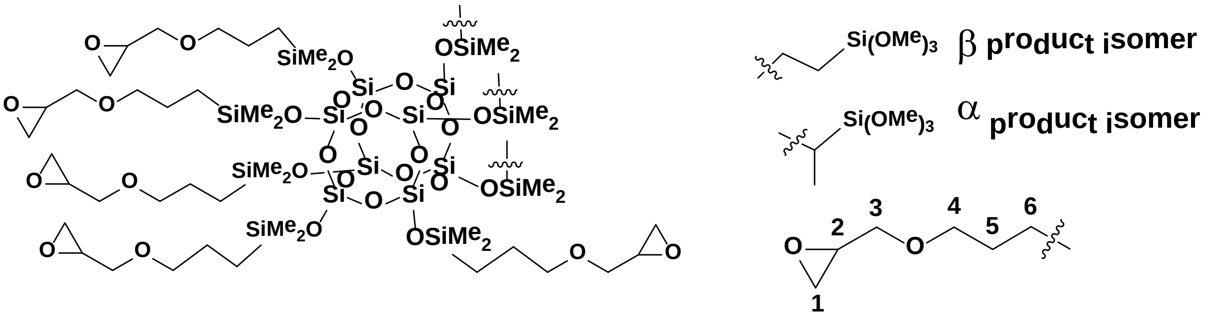

^{1}H NMR (400 MHz, $CDCl_3$): δ (ppm) = 3.70-3.66 (m, 5H position 3), 3.54 (s, 27H, OMe), 3.47-3.43 (m, 10H, position 4), 3.39-3.33 (m, 5H, position 3), 3.14-3.09 (m, 5H, position 2), 2.77-2.74 (m, 5H, position 1), 2.59-2.55 (m, 5H, position 1), 1.64-1.63 (m, 10H, position 5), 1.12 (d, alpha product $-CH_3$), 0.61-0.59 (m, 22H, Si$\underline{CH_2CH_2}$Si, position 6), 0.14 (s, 48H, $SiMe_2$); **α** and **β** isomers were obseved in 10 : 90 ratio;
^{13}C NMR (101 MHz, $CDCl_3$): δ (ppm) = 73.87, 71.31 (glycidoxy group), 50.61, 50.32 (OMe), 43.99 (propyl-3-CH_2), 23.06, 13.55 (propyl-1 and propyl-2-CH_2), 8.36 (Si-$\underline{CH_2CH_2}$-Si), 7.14, 5.01 (Si$\underline{CH(CH_3)}$Si), 0.17 (Si-$\underline{CH_2CH_2}$-Si), -0.54, -0.57, -0.61 (glycidoxypropyl $SiMe_2$), -1.24, -1.27 (trimethoxysilylethyl $SiMe_2$);
^{29}Si NMR (79,5 MHz, $CDCl_3$): δ (ppm) = 13.17-13.13 (Si-trimethoxysilylethyl), 12.92-12.87 (Si-glycidyloxypropyl), -41.87 ($Si(OMe)_3$), -109.12 (core).

^{1}H NMR

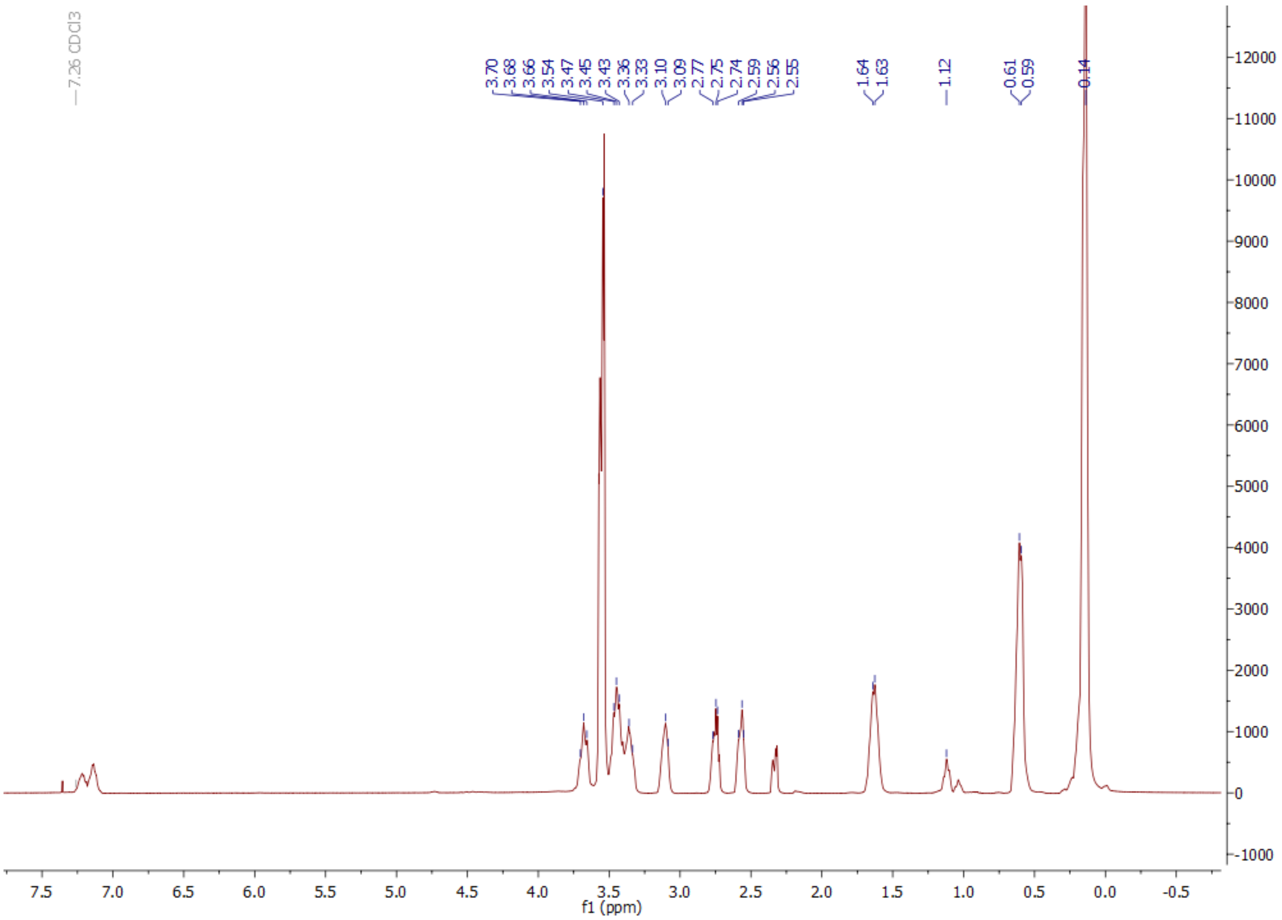
7.26 CDCl3
3.70
3.68
3.66
3.54
3.47
3.45
3.43
3.36
3.33
3.10
3.09
2.77
2.75
2.74
2.59
2.56
2.55
1.64
1.63
1.12
0.61
0.59
0.14
f1 (ppm)

^{13}C NMR

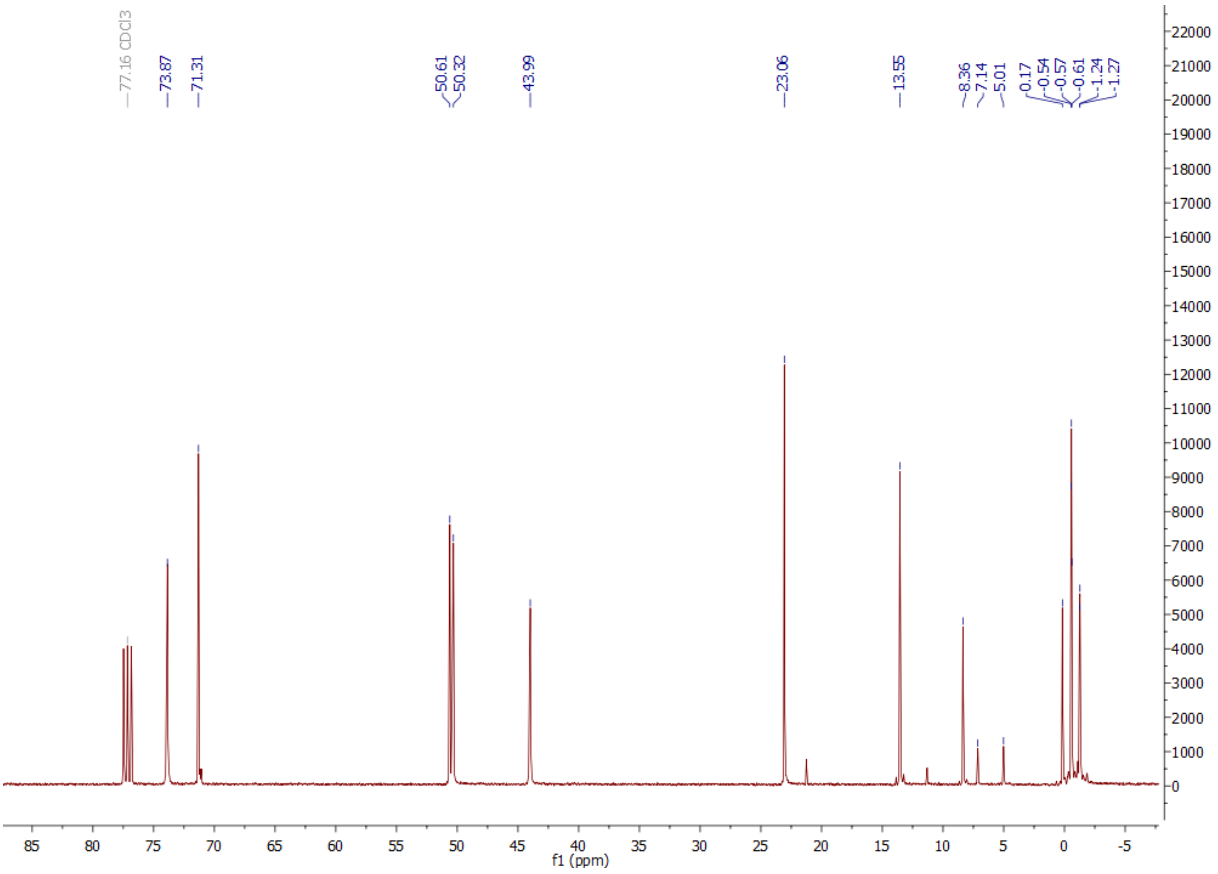
77.16 CDCl3
73.87
71.31
50.61
50.32
43.99
23.06
13.55
8.36
7.14
5.01
0.17
-0.54
-0.57
-0.61
-1.24
-1.27
f1 (ppm)

^{29}Si NMR

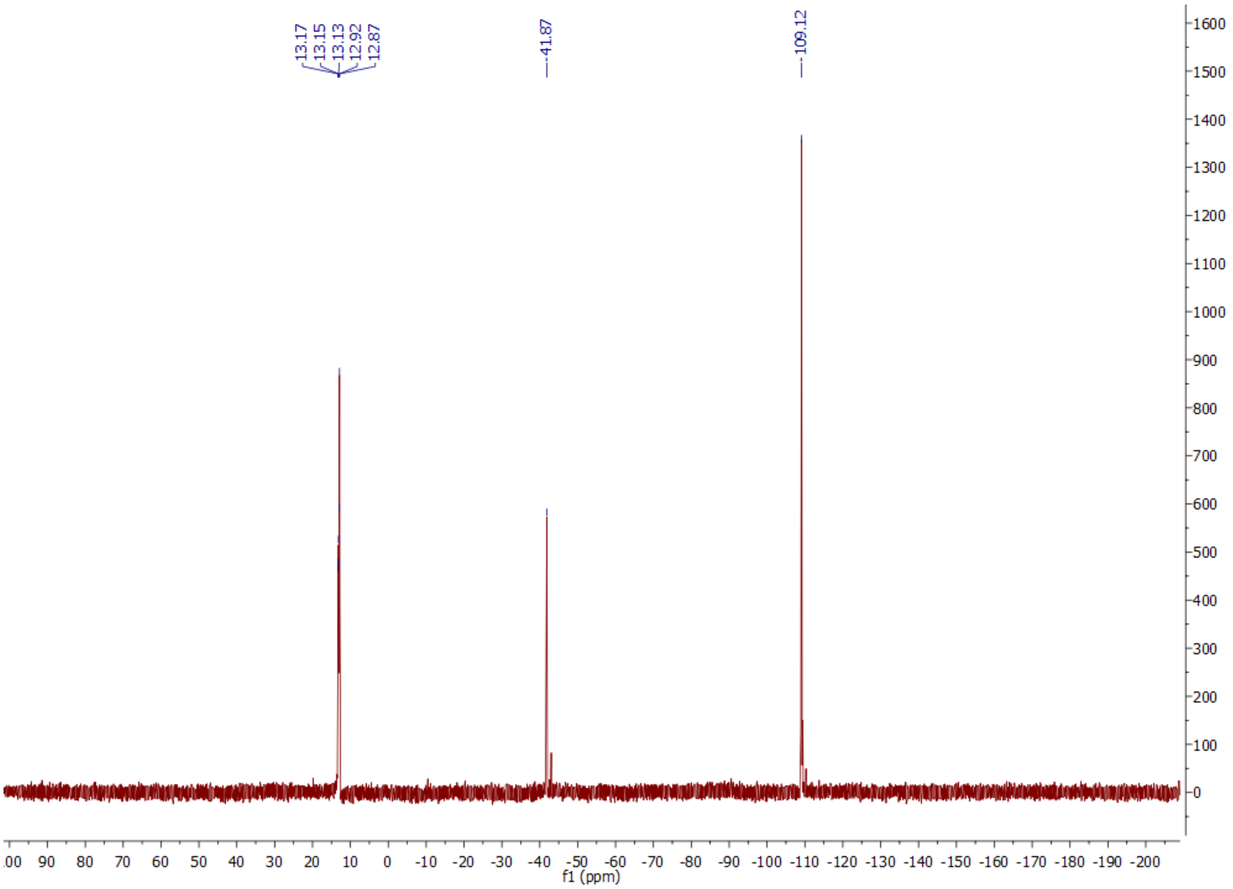
13.17
13.15
13.13
12.92
12.87
-41.87
-109.12
f1 (ppm)

1-ethoxy-3,5,7,9,11,13,15-heptaisobutylpentacyclo[9.5.1.1^{3,9}.1^{5,15}.1^{7,13}]octasiloxane (iBu_7SSQ-OEt)

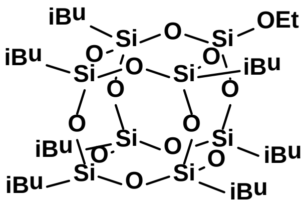

^{1}H NMR (400 MHz, $CDCl_3$): δ (ppm) = 3.84 (q, J = 7.0 Hz, 2H, $OC\underline{H_2}CH_3$), 1.92-1.80 (m, 7H, iBu), 1.23 (t, J = 7.0 Hz, 3H, $OCH_2C\underline{H_3}$), 0.97-0.95 (m, 42H, iBu), 0.64-0.59 (m, 14H, iBu);

^{13}C NMR (101 MHz, $CDCl_3$): δ (ppm) = 59.48 ($O\underline{C}H_2CH_3$), 25.85, 25.83, 24.02, 23.98, 22.64, 22.59, 22.51, 22.49 (iBu), 18.03 ($OCH_2\underline{C}H_3$);

^{29}Si NMR (79,5 MHz, $CDCl_3$): δ (ppm) = -66.71, -66.94, -67.87 (cage), -103.22 (SiOEt).

^{1}H NMR

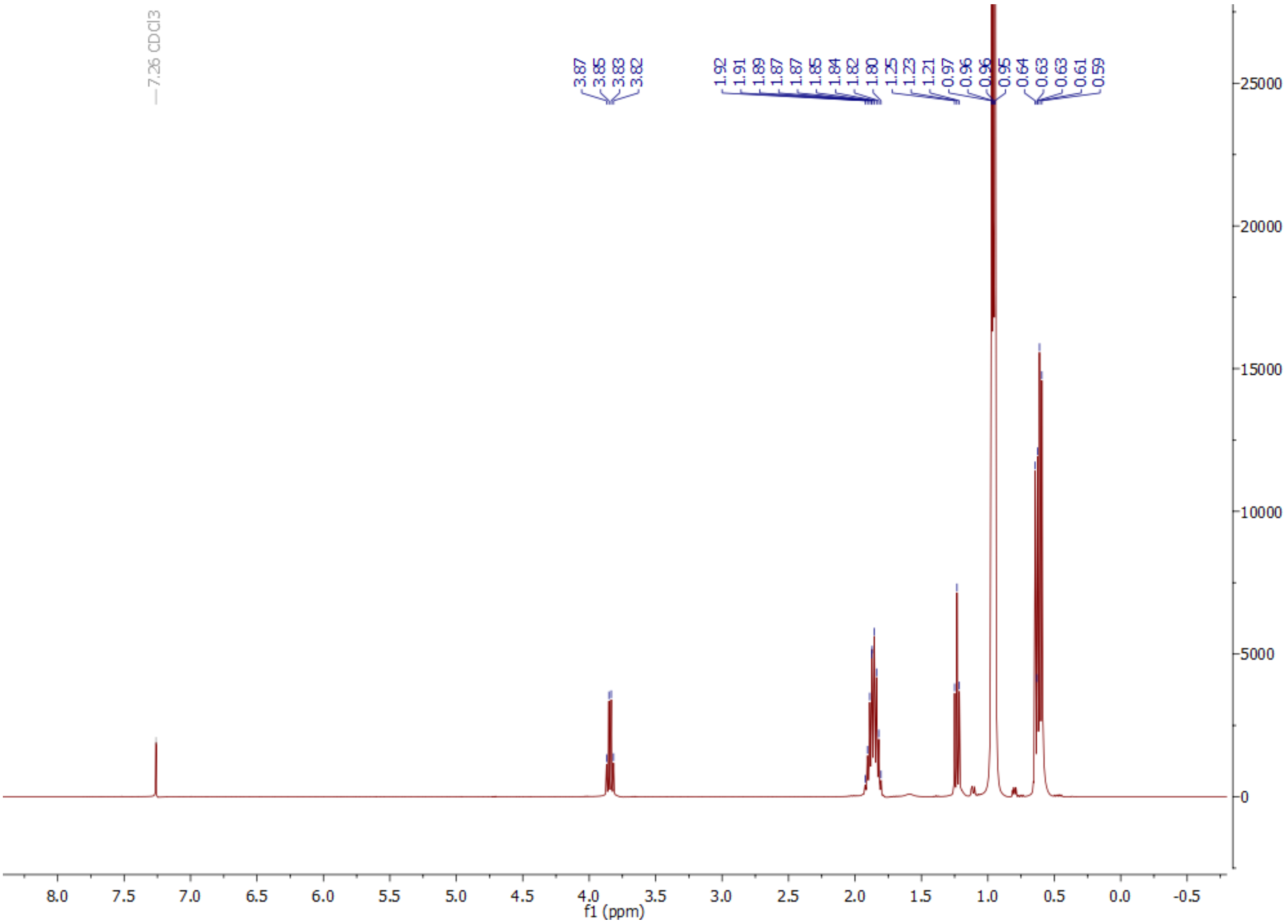

^{13}C NMR

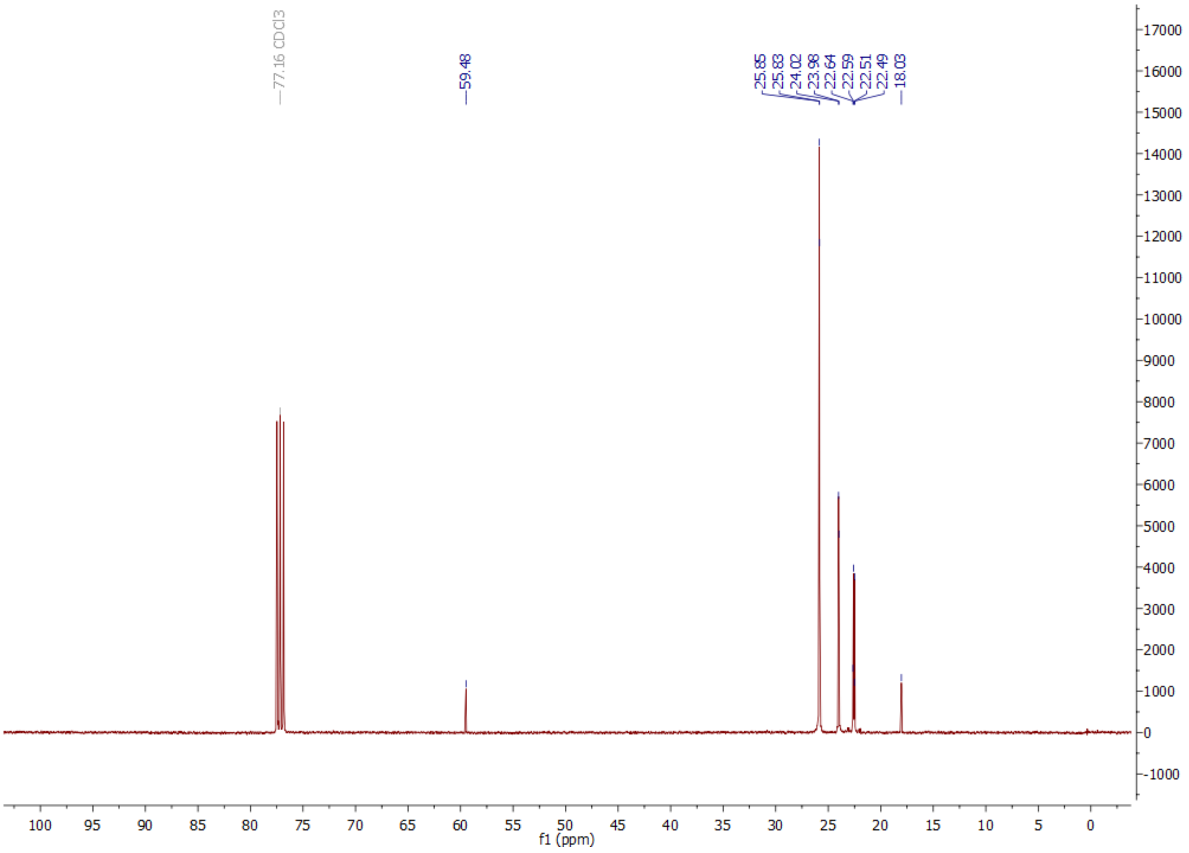

^{29}Si NMR

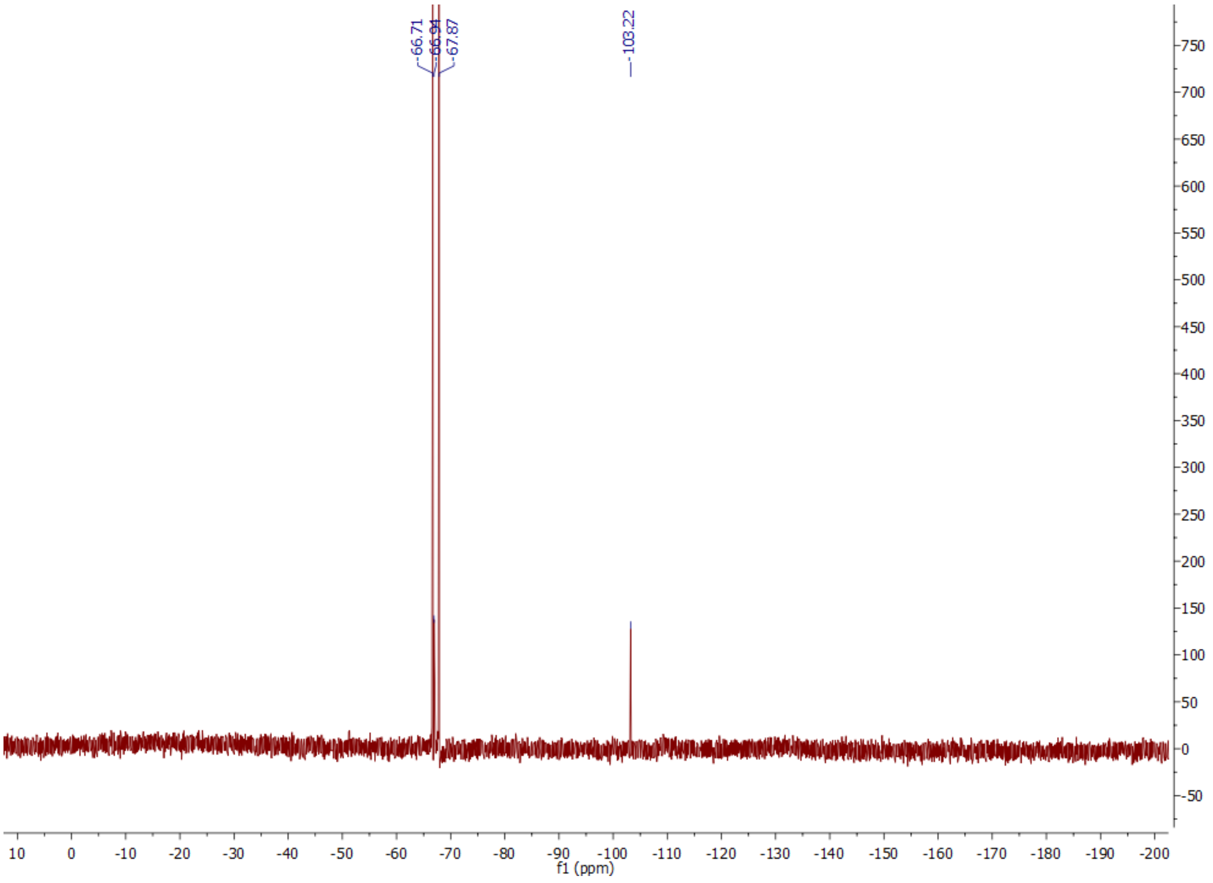

3. MALDI-TOF mass spectra of obtained spherosilicate compounds

SS-6GP-2TMOS

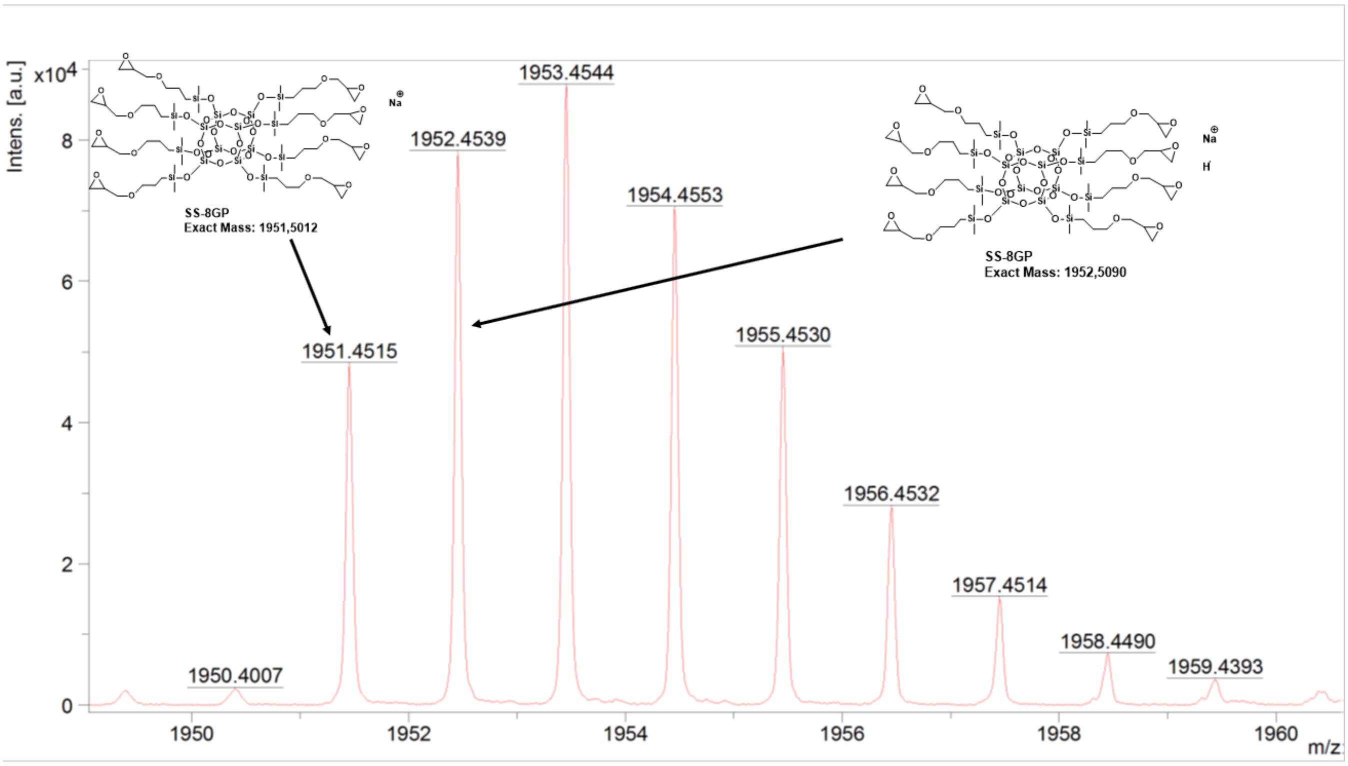

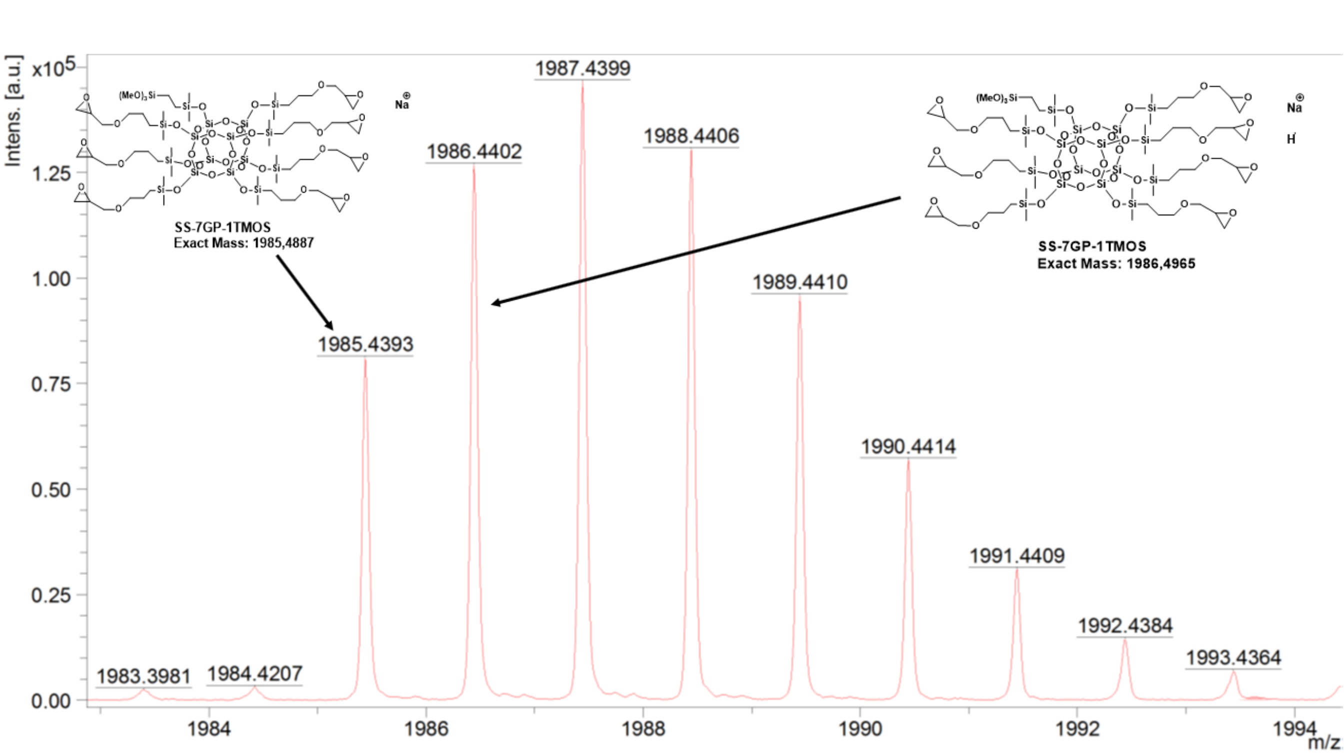

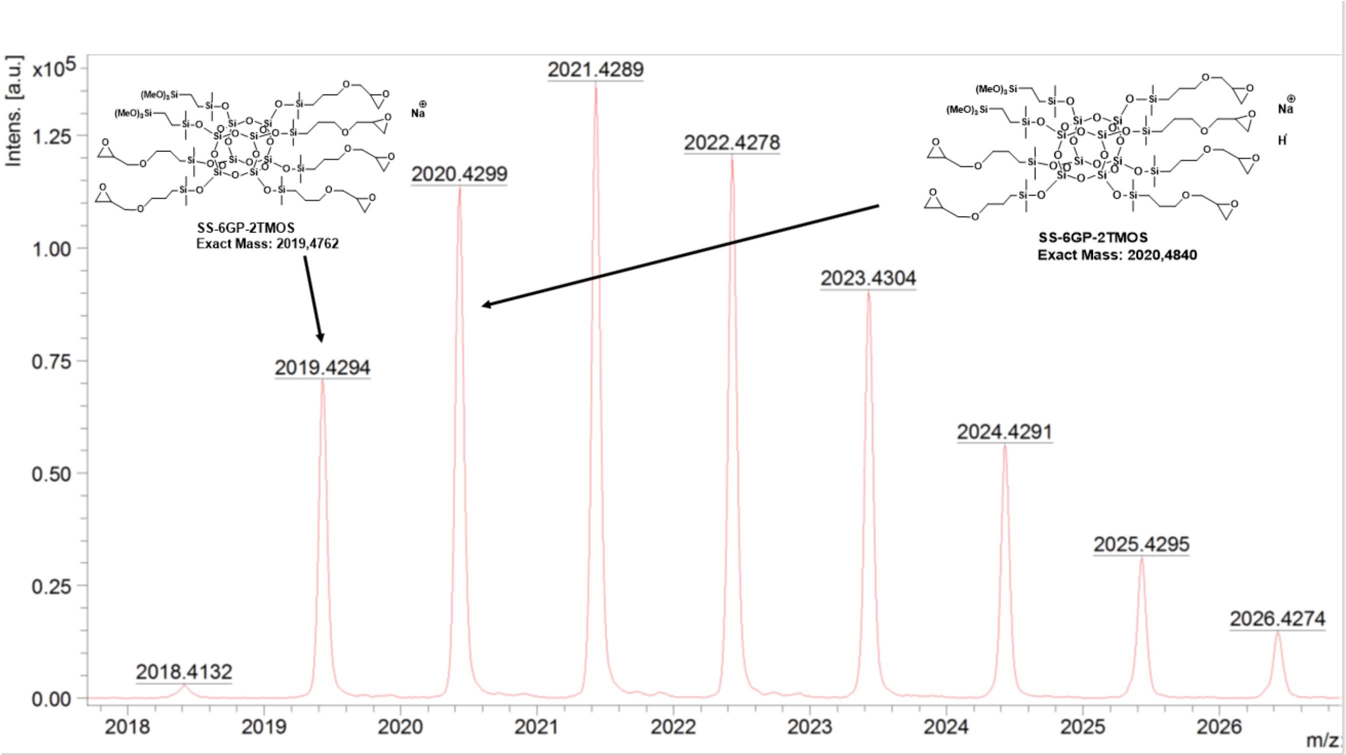

Intens. [a.u.]
x10^5
SS-6GP-2TMOS
Exact Mass: 2019,4762
SS-6GP-2TMOS
Exact Mass: 2020,4840
2018.4132
2019.4294
2020.4299
2021.4289
2022.4278
2023.4304
2024.4291
2025.4295
2026.4274
m/z

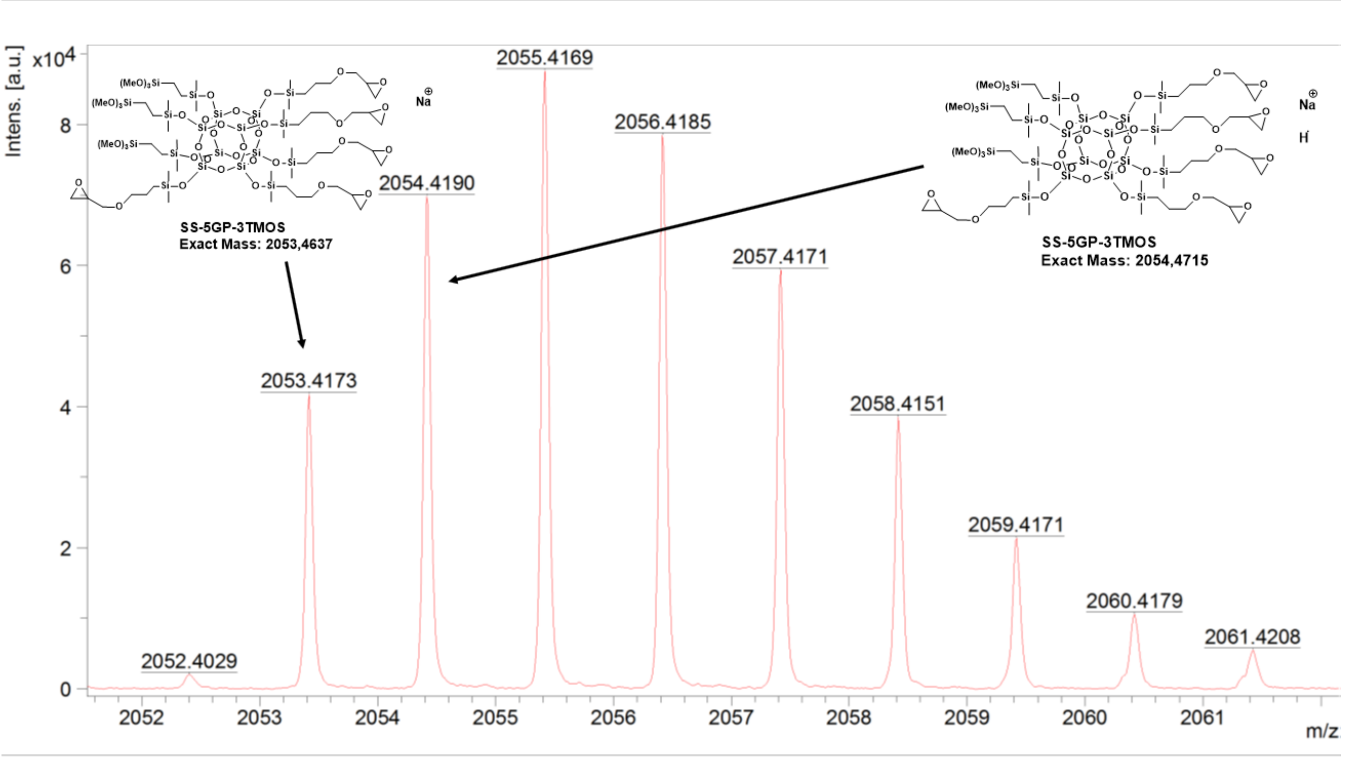

Intens. [a.u.]
x10^4
SS-5GP-3TMOS
Exact Mass: 2053,4637
SS-5GP-3TMOS
Exact Mass: 2054,4715
2052.4029
2053.4173
2054.4190
2055.4169
2056.4185
2057.4171
2058.4151
2059.4171
2060.4179
2061.4208
m/z

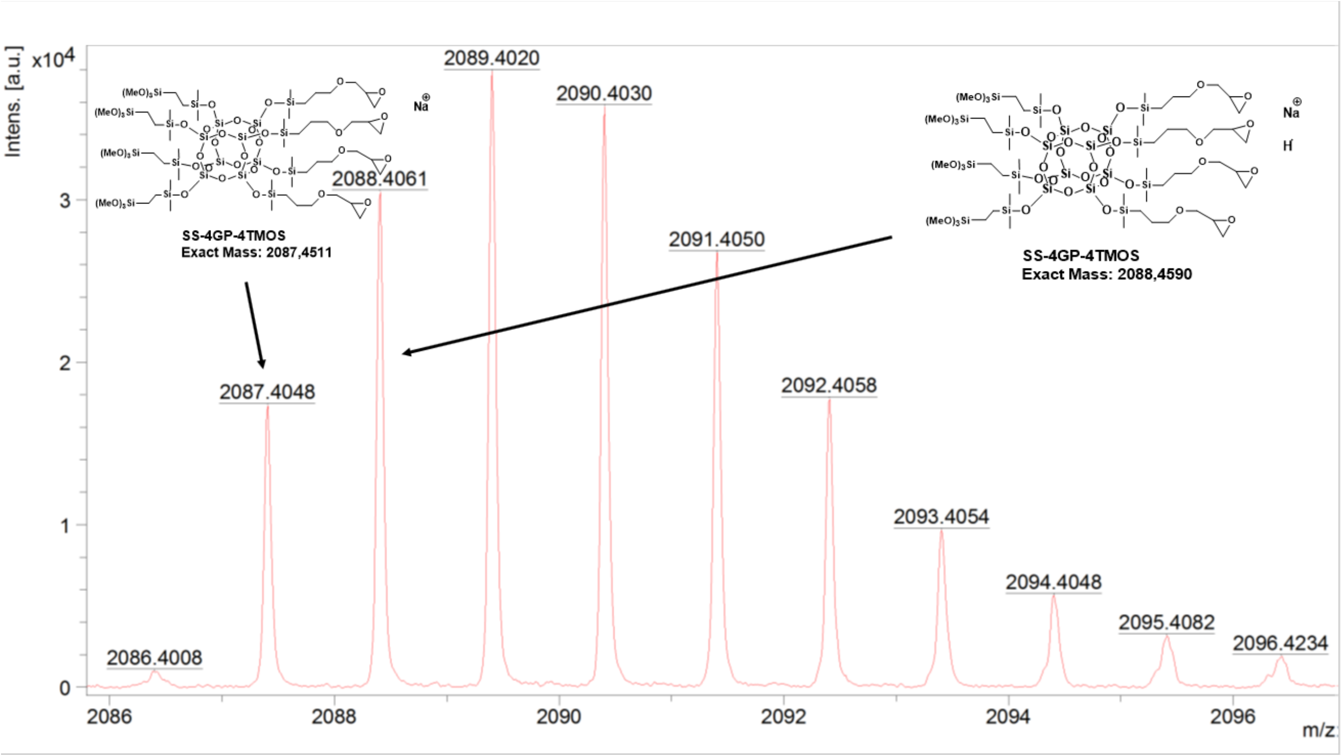
Intens. [a.u.]
x10^4
SS-4GP-4TMOS
Exact Mass: 2087,4511
SS-4GP-4TMOS
Exact Mass: 2088,4590
2086.4008
2087.4048
2088.4061
2089.4020
2090.4030
2091.4050
2092.4058
2093.4054
2094.4048
2095.4082
2096.4234
m/z

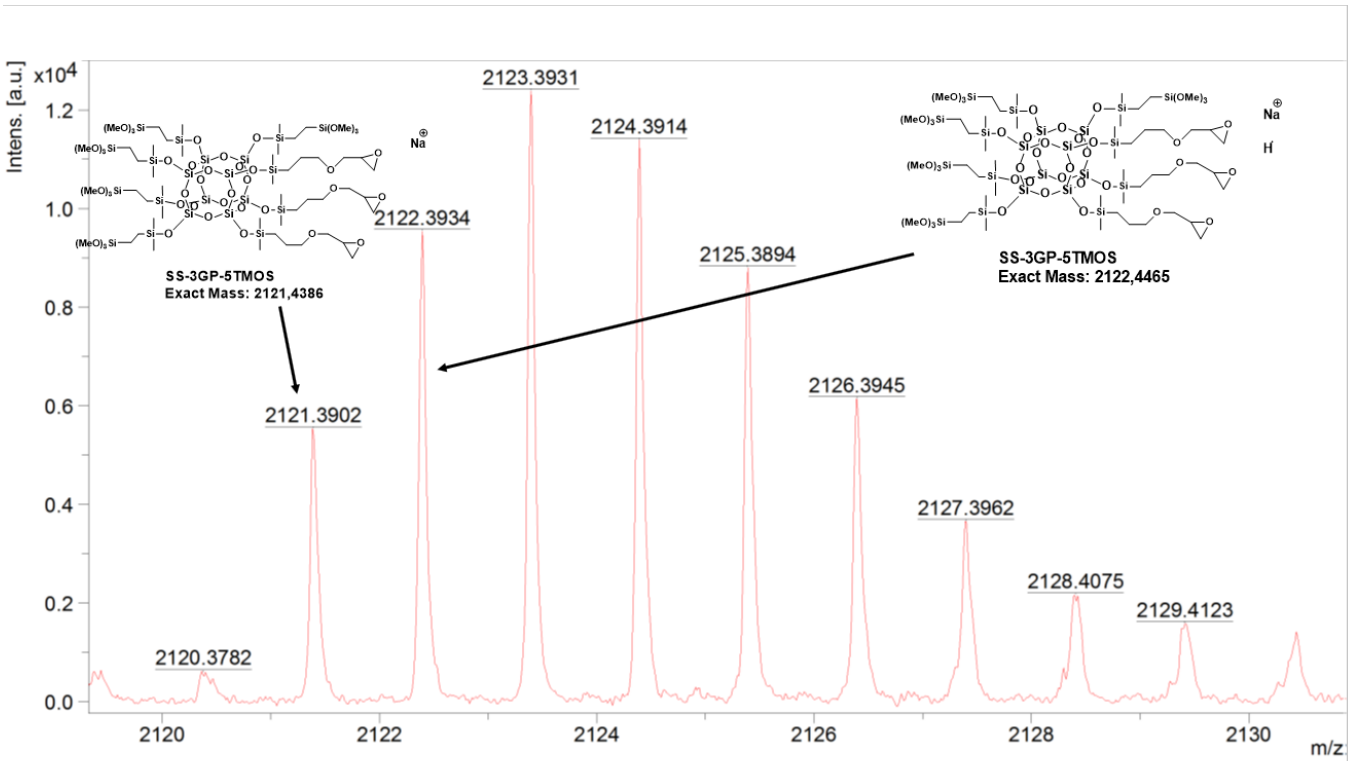
Intens. [a.u.]
x10^4
SS-3GP-5TMOS
Exact Mass: 2121,4386
SS-3GP-5TMOS
Exact Mass: 2122,4465
2120.3782
2121.3902
2122.3934
2123.3931
2124.3914
2125.3894
2126.3945
2127.3962
2128.4075
2129.4123
m/z

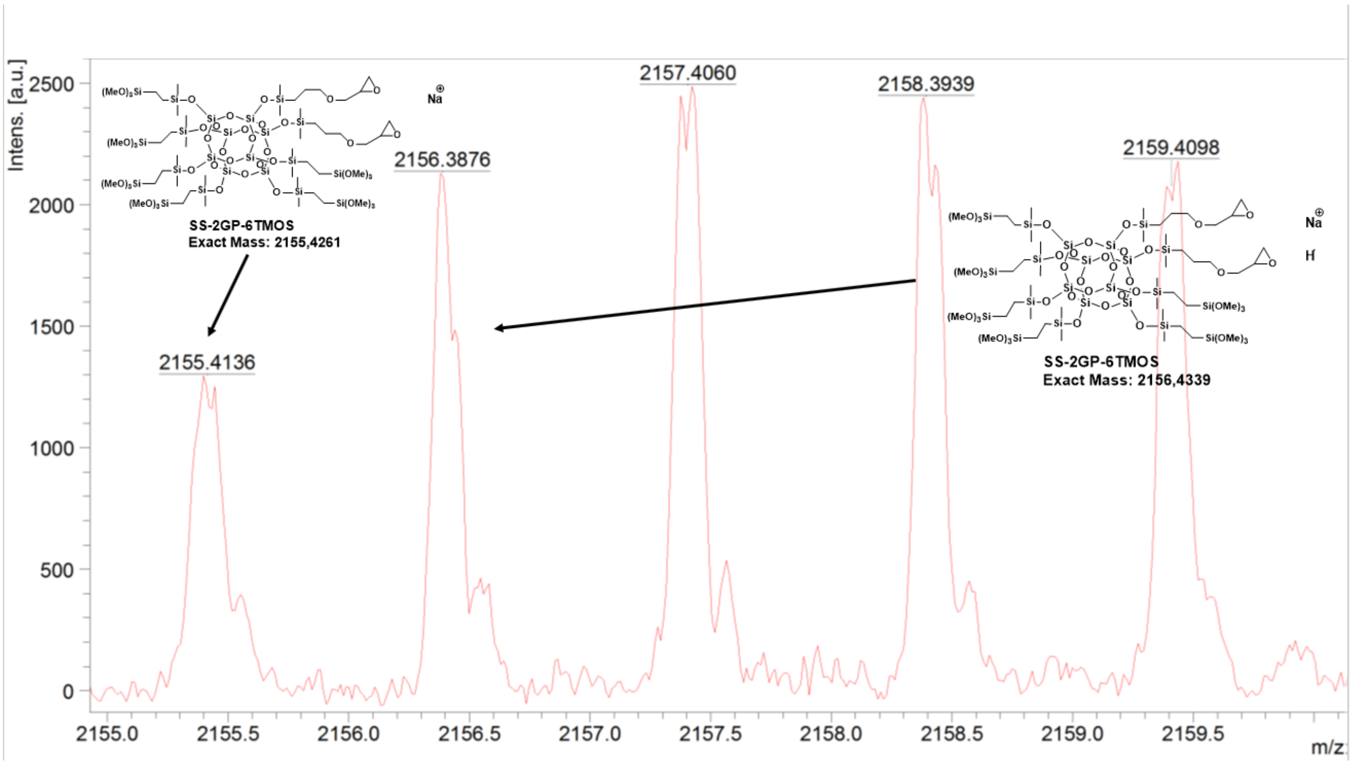
Intens. [a.u.]
2155.4136
2156.3876
2157.4060
2158.3939
2159.4098
SS-2GP-6TMOS
Exact Mass: 2155,4261
SS-2GP-6TMOS
Exact Mass: 2156,4339
m/z

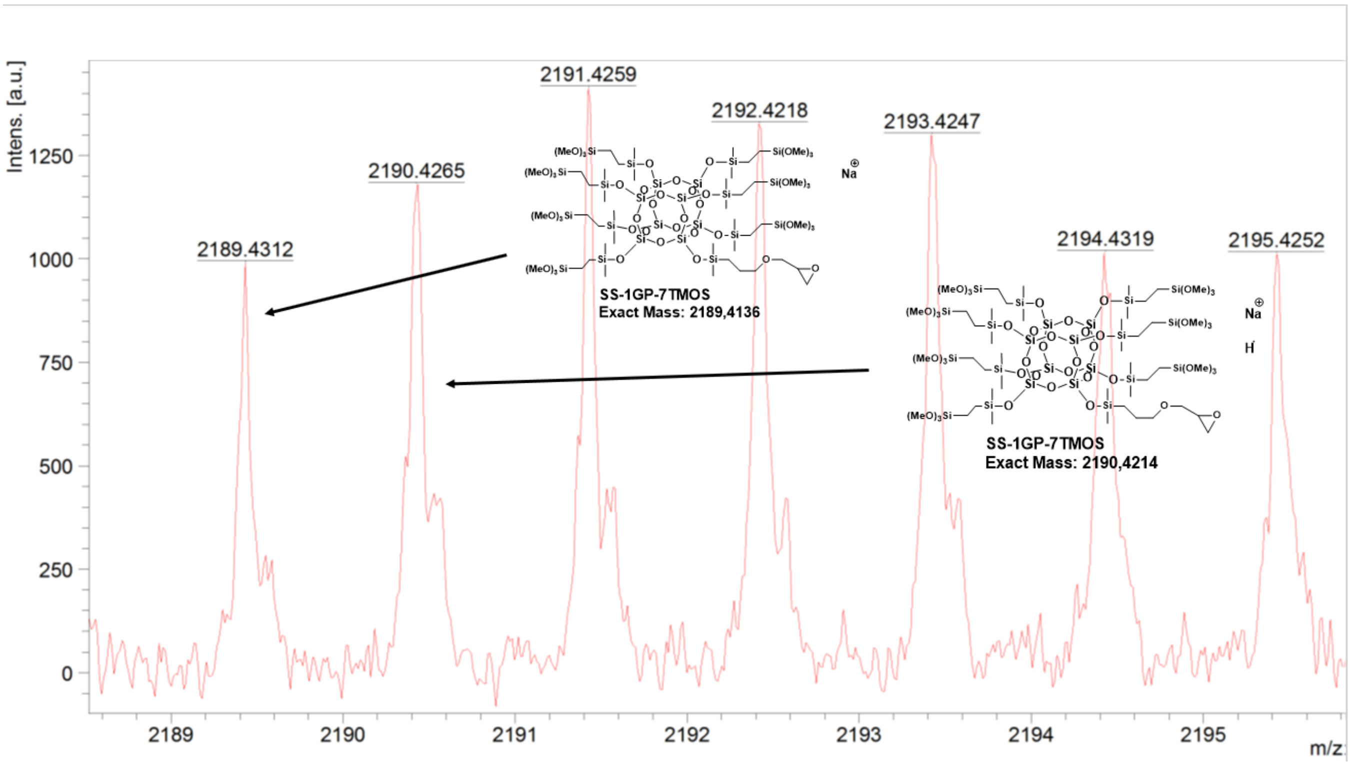
Intens. [a.u.]
2189.4312
2190.4265
2191.4259
2192.4218
2193.4247
2194.4319
2195.4252
SS-1GP-7TMOS
Exact Mass: 2189,4136
SS-1GP-7TMOS
Exact Mass: 2190,4214
m/z

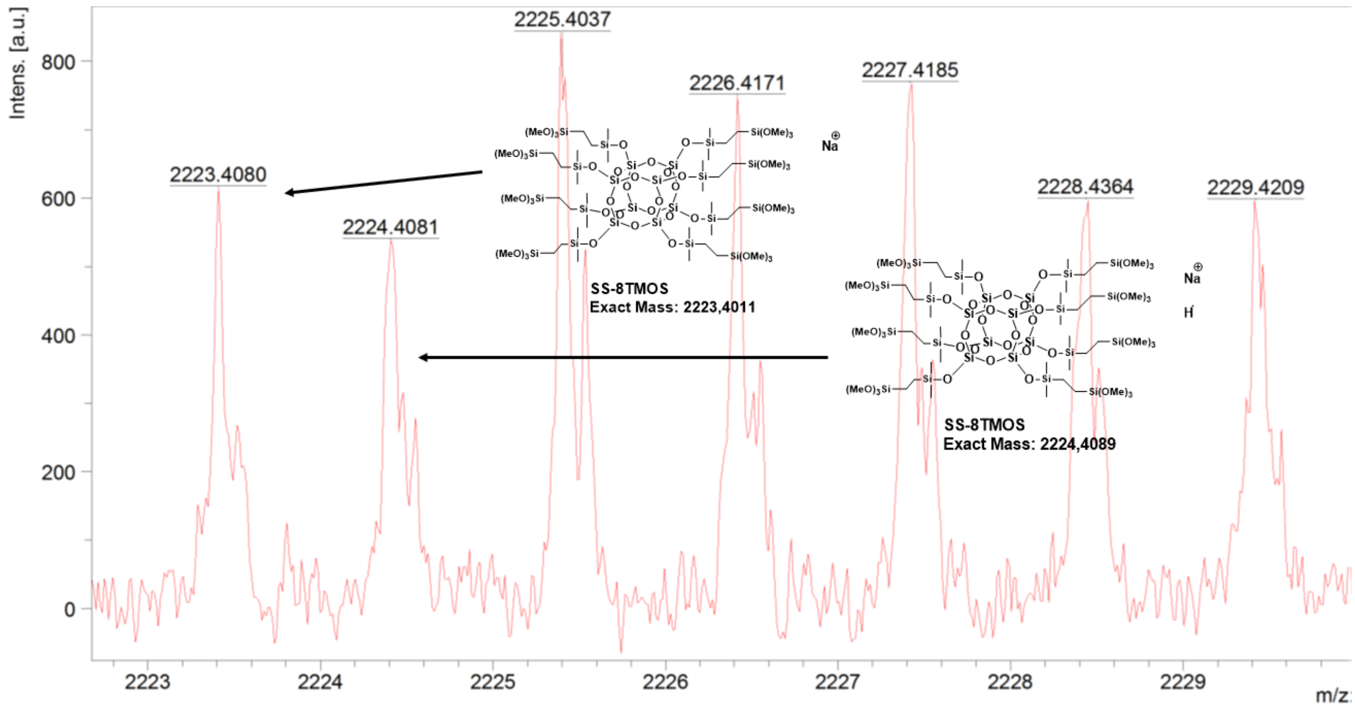
Intens. [a.u.]
2223.4080
2224.4081
2225.4037
2226.4171
2227.4185
2228.4364
2229.4209
SS-8TMOS
Exact Mass: 2223,4011
SS-8TMOS
Exact Mass: 2224,4089
m/z

SS-5GP-3TMOS

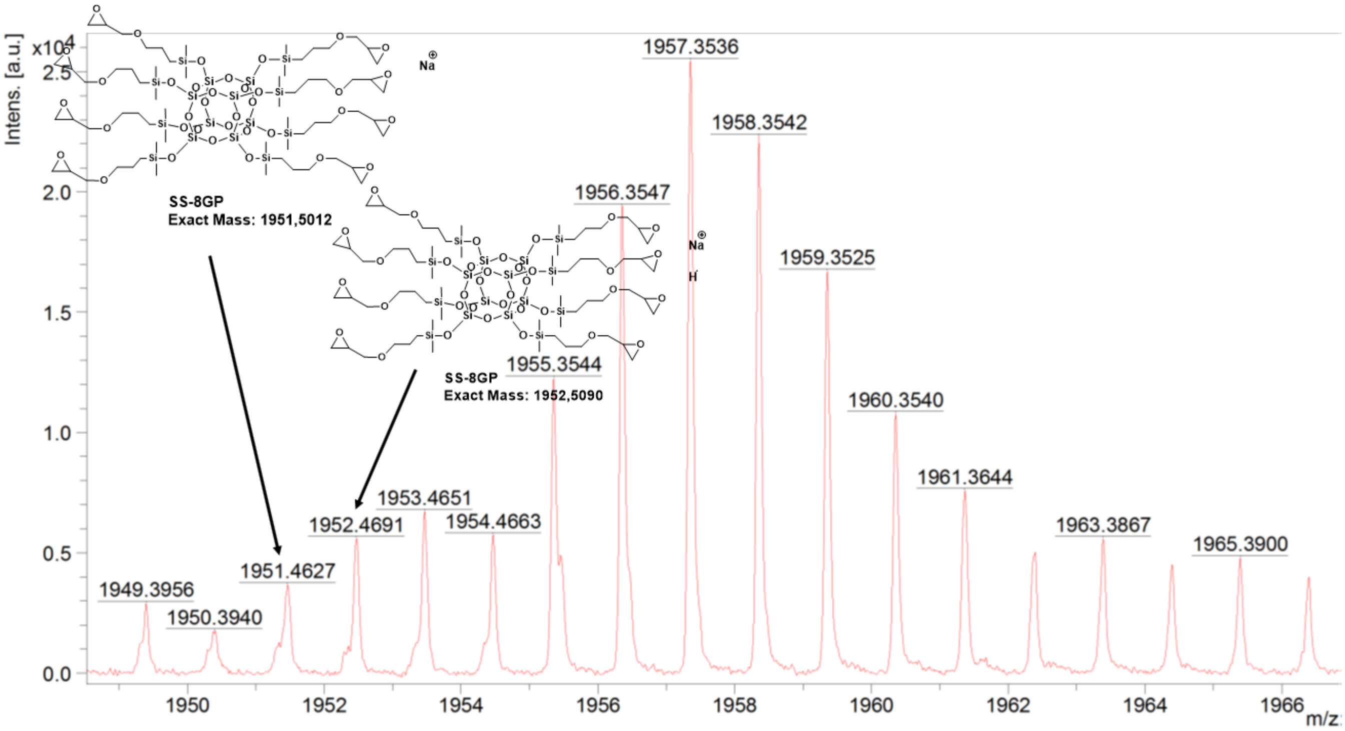

x10^4
Intens. [a.u.]
SS-8GP
Exact Mass: 1951,5012
SS-8GP
Exact Mass: 1952,5090
1949.3956
1950.3940
1951.4627
1952.4691
1953.4651
1954.4663
1955.3544
1956.3547
1957.3536
1958.3542
1959.3525
1960.3540
1961.3644
1963.3867
1965.3900
m/z

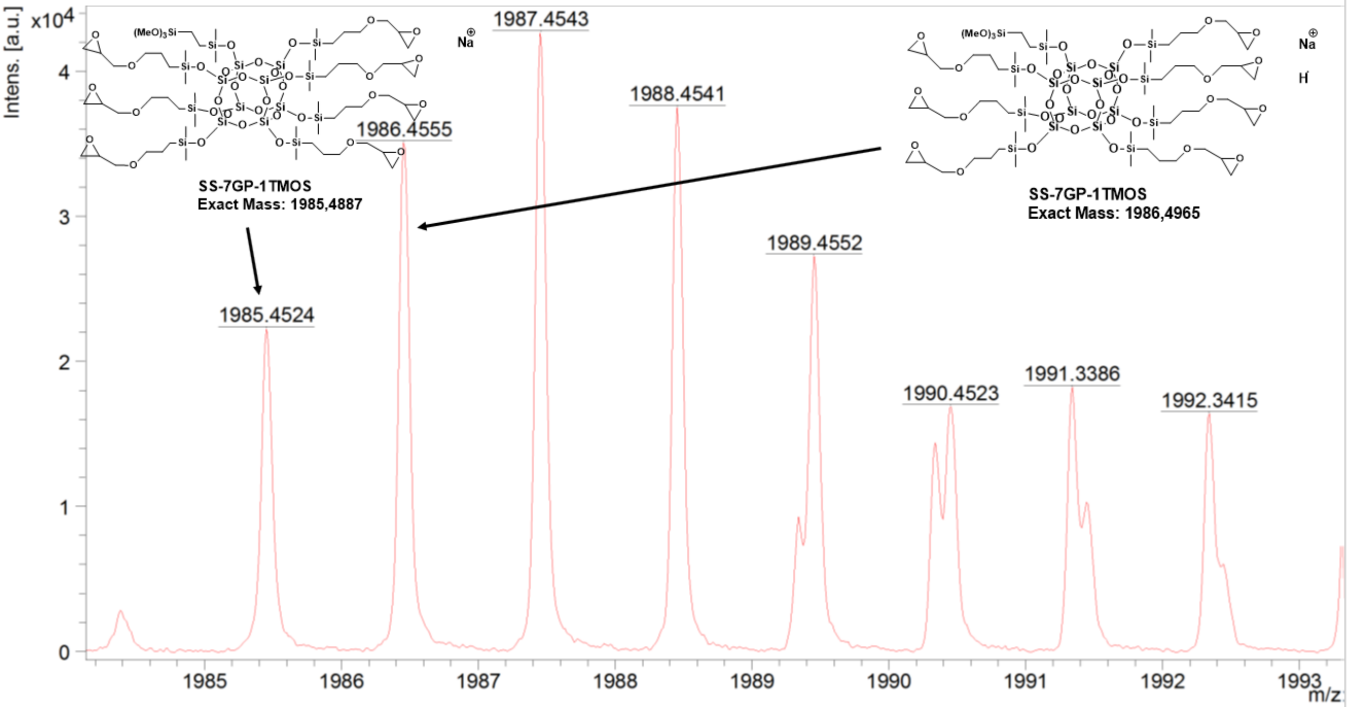

x10^4
Intens. [a.u.]
SS-7GP-1TMOS
Exact Mass: 1985,4887
SS-7GP-1TMOS
Exact Mass: 1986,4965
1985.4524
1986.4555
1987.4543
1988.4541
1989.4552
1990.4523
1991.3386
1992.3415
m/z

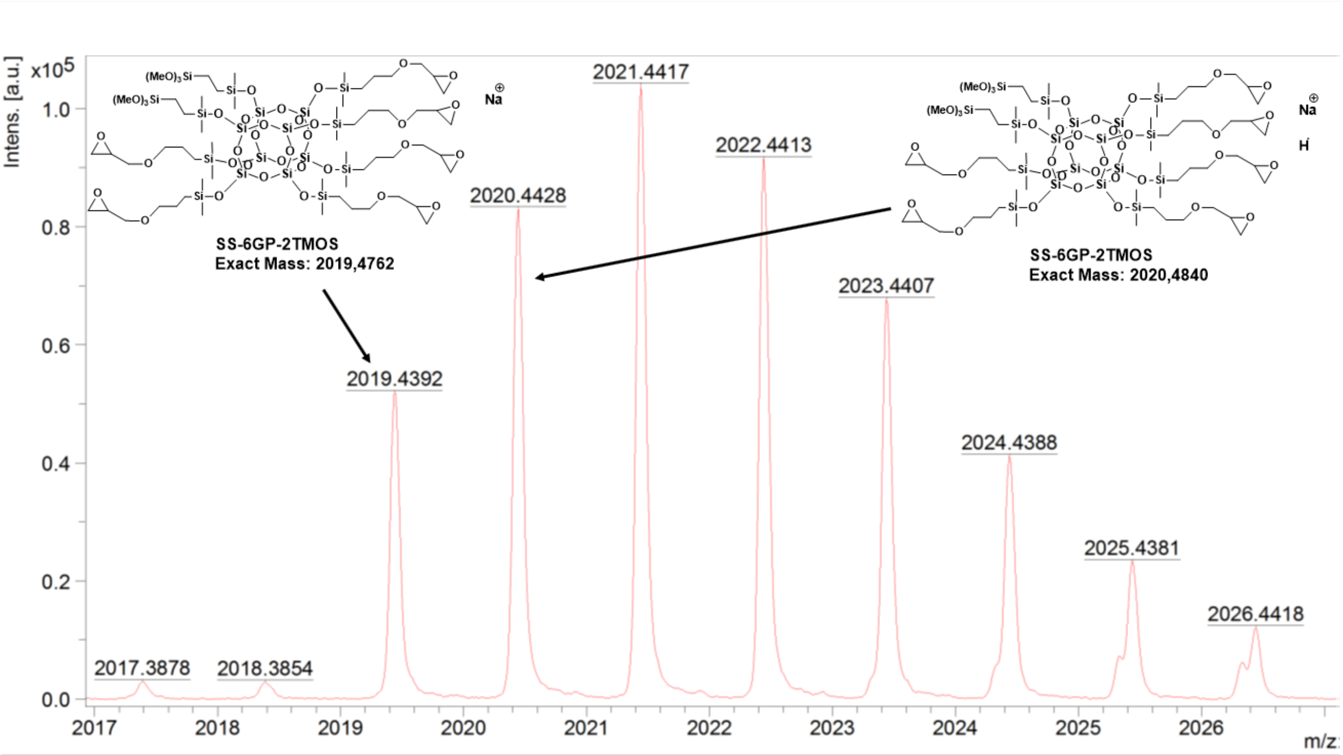
Intens. [a.u.]
x10^5
SS-6GP-2TMOS
Exact Mass: 2019,4762
SS-6GP-2TMOS
Exact Mass: 2020,4840
2017.3878
2018.3854
2019.4392
2020.4428
2021.4417
2022.4413
2023.4407
2024.4388
2025.4381
2026.4418
m/z

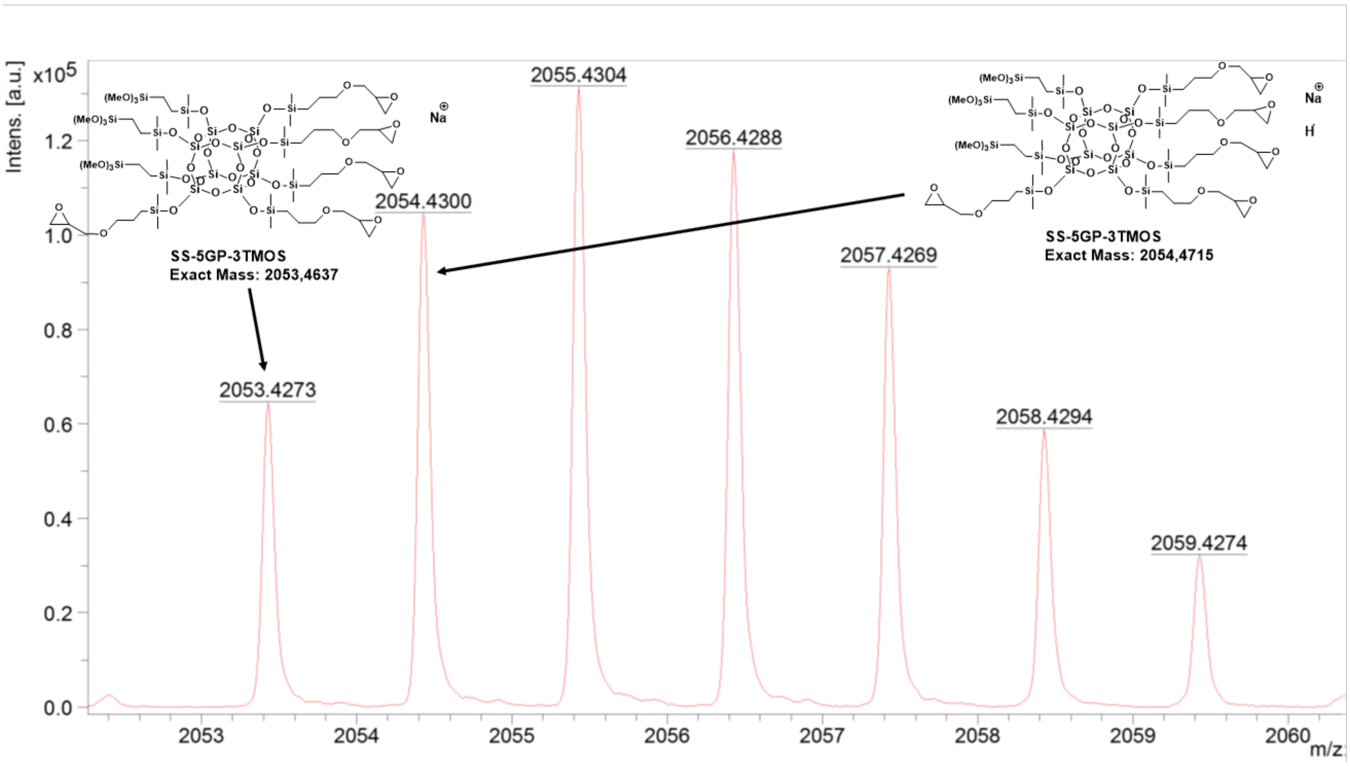
Intens. [a.u.]
x10^5
SS-5GP-3TMOS
Exact Mass: 2053,4637
SS-5GP-3TMOS
Exact Mass: 2054,4715
2053.4273
2054.4300
2055.4304
2056.4288
2057.4269
2058.4294
2059.4274
m/z

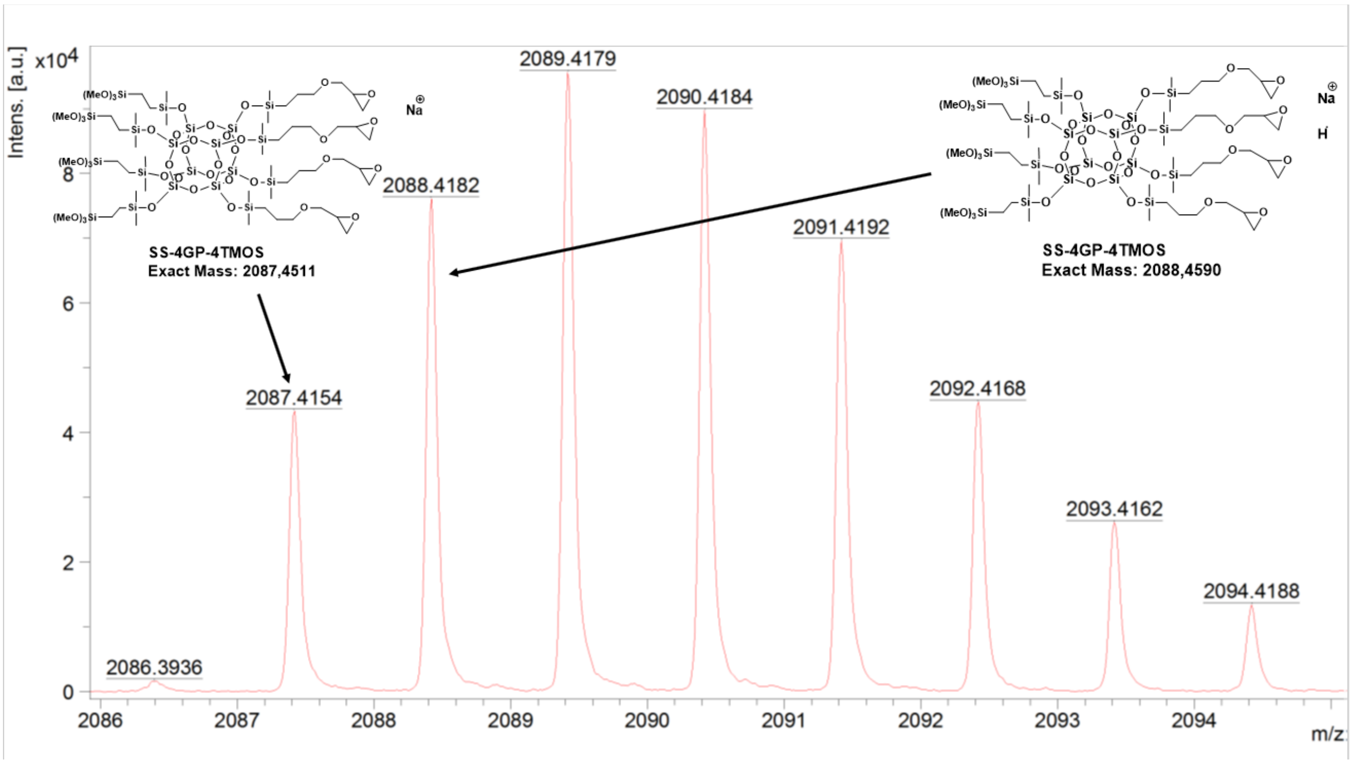
x10^4
Intens. [a.u.]
SS-4GP-4TMOS
Exact Mass: 2087,4511
SS-4GP-4TMOS
Exact Mass: 2088,4590
2086.3936
2087.4154
2088.4182
2089.4179
2090.4184
2091.4192
2092.4168
2093.4162
2094.4188
m/z

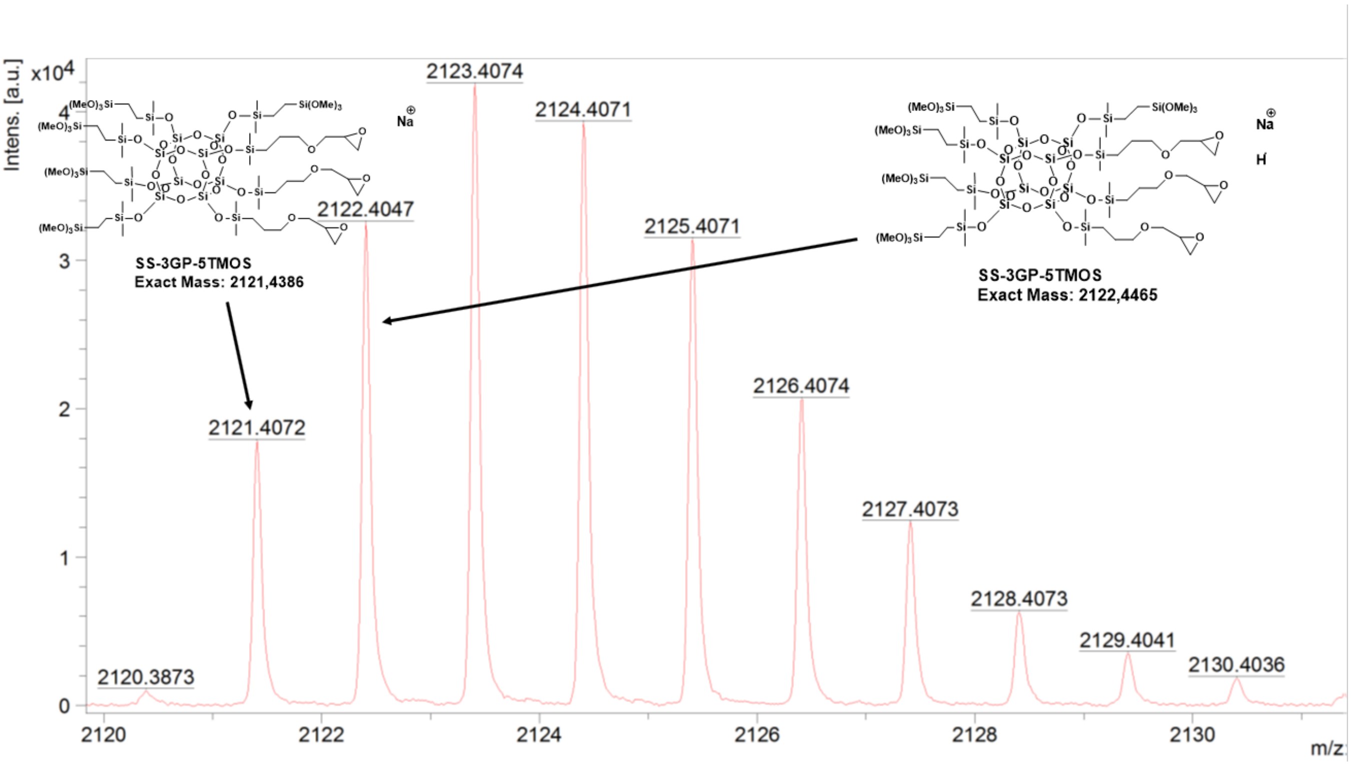
x10^4
Intens. [a.u.]
SS-3GP-5TMOS
Exact Mass: 2121,4386
SS-3GP-5TMOS
Exact Mass: 2122,4465
2120.3873
2121.4072
2122.4047
2123.4074
2124.4071
2125.4071
2126.4074
2127.4073
2128.4073
2129.4041
2130.4036
m/z

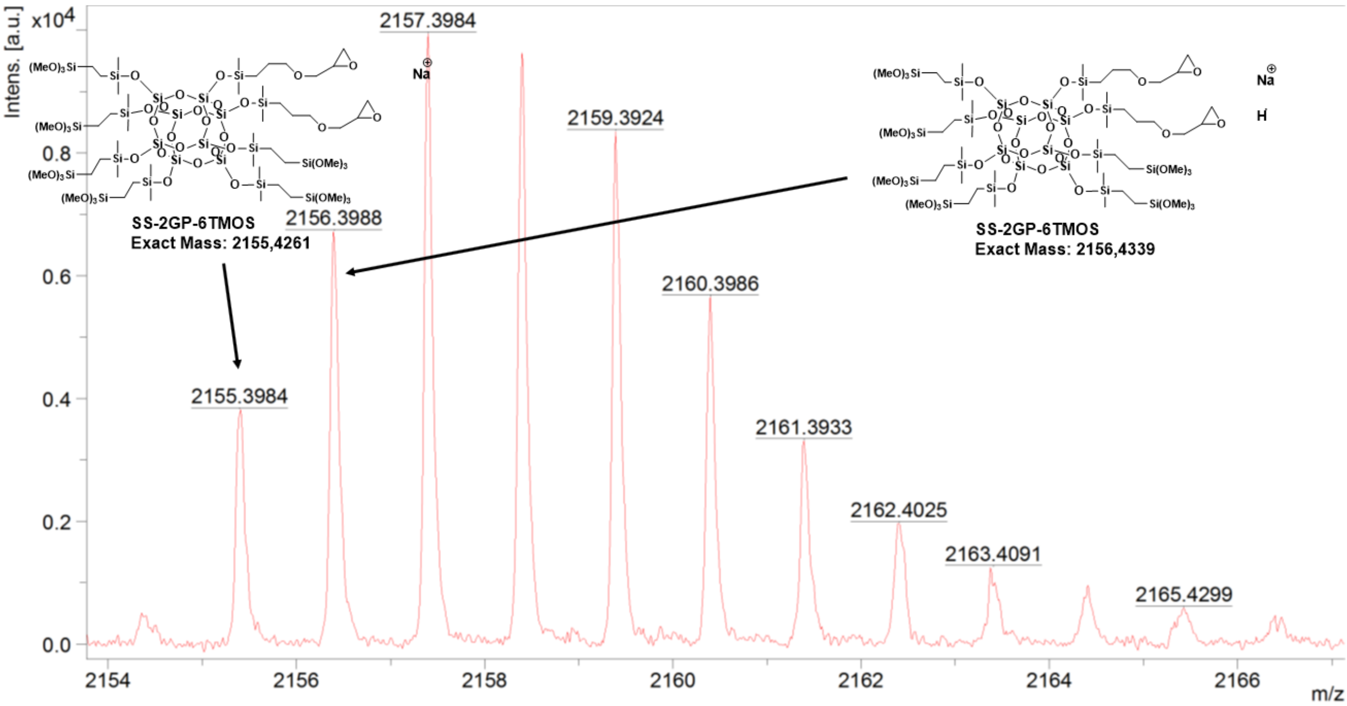

Intens. [a.u.]
x10^4
SS-2GP-6TMOS
Exact Mass: 2155,4261
SS-2GP-6TMOS
Exact Mass: 2156,4339
2155.3984
2156.3988
2157.3984
2159.3924
2160.3986
2161.3933
2162.4025
2163.4091
2165.4299
m/z

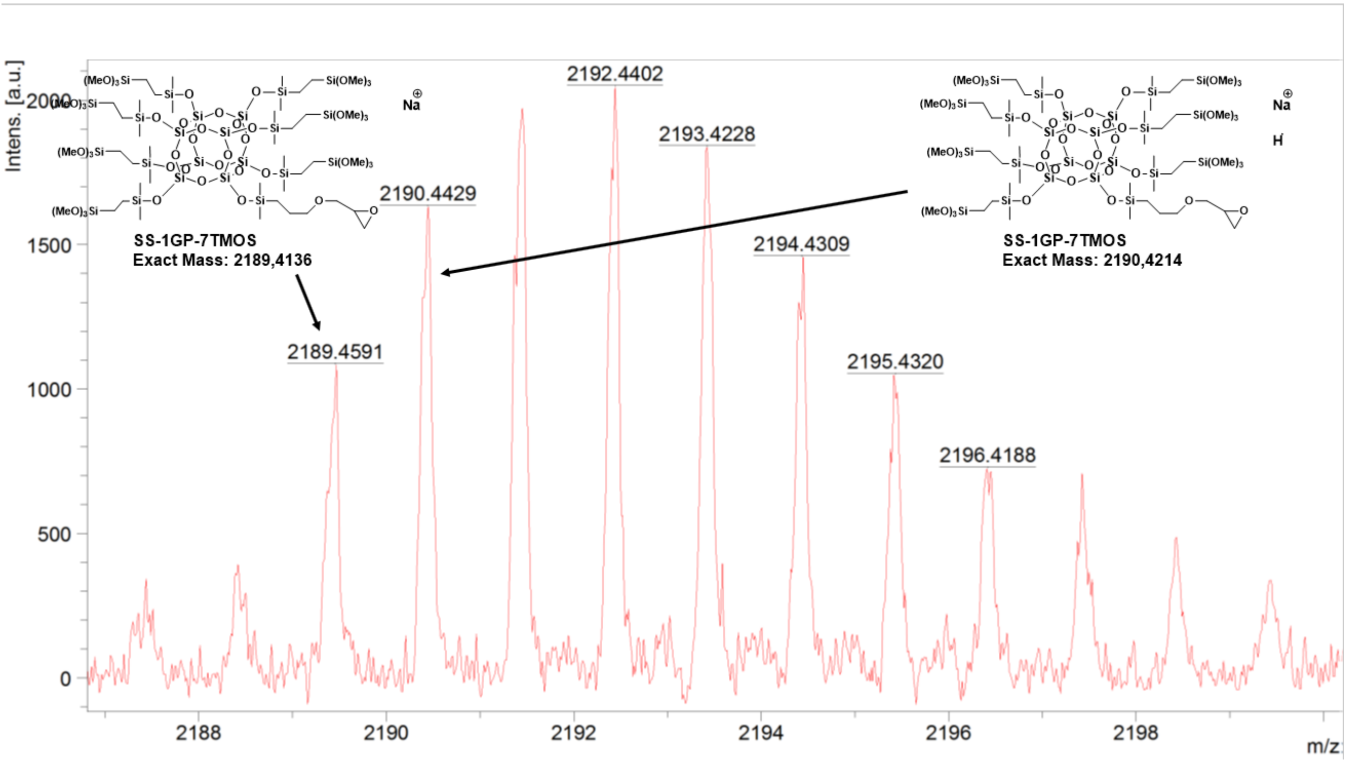

Intens. [a.u.]
SS-1GP-7TMOS
Exact Mass: 2189,4136
SS-1GP-7TMOS
Exact Mass: 2190,4214
2189.4591
2190.4429
2192.4402
2193.4228
2194.4309
2195.4320
2196.4188
m/z

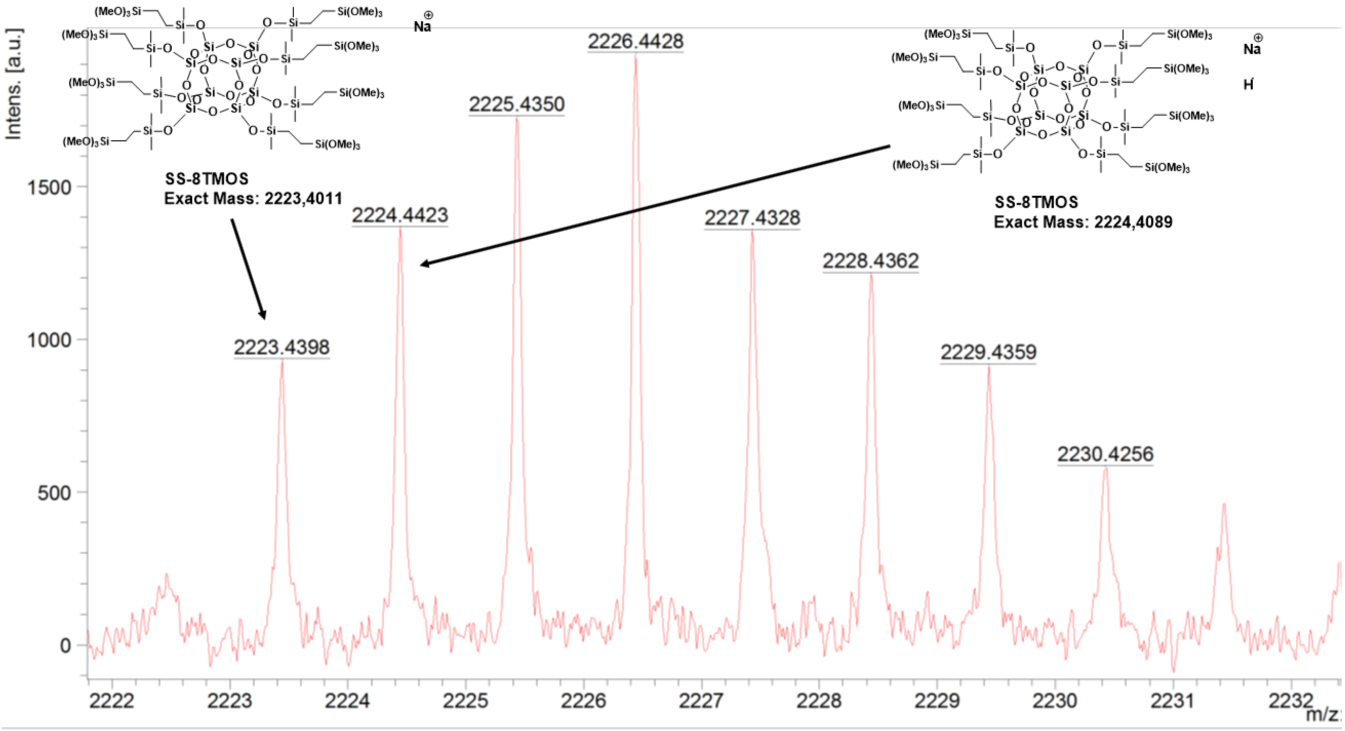
Intens. [a.u.]
1500
1000
500
0
SS-8TMOS
Exact Mass: 2223,4011
SS-8TMOS
Exact Mass: 2224,4089
Na
H
(MeO)3 Si
Si(OMe)3
2223.4398
2224.4423
2225.4350
2226.4428
2227.4328
2228.4362
2229.4359
2230.4256
2222
2223
2224
2225
2226
2227
2228
2229
2230
2231
2232
m/z

4. SEM images of the TiO_2/EP composites

1% TiO_2, unmodified, mechanical stirrer

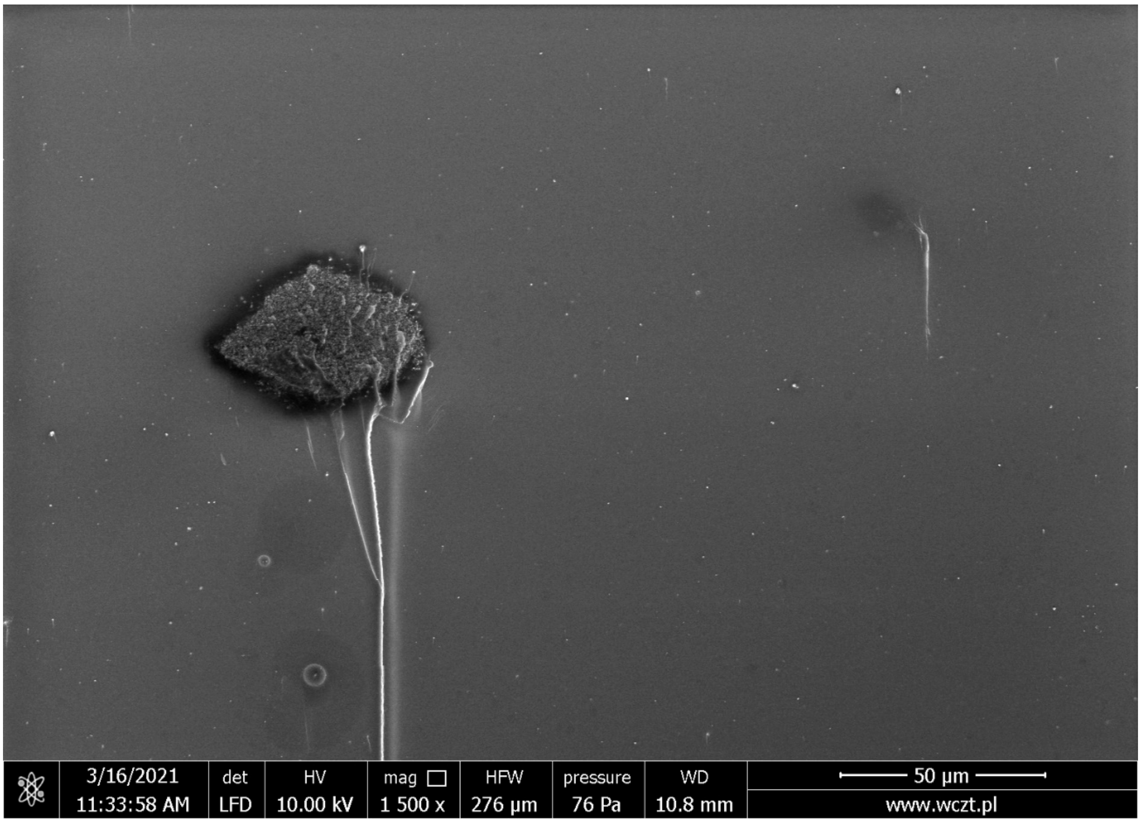

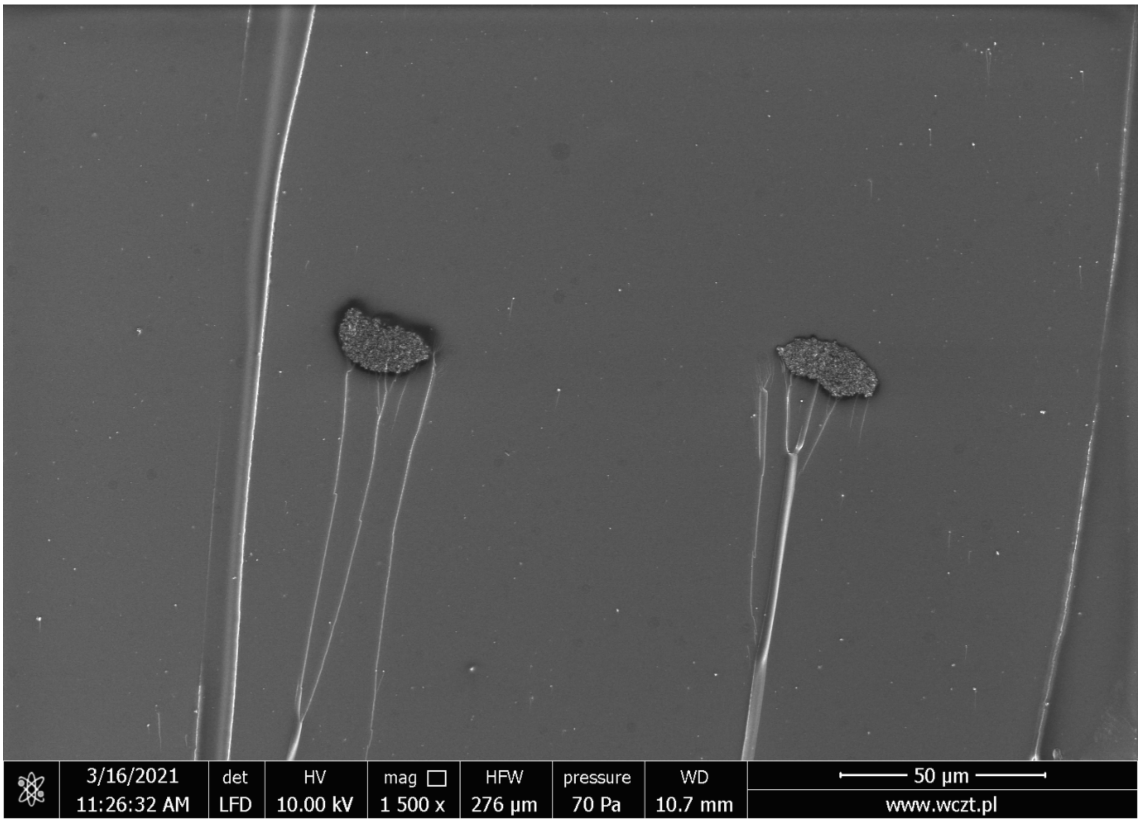

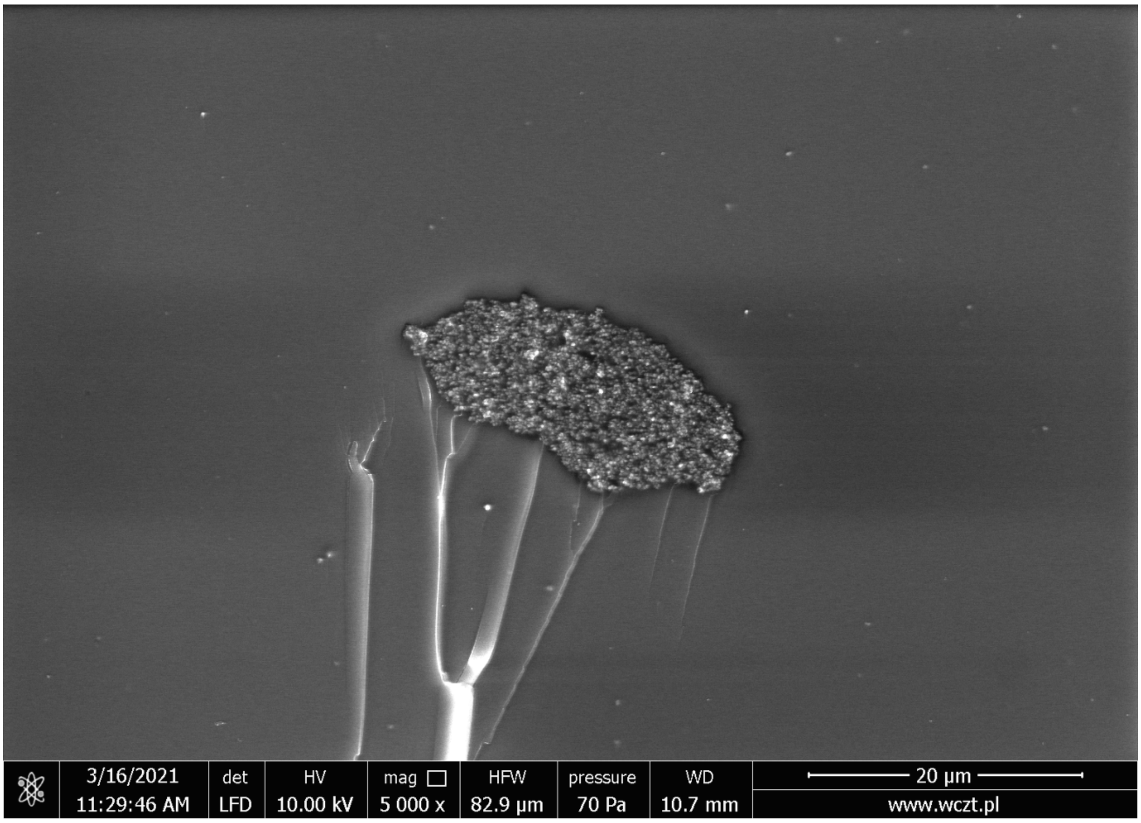
3/16/2021
11:29:46 AM
det
LFD
HV
10.00 kV
mag
5 000 x
HFW
82.9 µm
pressure
70 Pa
WD
10.7 mm
20 µm
www.wczt.pl

1% TiO_2, unmodified, mixing pump

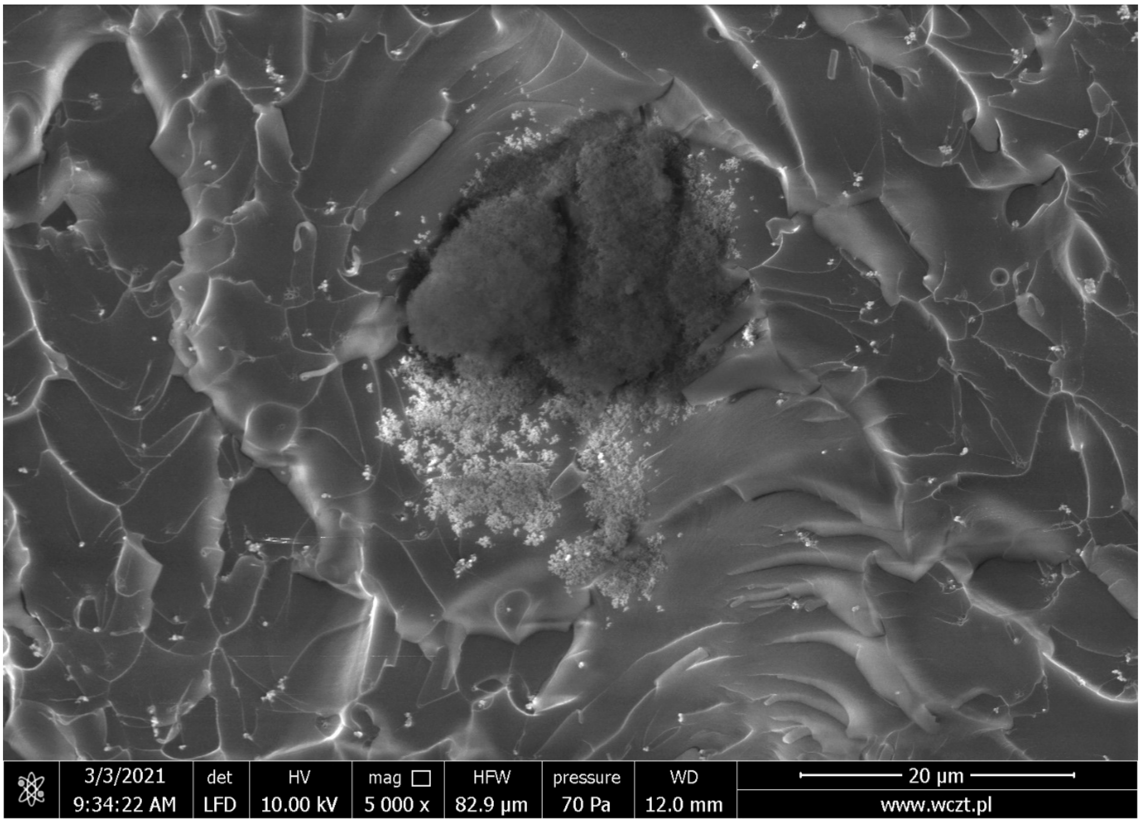

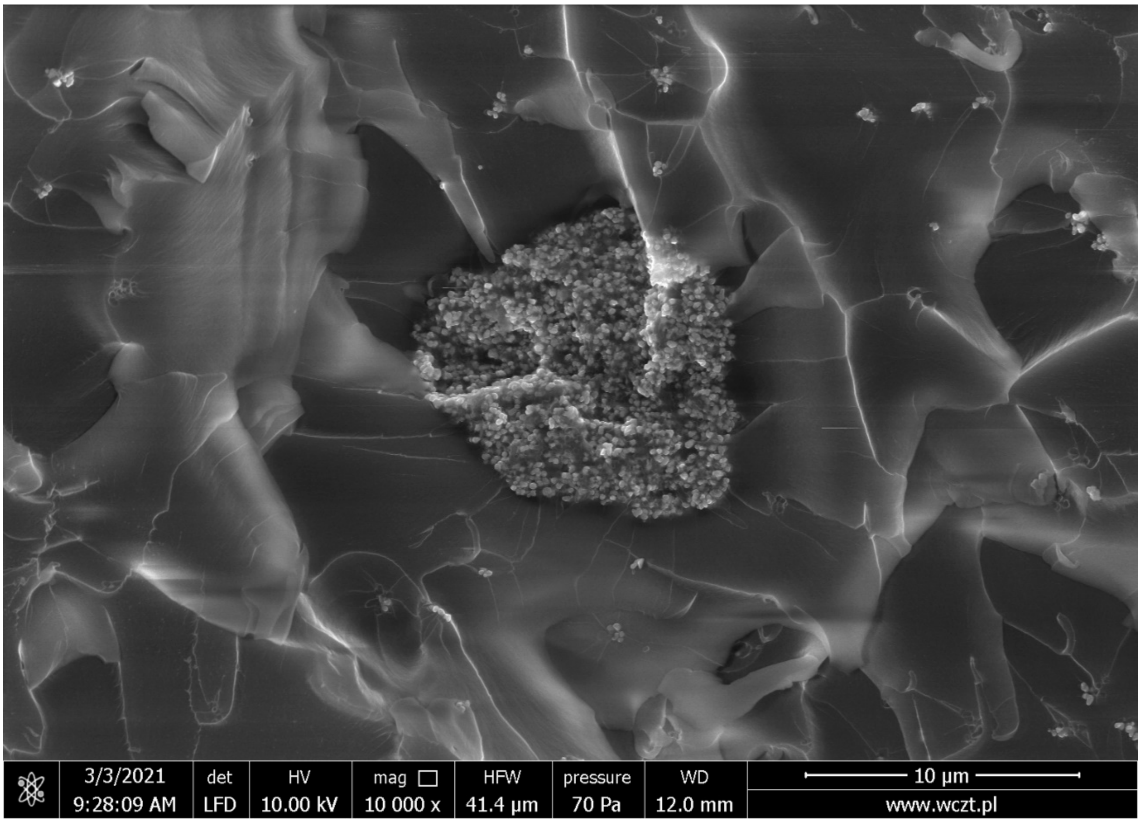

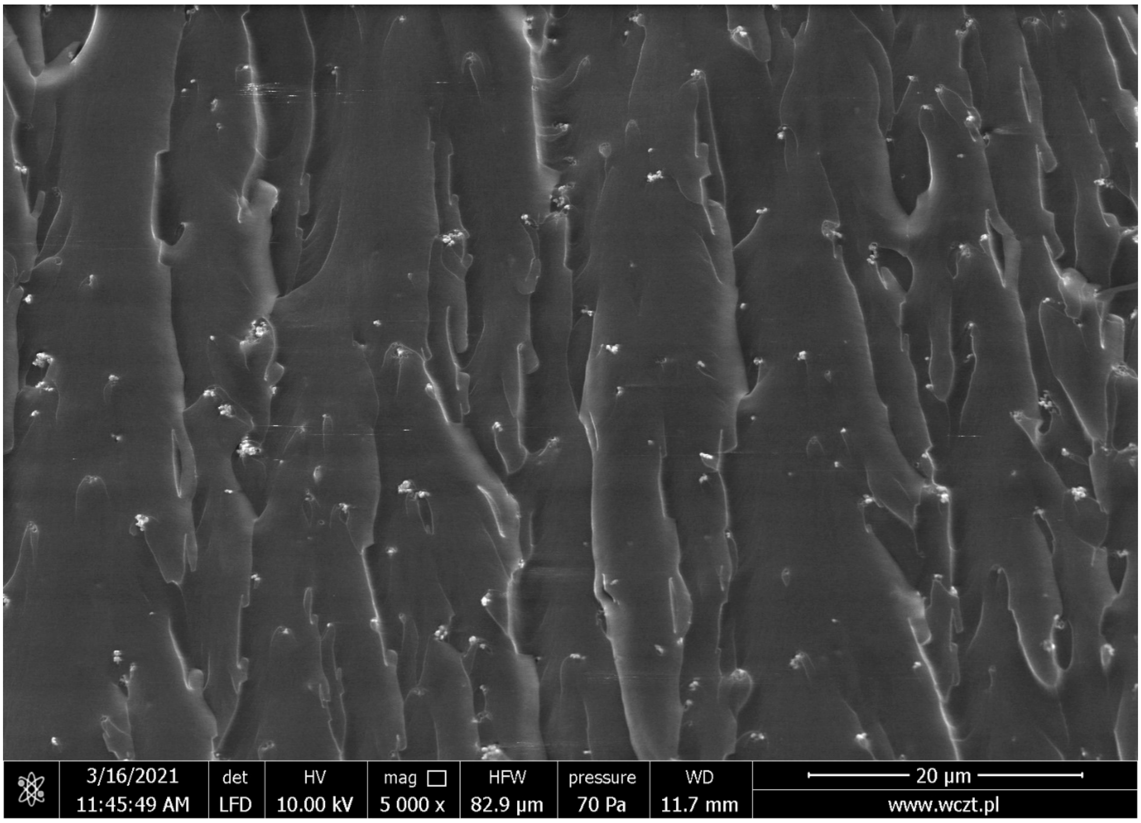
3/16/2021
11:45:49 AM
det
LFD
HV
10.00 kV
mag
5 000 x
HFW
82.9 µm
pressure
70 Pa
WD
11.7 mm
20 µm
www.wczt.pl

2% TiO_2, unmodified, mixing pump

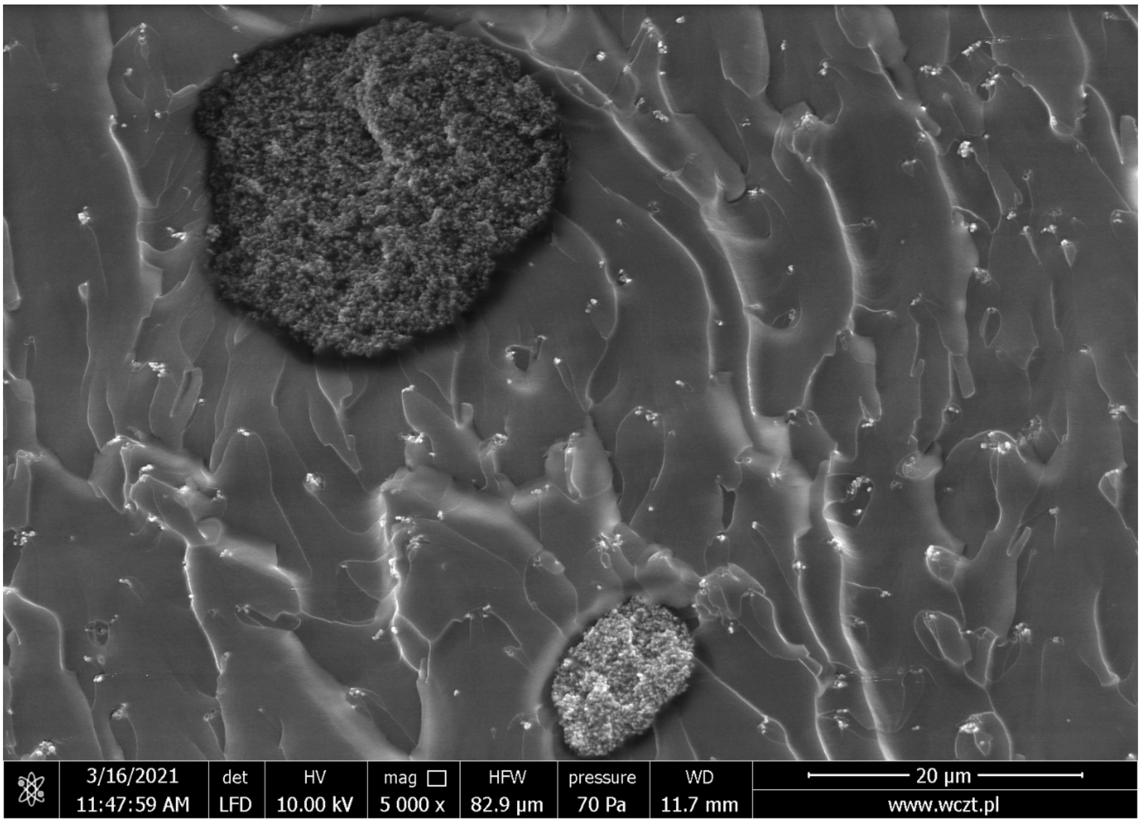

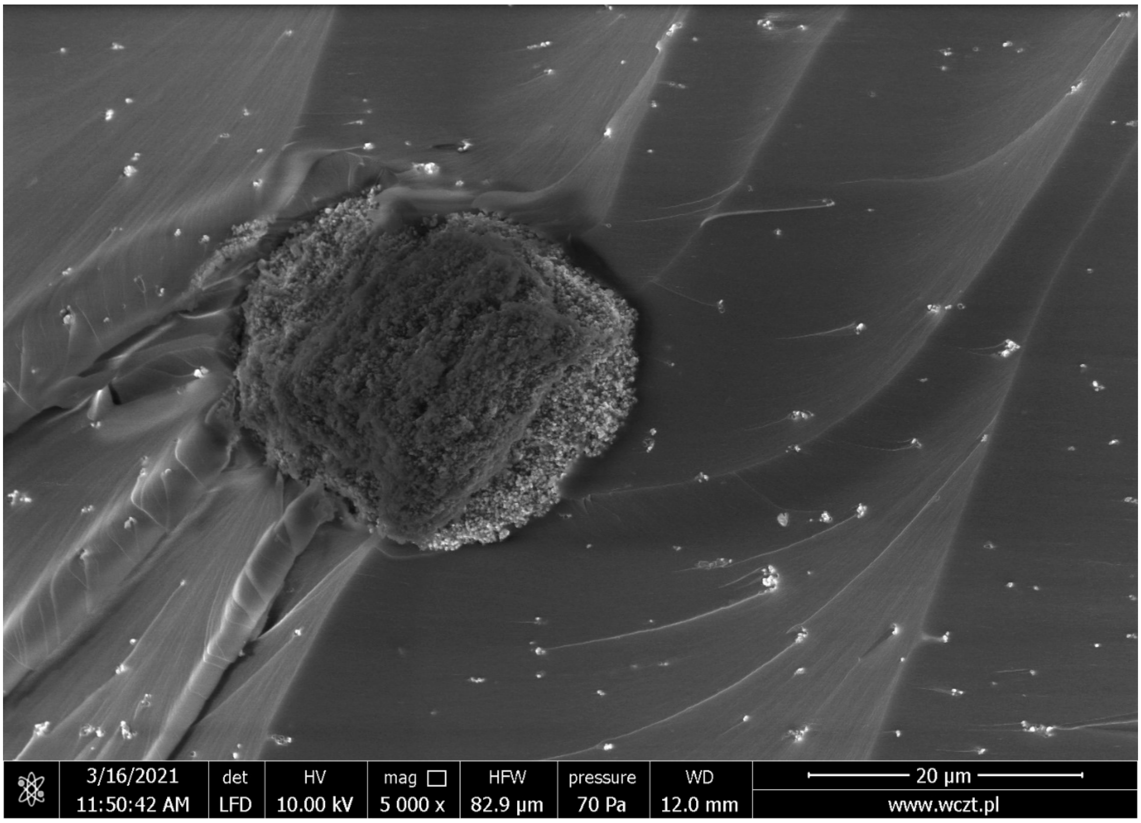

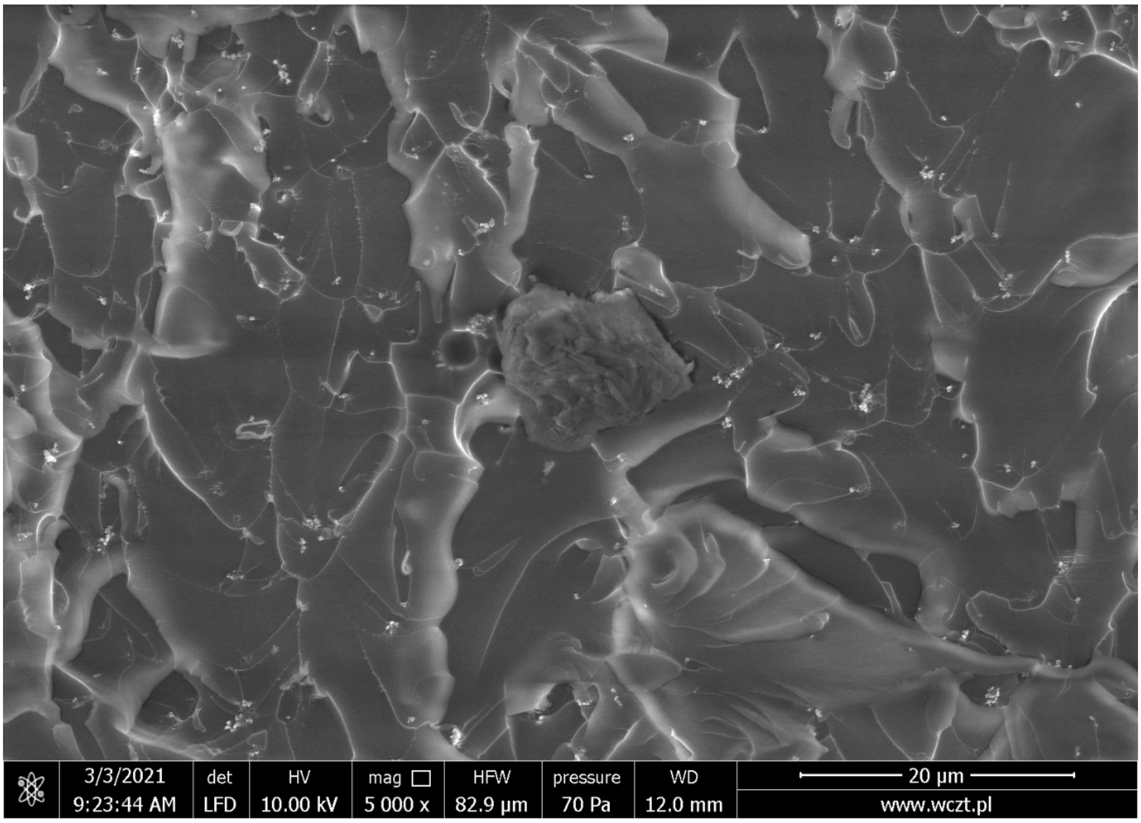
3/3/2021
9:23:44 AM
det
LFD
HV
10.00 kV
mag
5 000 x
HFW
82.9 μm
pressure
70 Pa
WD
12.0 mm
20 μm
www.wczt.pl

1%TiO₂, 0.5% iBuTMOS, mixing pump

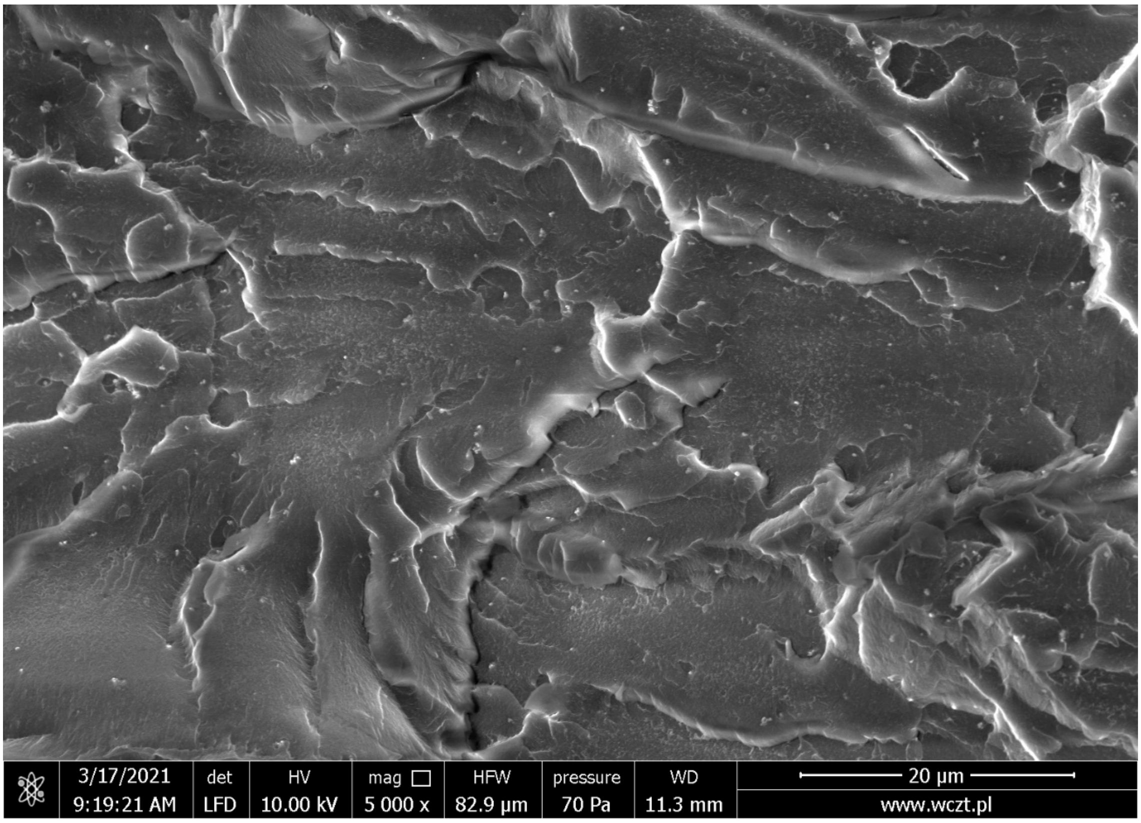

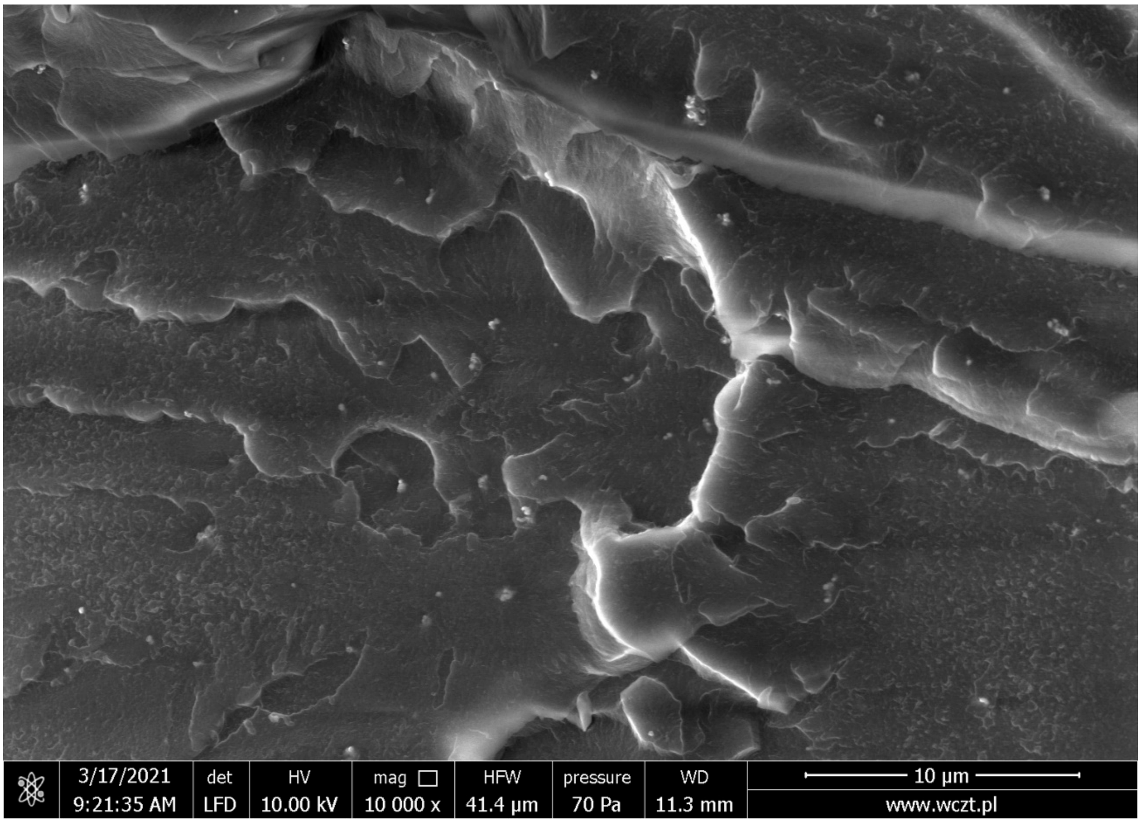

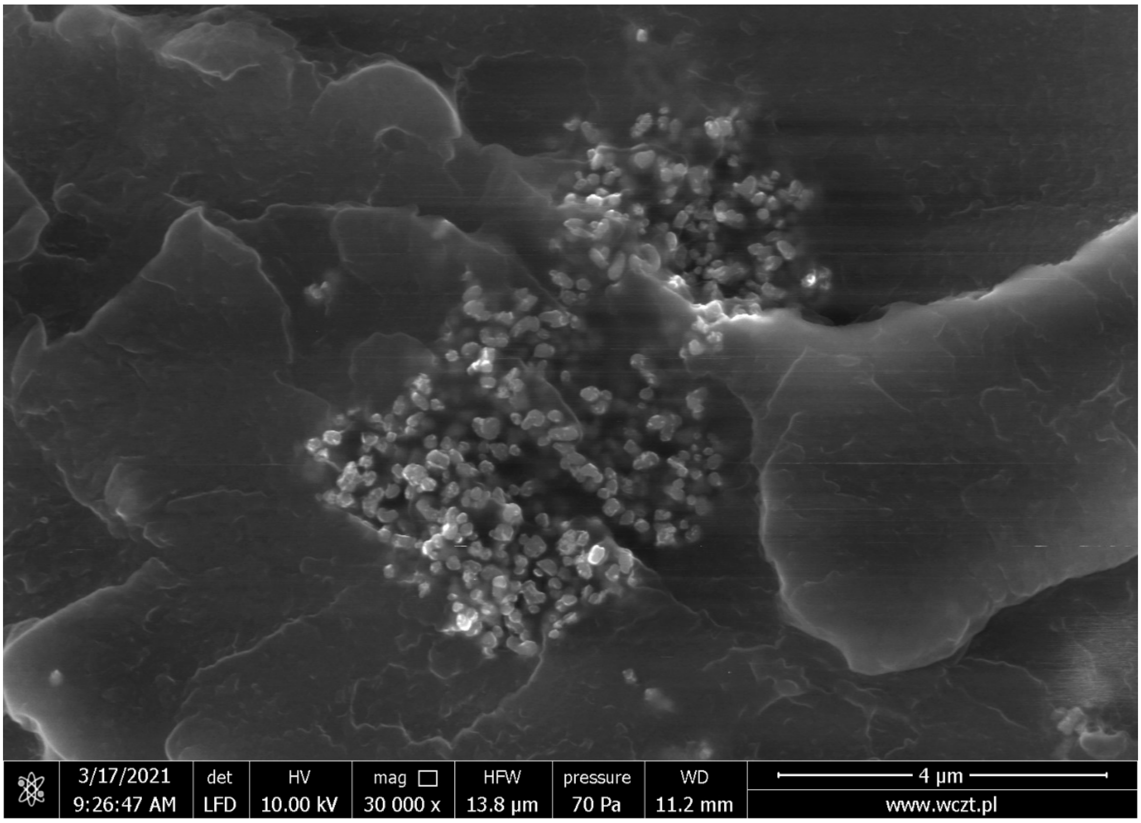
3/17/2021
9:26:47 AM
det
LFD
HV
10.00 kV
mag
30 000 x
HFW
13.8 μm
pressure
70 Pa
WD
11.2 mm
4 μm
www.wczt.pl

2%TiO₂, 0.5% iBuTMOS, mixing pump

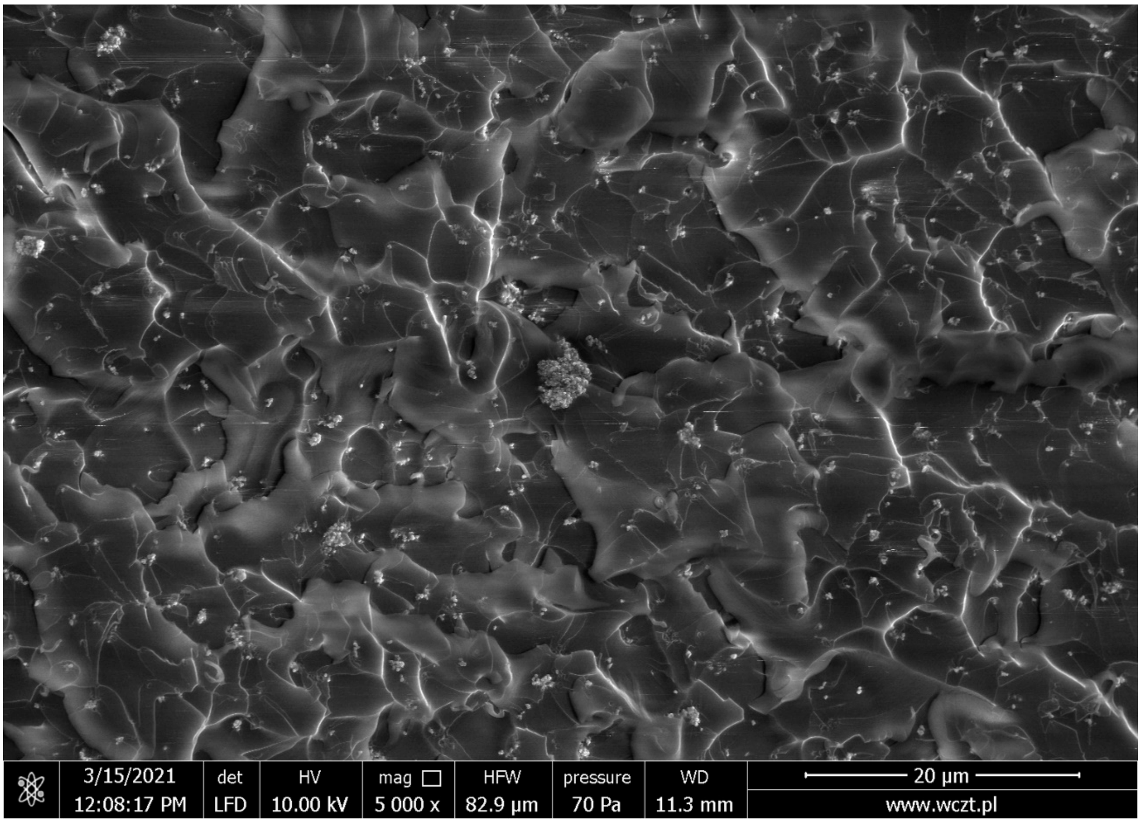

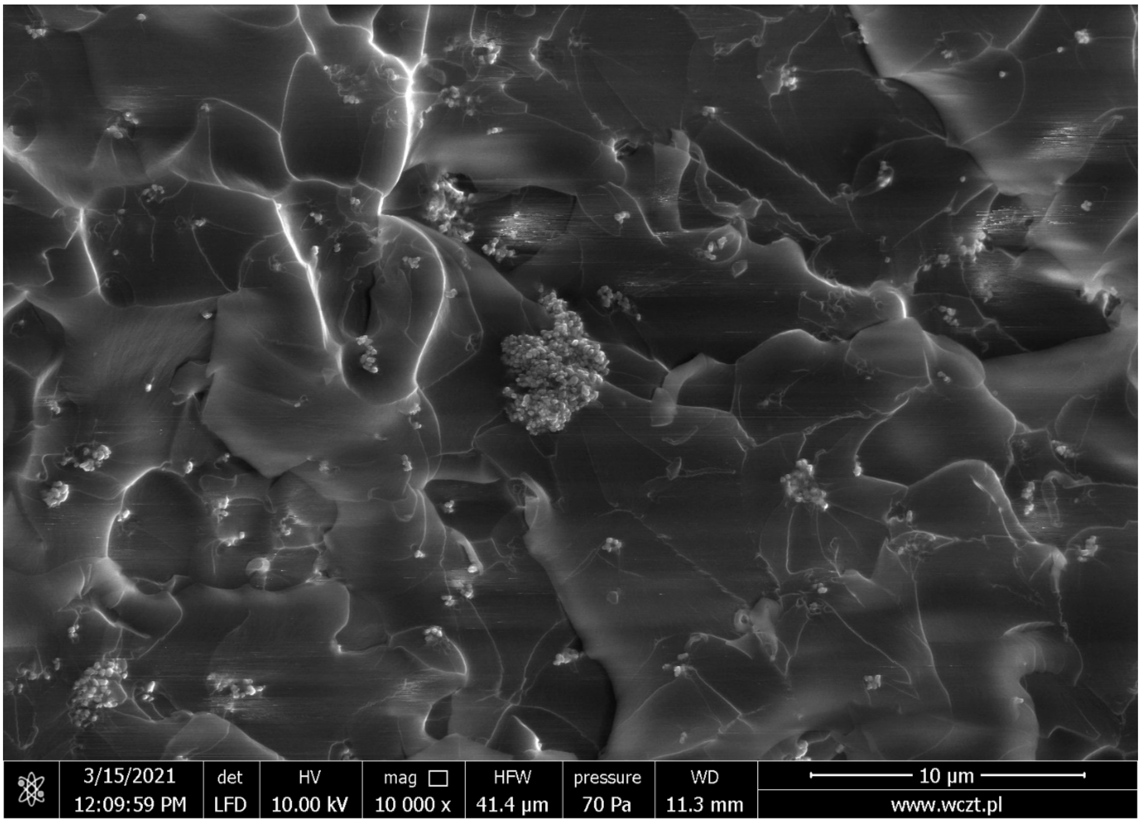

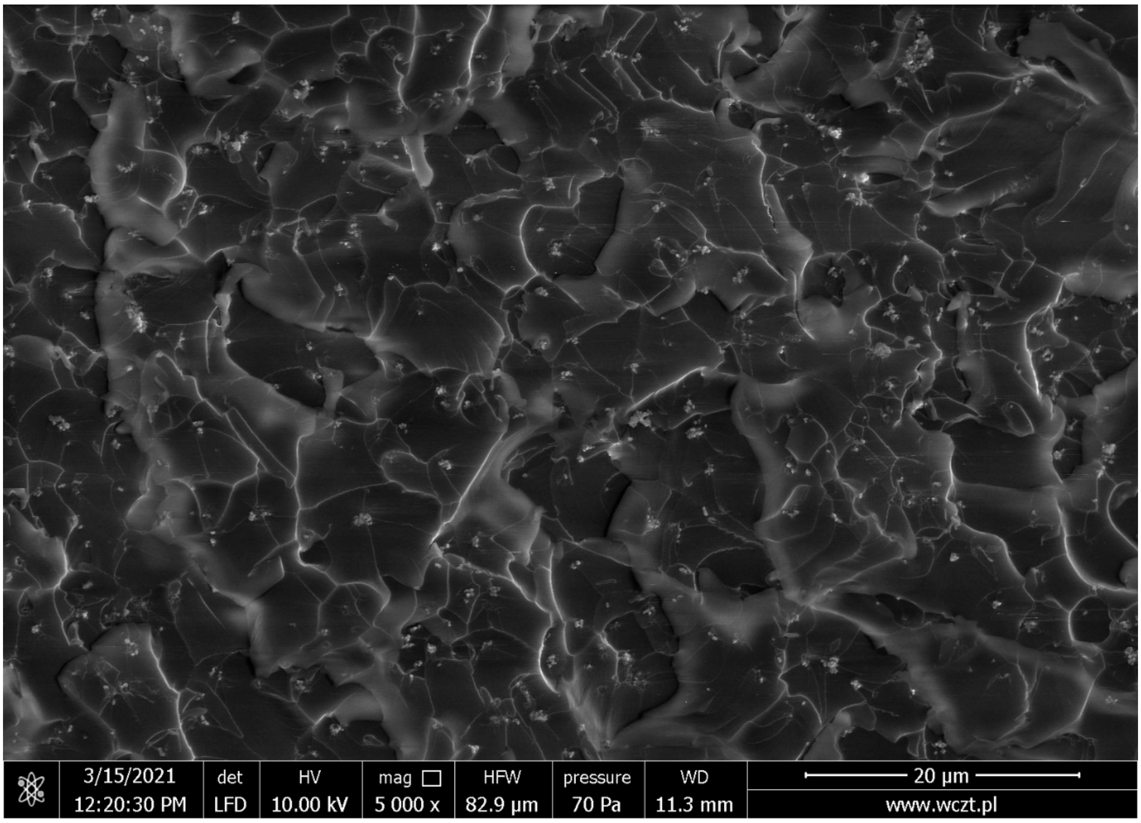
3/15/2021
12:20:30 PM
det
LFD
HV
10.00 kV
mag
5 000 x
HFW
82.9 µm
pressure
70 Pa
WD
11.3 mm
20 µm
www.wczt.pl

1%TiO₂, 1.5% iBuTMOS, mechanical stirrer

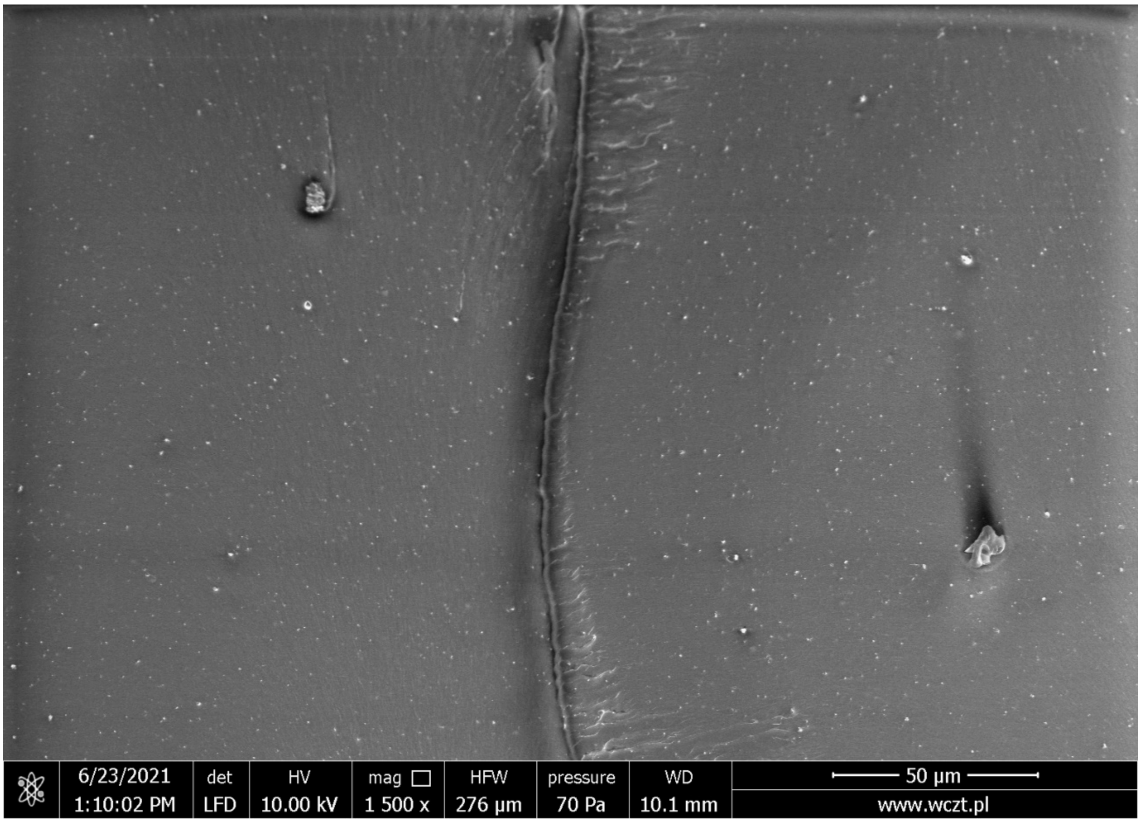

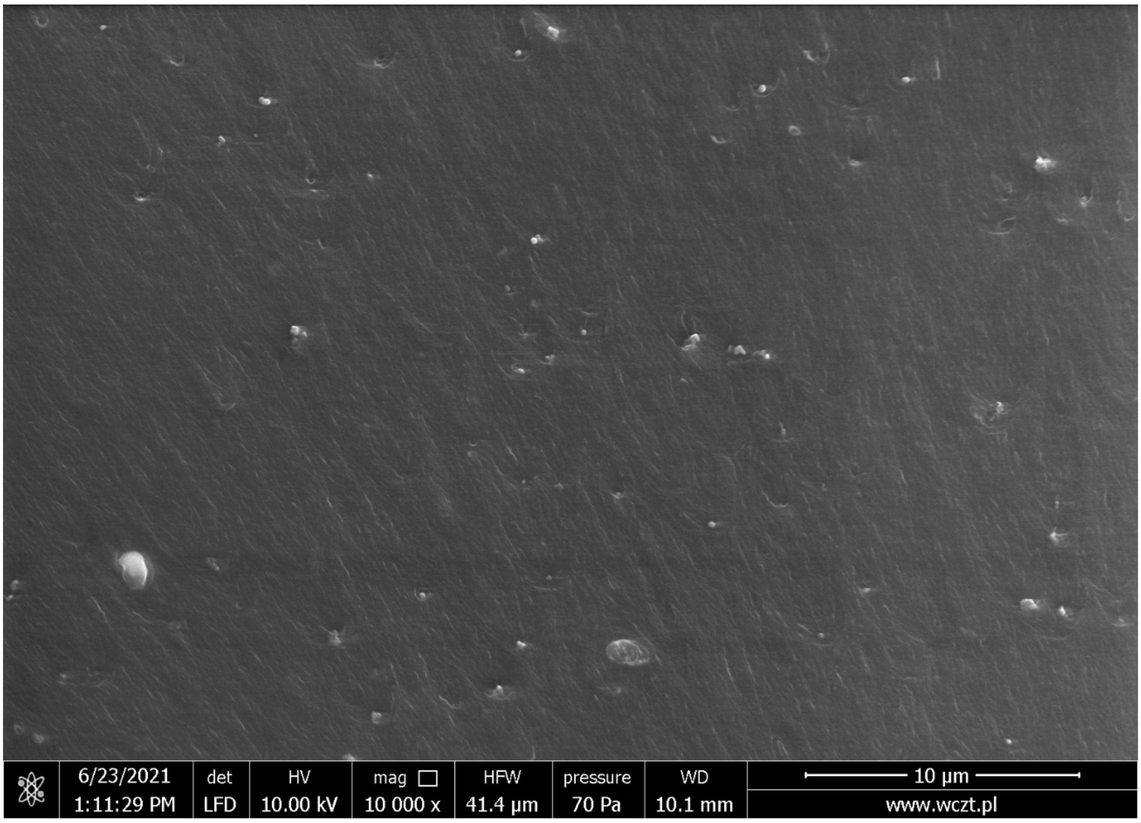

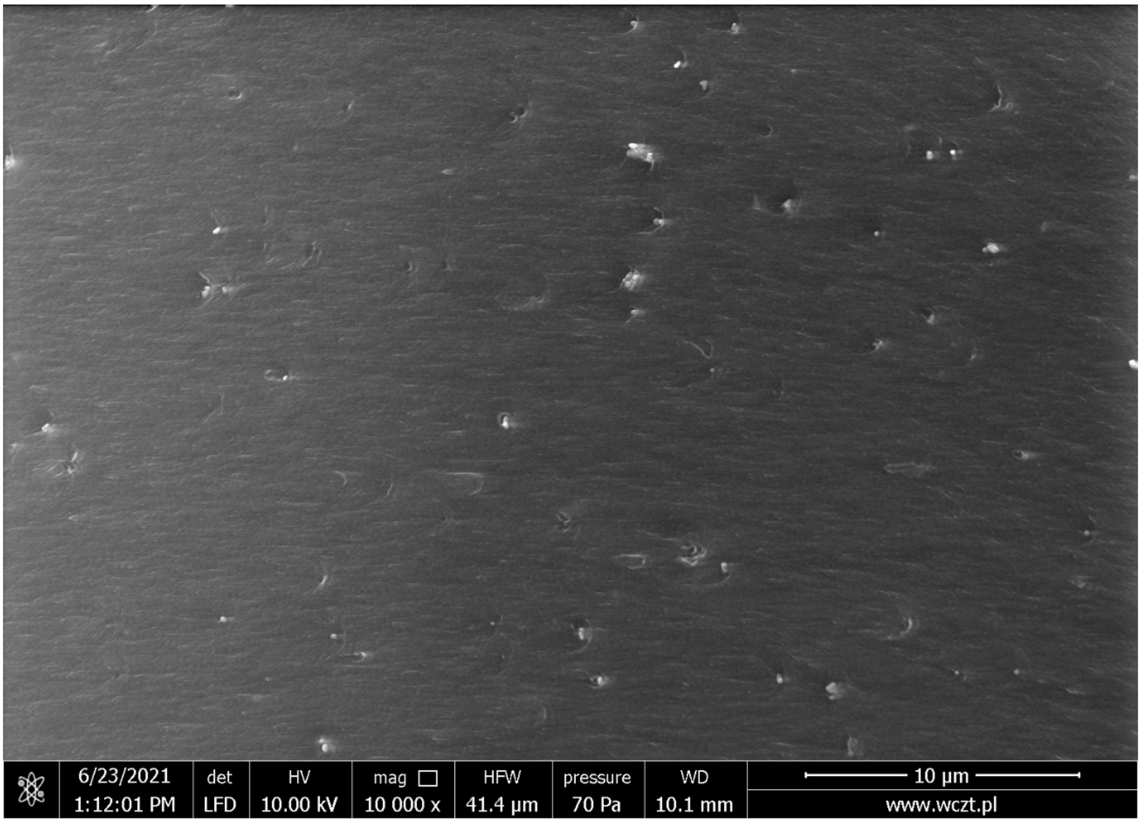
6/23/2021
1:12:01 PM
det
LFD
HV
10.00 kV
mag
10 000 x
HFW
41.4 µm
pressure
70 Pa
WD
10.1 mm
10 µm
www.wczt.pl

1%TiO₂, 1.5% iBuTMOS, mixing pump

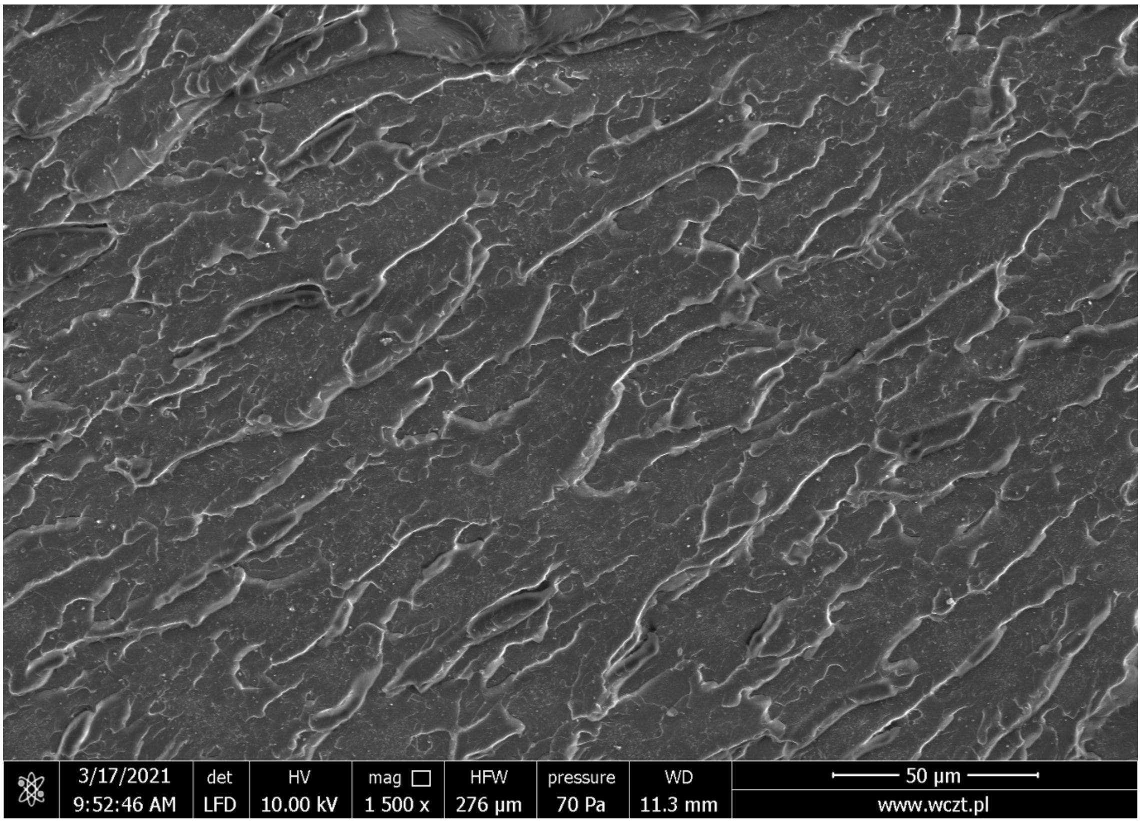

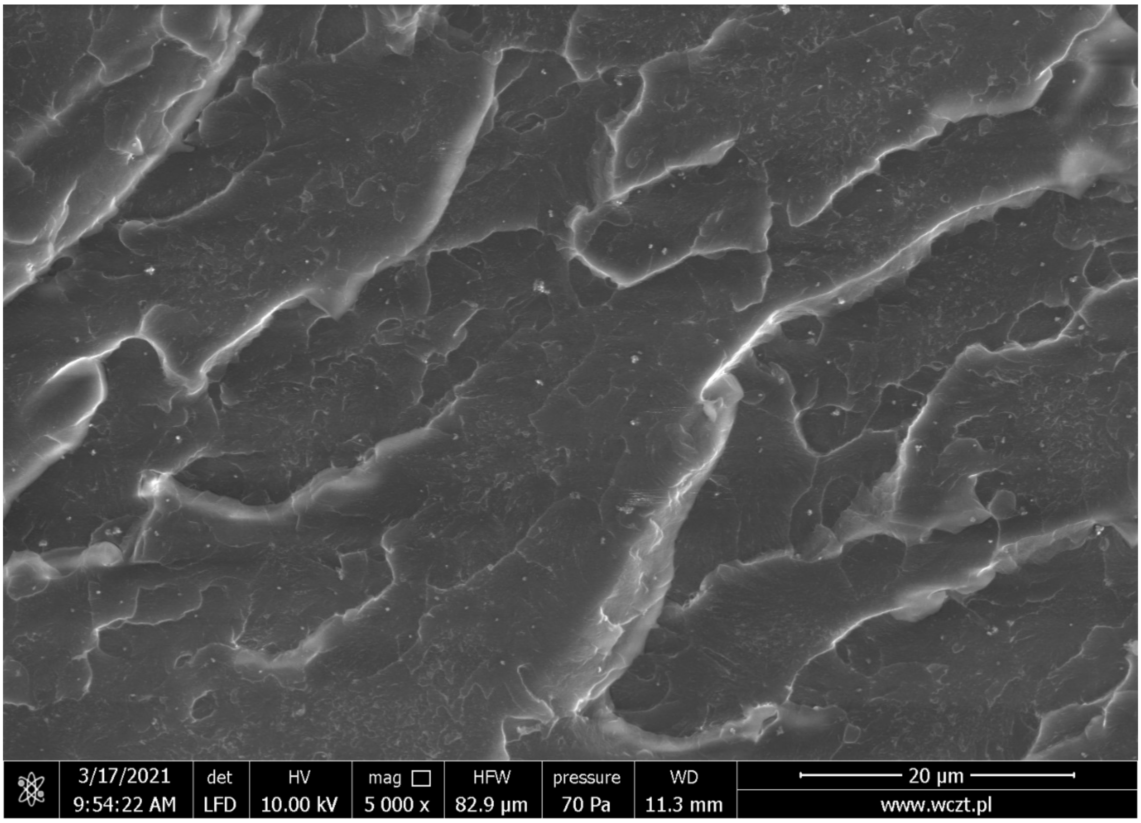

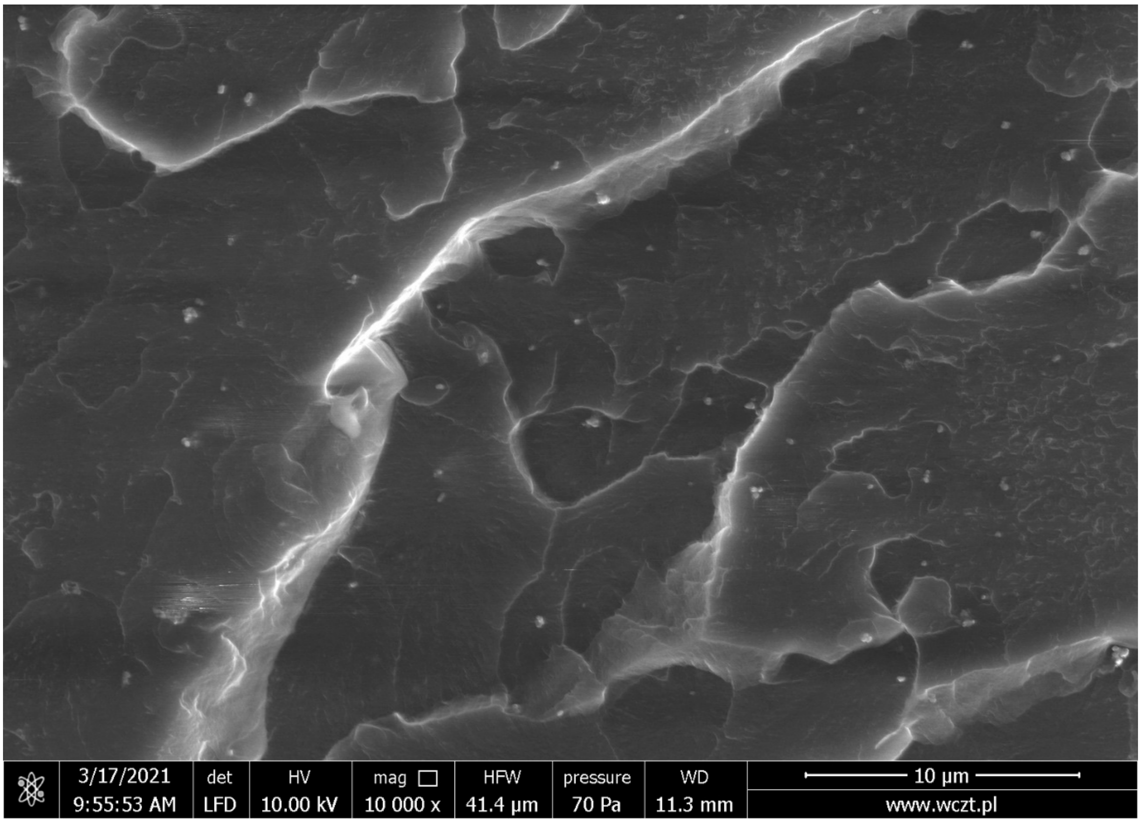
3/17/2021
9:55:53 AM
det
LFD
HV
10.00 kV
mag
10 000 x
HFW
41.4 µm
pressure
70 Pa
WD
11.3 mm
10 µm
www.wczt.pl

2%TiO_2, 1.5% iBuTMOS, mixing pump

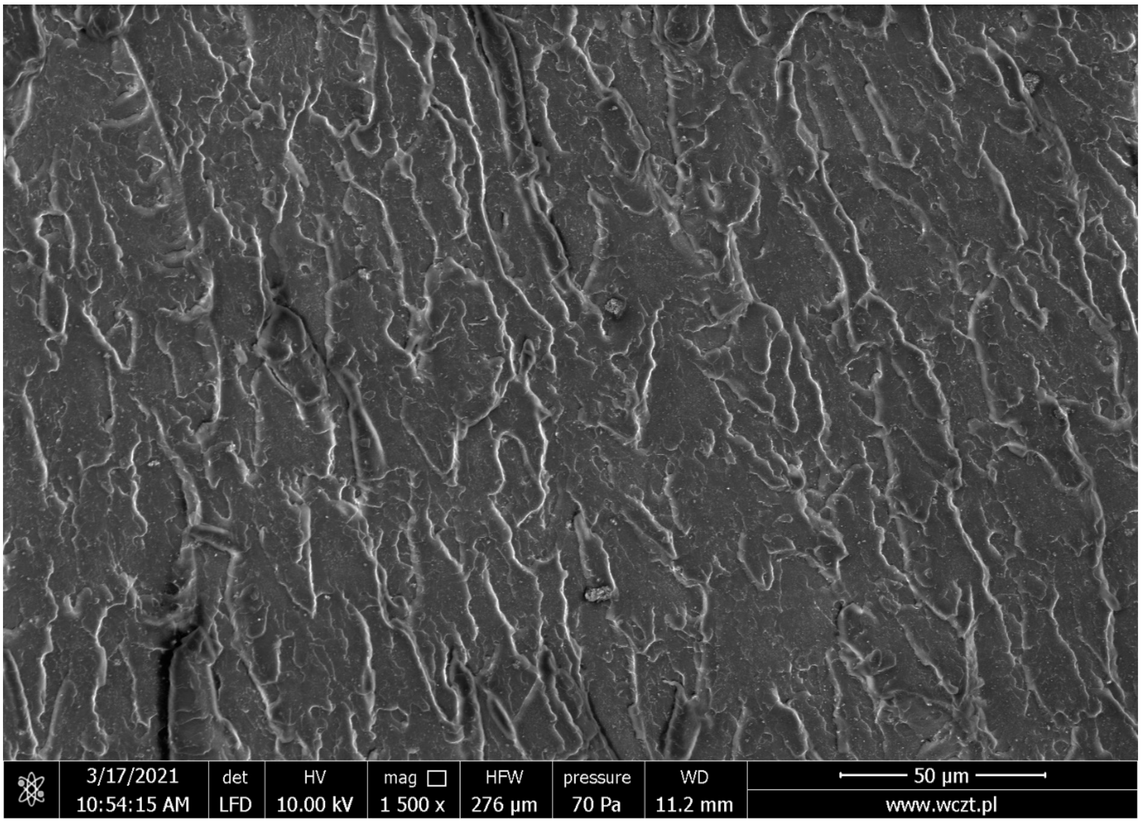

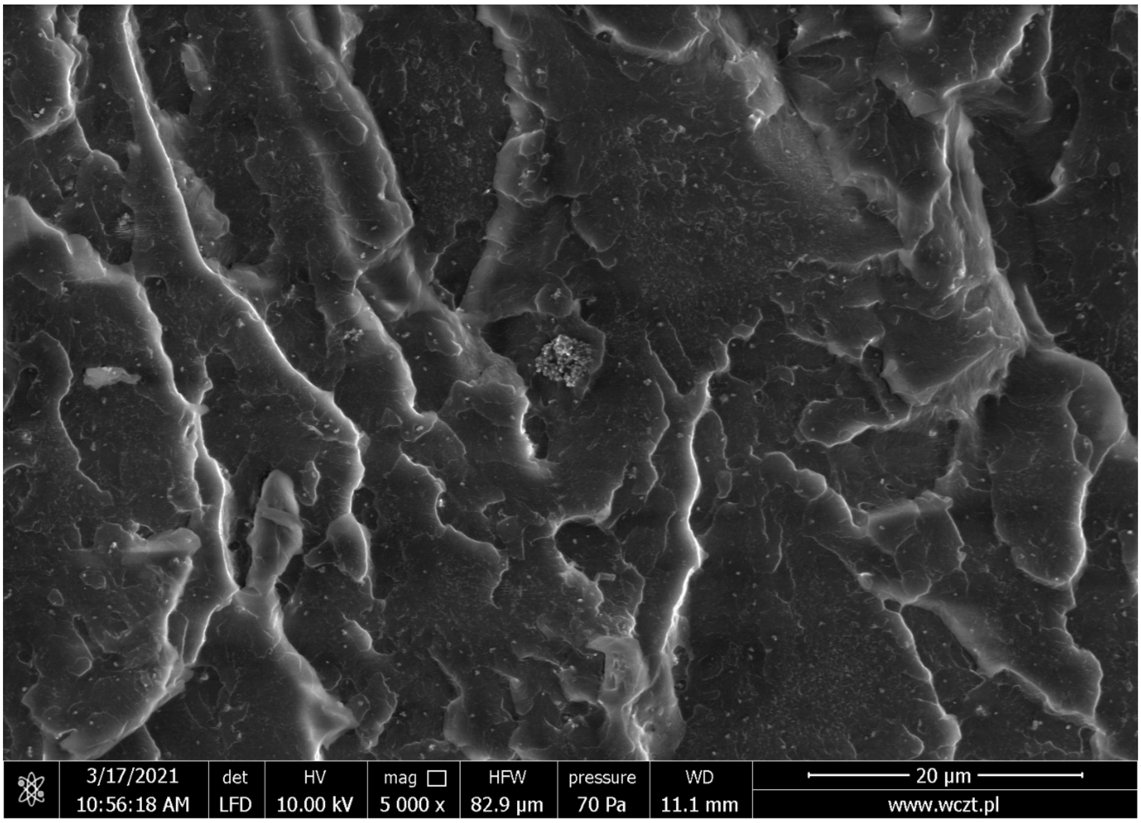

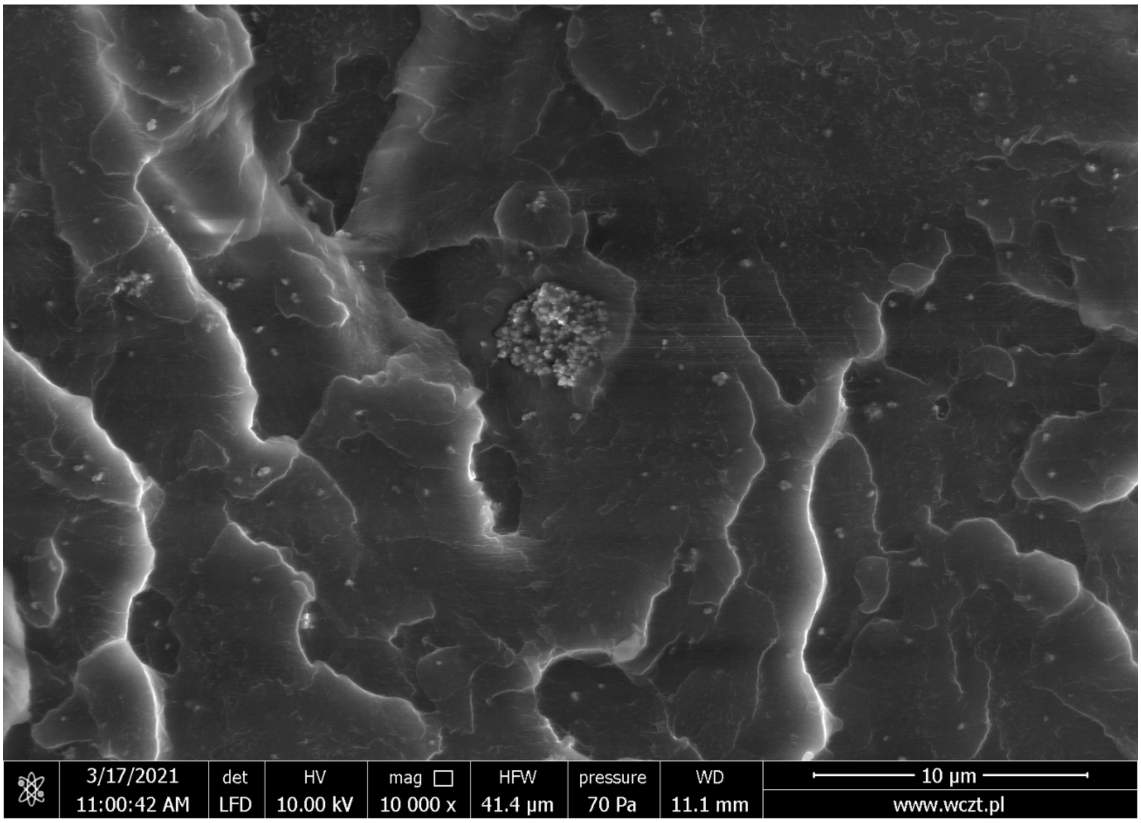
3/17/2021
11:00:42 AM
det
LFD
HV
10.00 kV
mag
10 000 x
HFW
41.4 µm
pressure
70 Pa
WD
11.1 mm
10 µm
www.wczt.pl

1%TiO_2, 0.5% GPTES, mixing pump

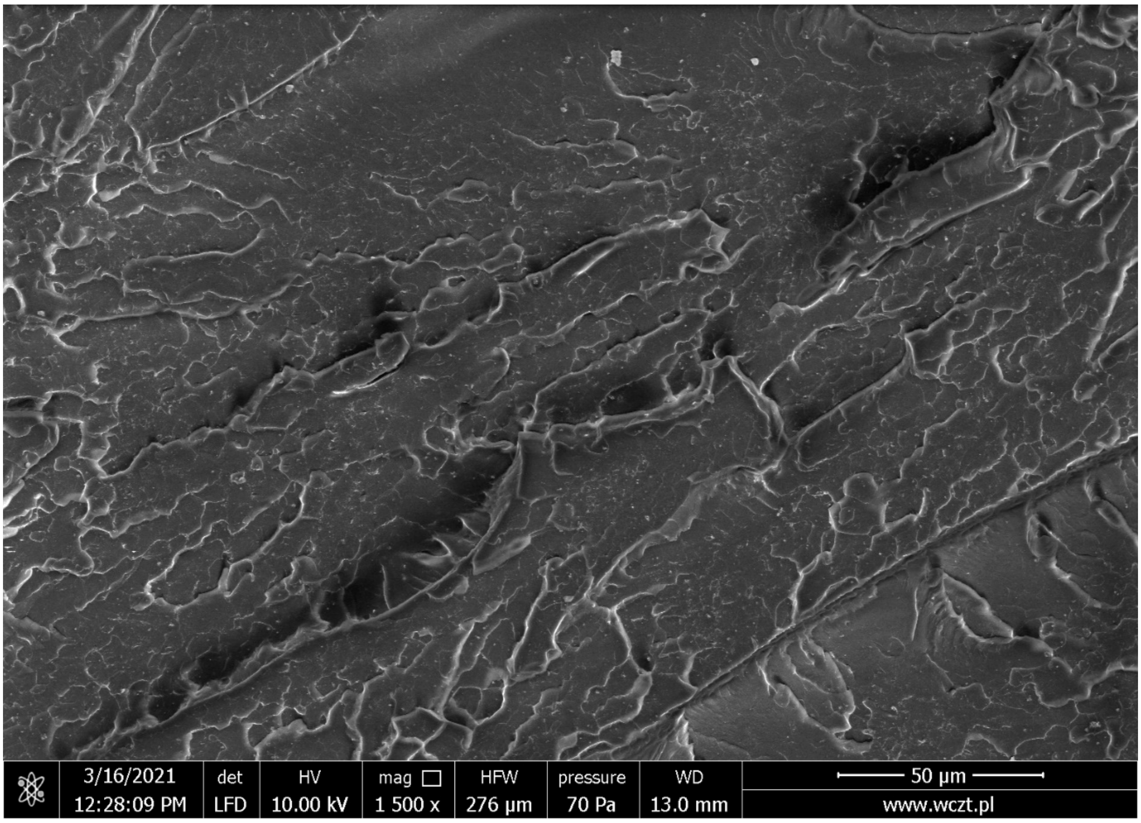

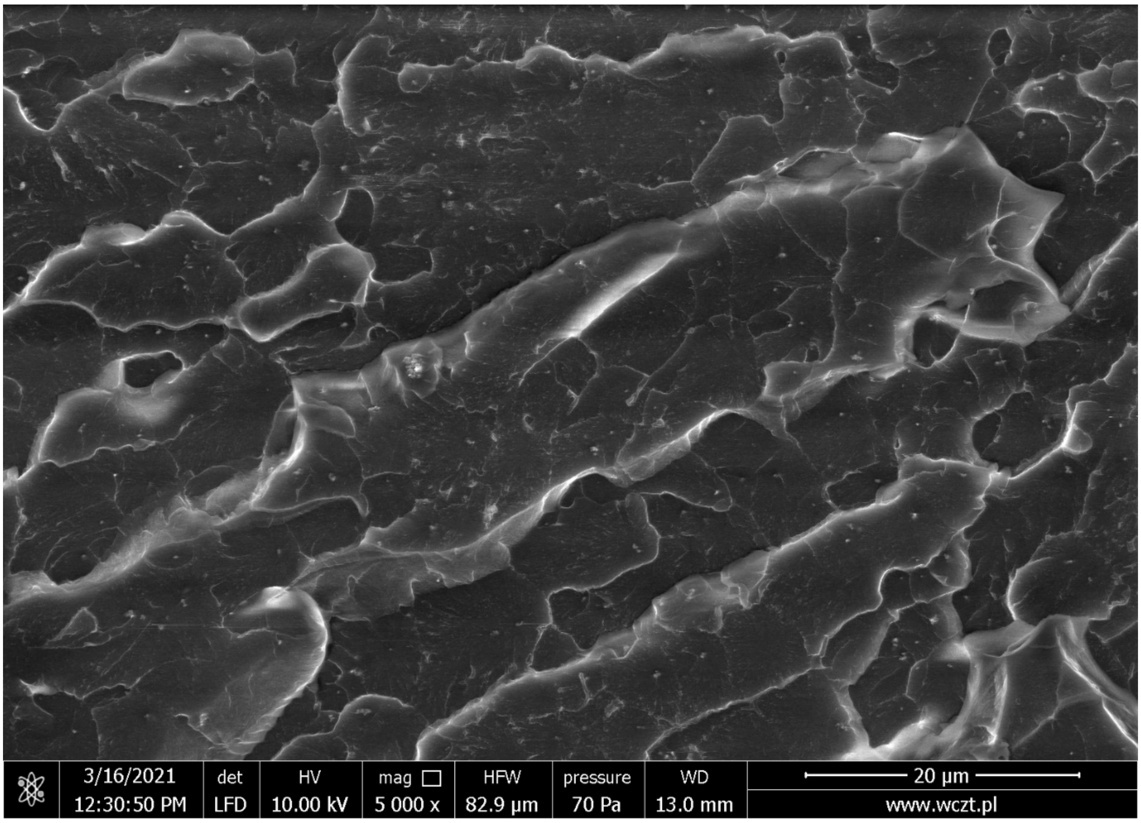

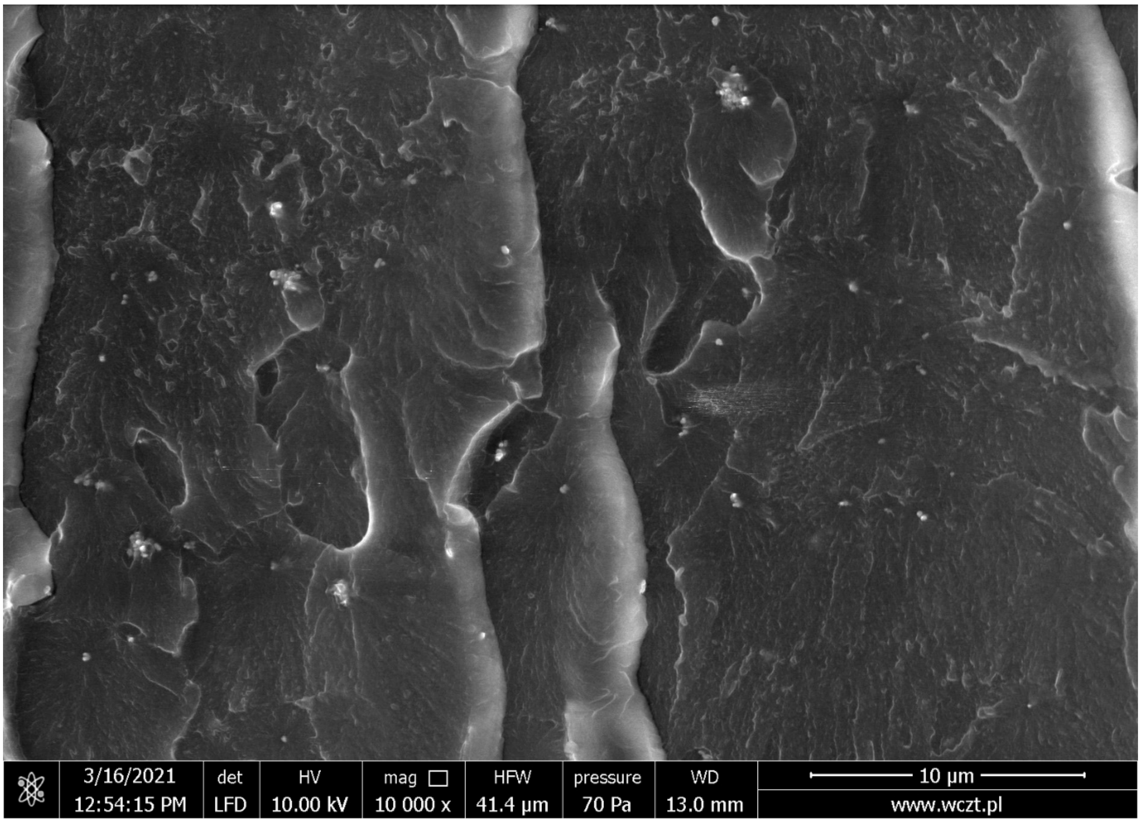
3/16/2021
12:54:15 PM
det
LFD
HV
10.00 kV
mag
10 000 x
HFW
41.4 µm
pressure
70 Pa
WD
13.0 mm
10 µm
www.wczt.pl

2%TiO₂, 0.5% GPTES, mixing pump

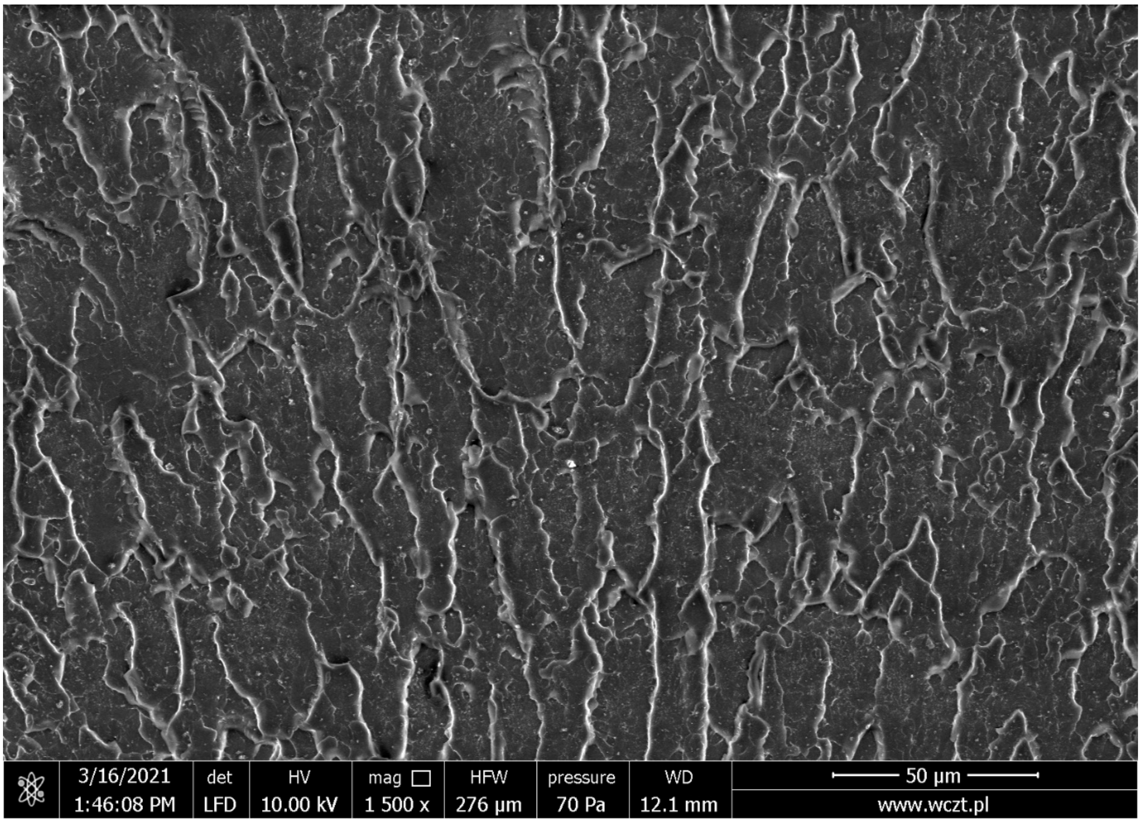

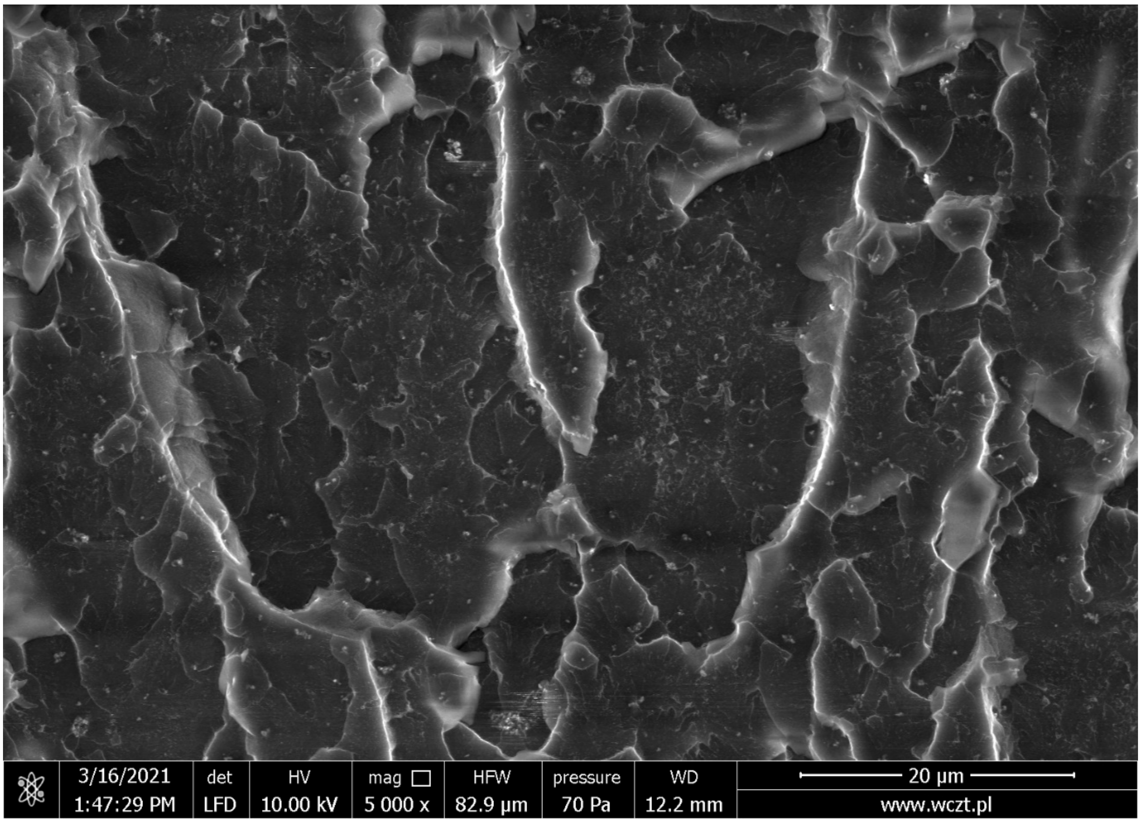

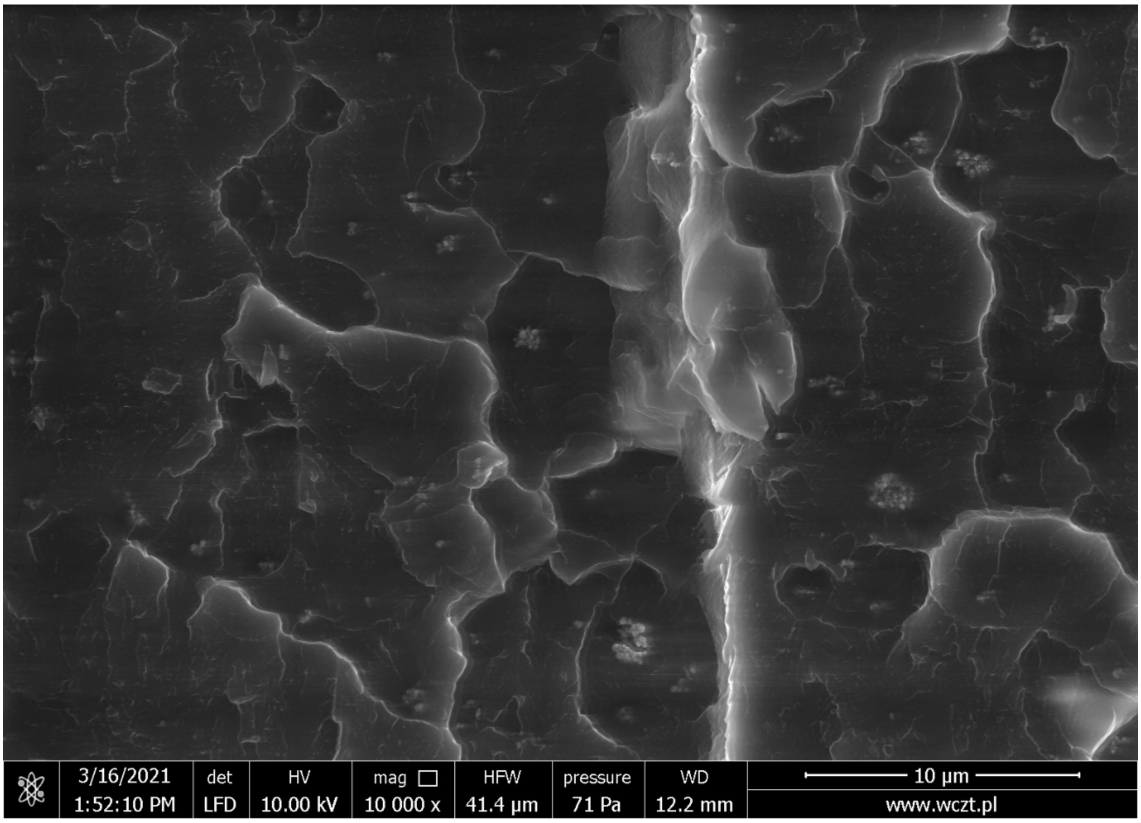
3/16/2021
1:52:10 PM
det
LFD
HV
10.00 kV
mag
10 000 x
HFW
41.4 µm
pressure
71 Pa
WD
12.2 mm
10 µm
www.wczt.pl

1%TiO₂, 1.5% GPTES, mechanical stirrer

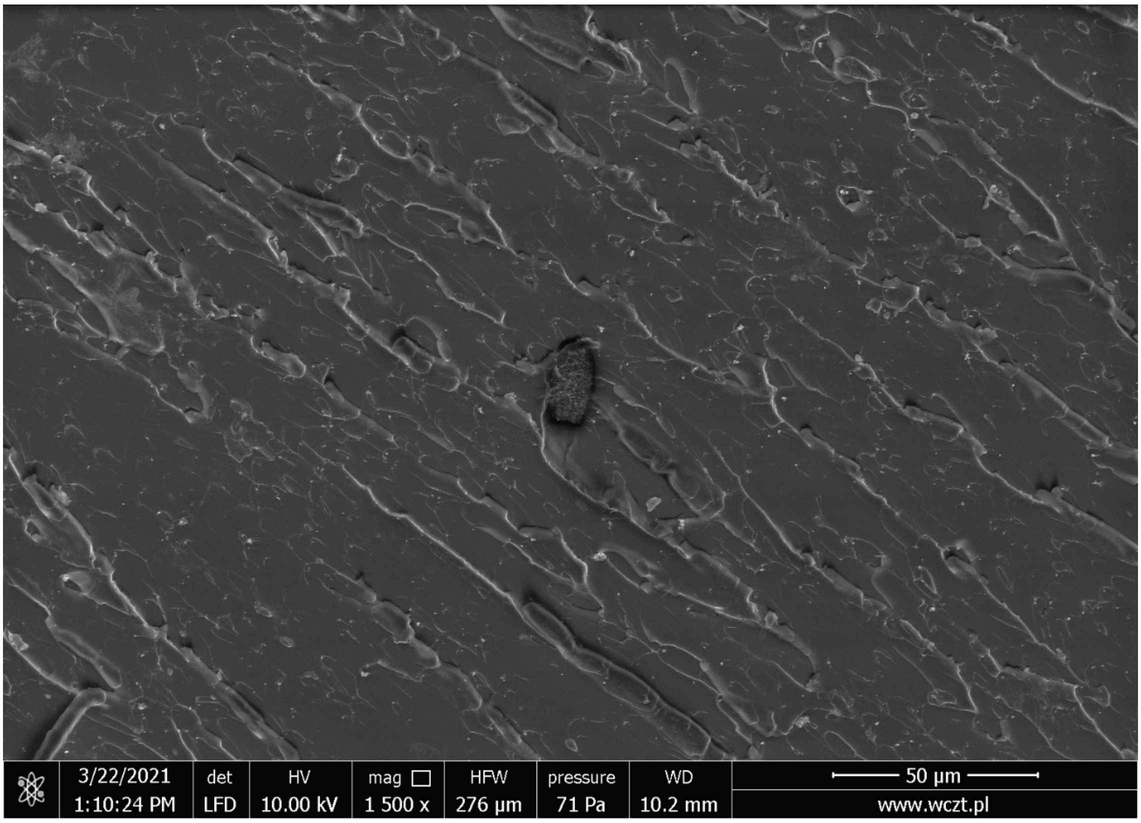

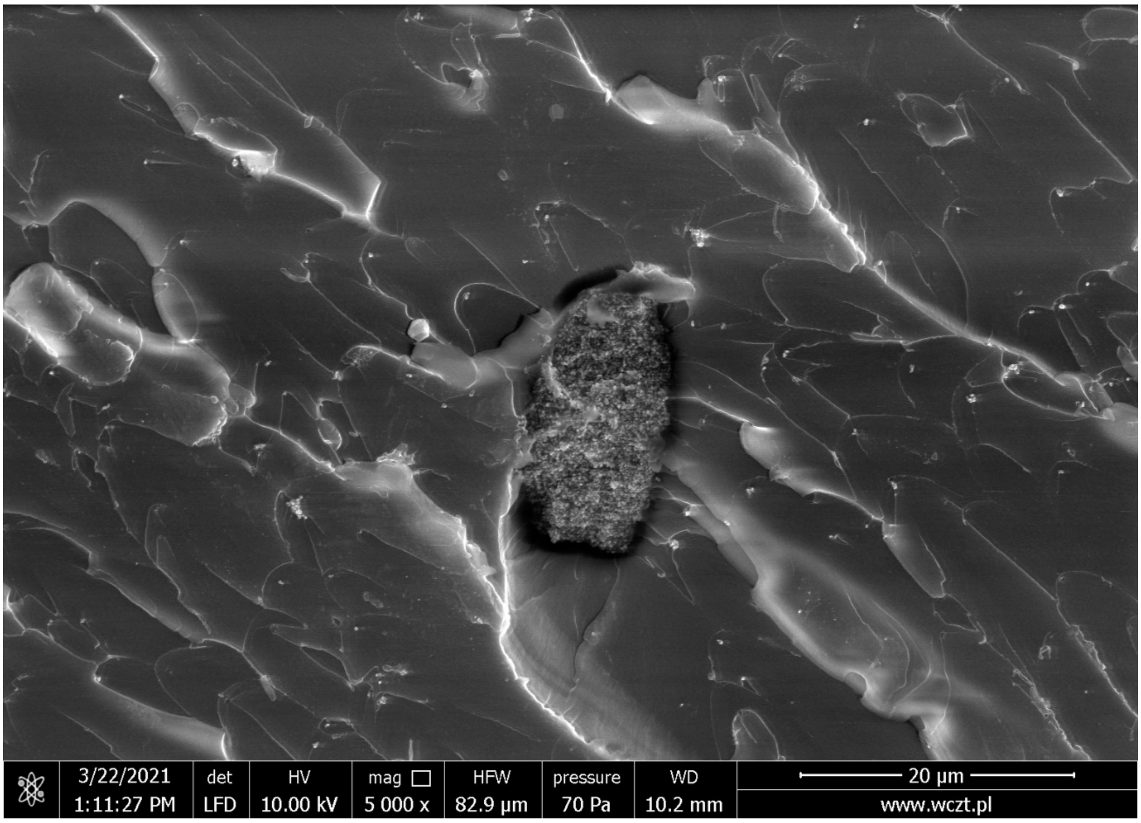

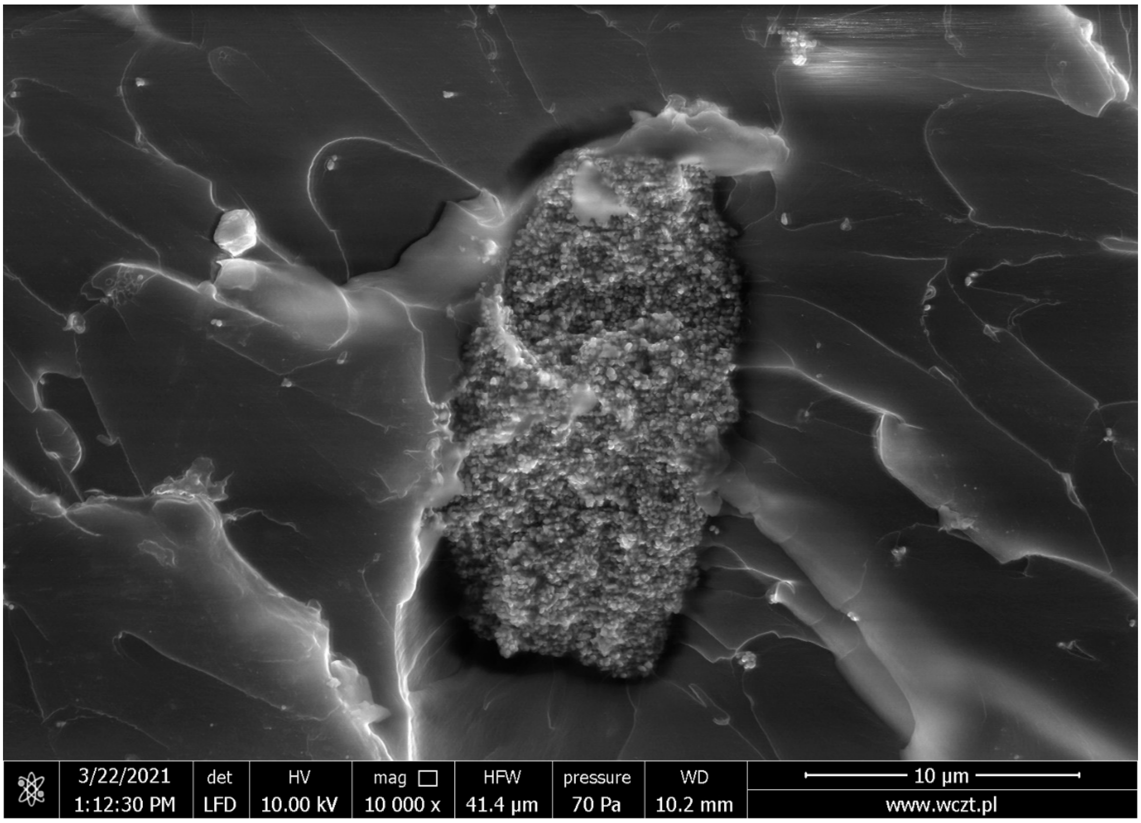
3/22/2021
1:12:30 PM
det
LFD
HV
10.00 kV
mag
10 000 x
HFW
41.4 µm
pressure
70 Pa
WD
10.2 mm
10 µm
www.wczt.pl

1%TiO₂, 1.5% GPTES, mixing pump

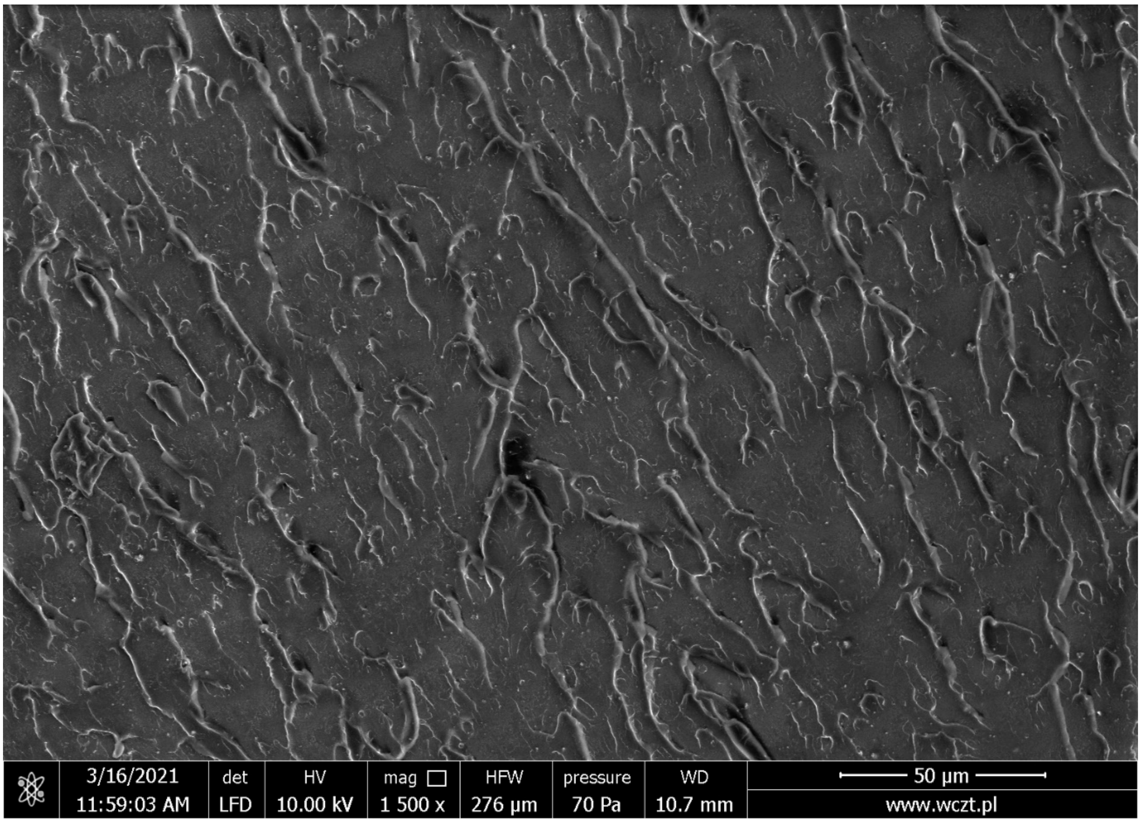

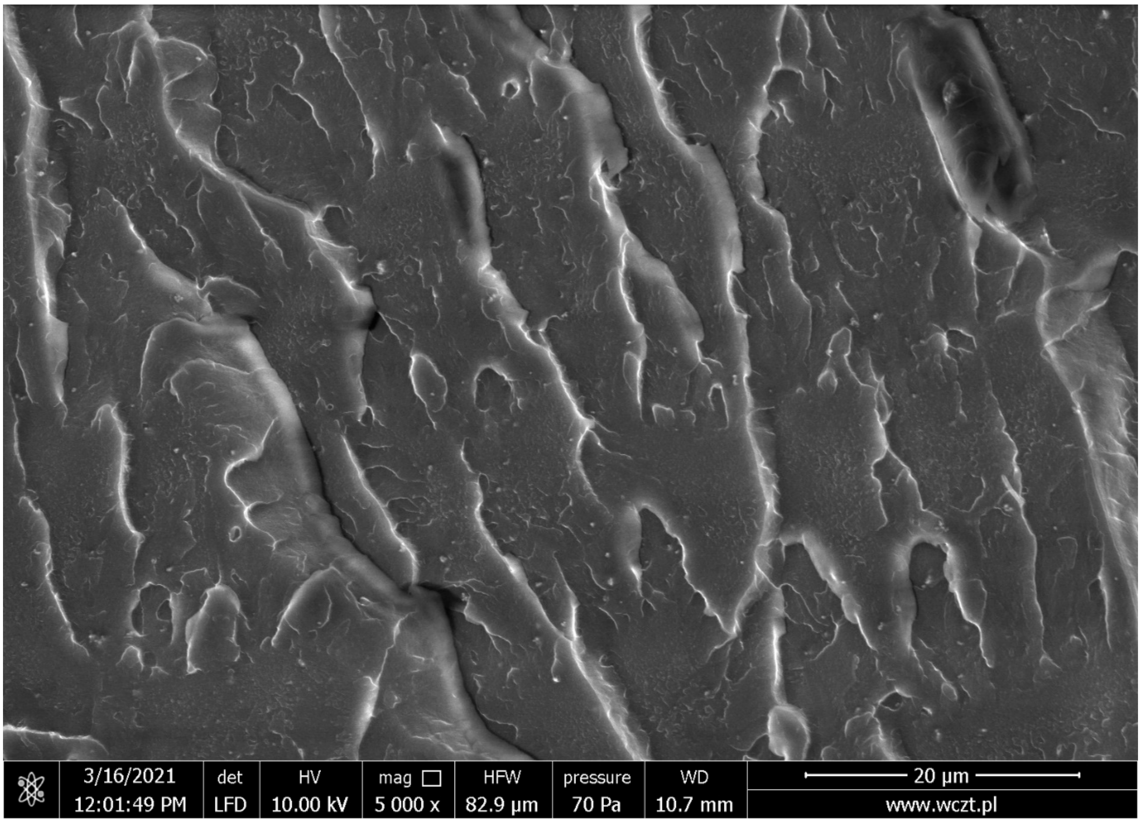

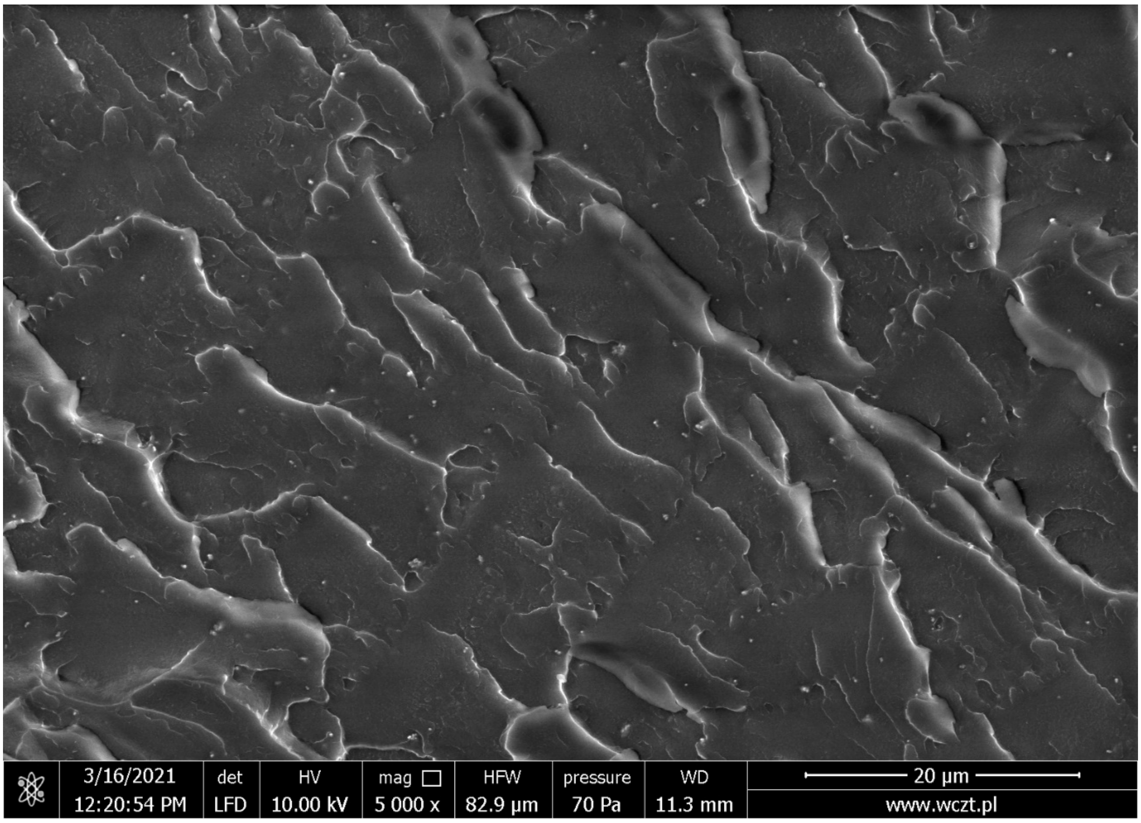
3/16/2021
12:20:54 PM
det
LFD
HV
10.00 kV
mag
5 000 x
HFW
82.9 µm
pressure
70 Pa
WD
11.3 mm
20 µm
www.wczt.pl

2%TiO_2, 1.5% GPTES, mixing pump

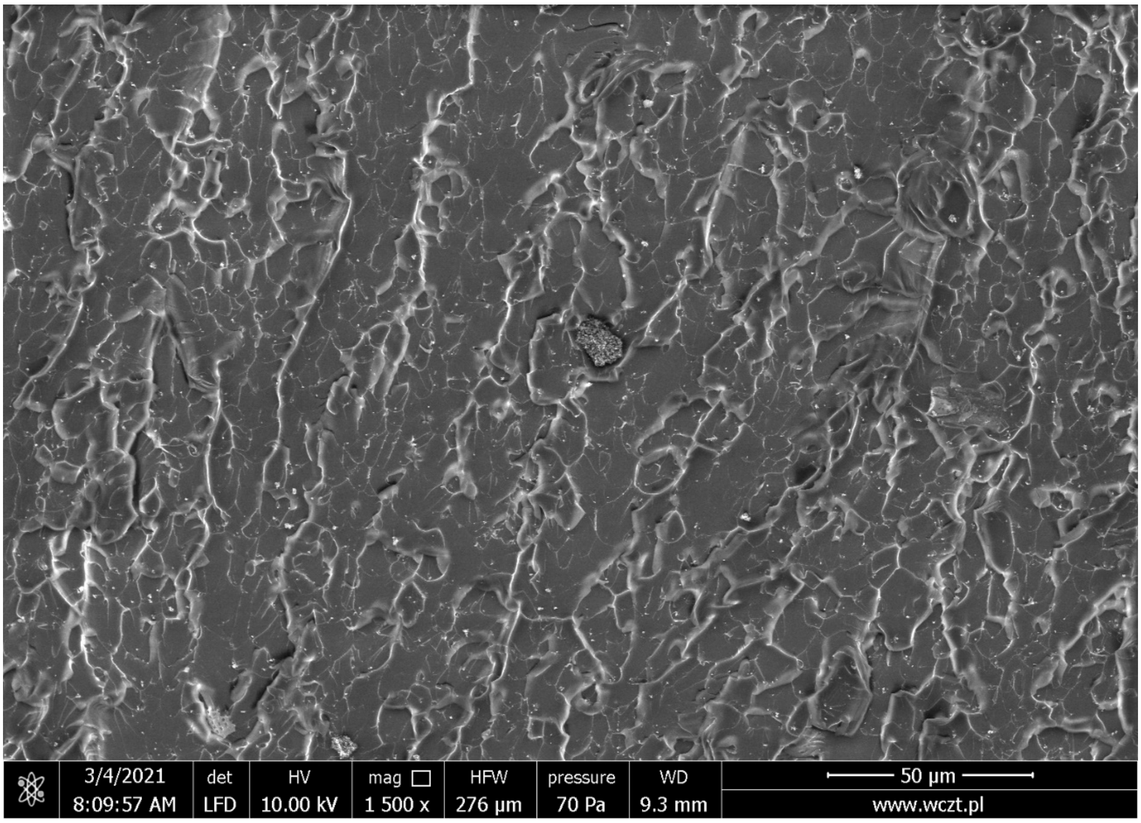

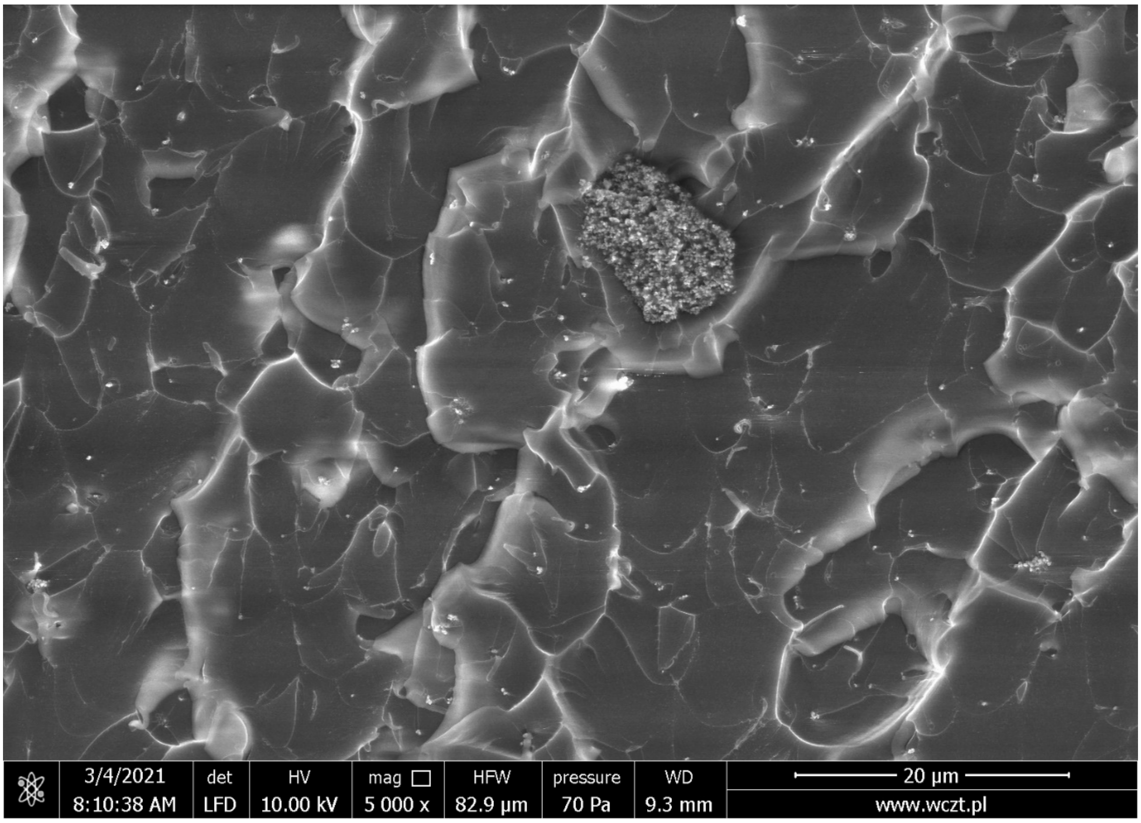

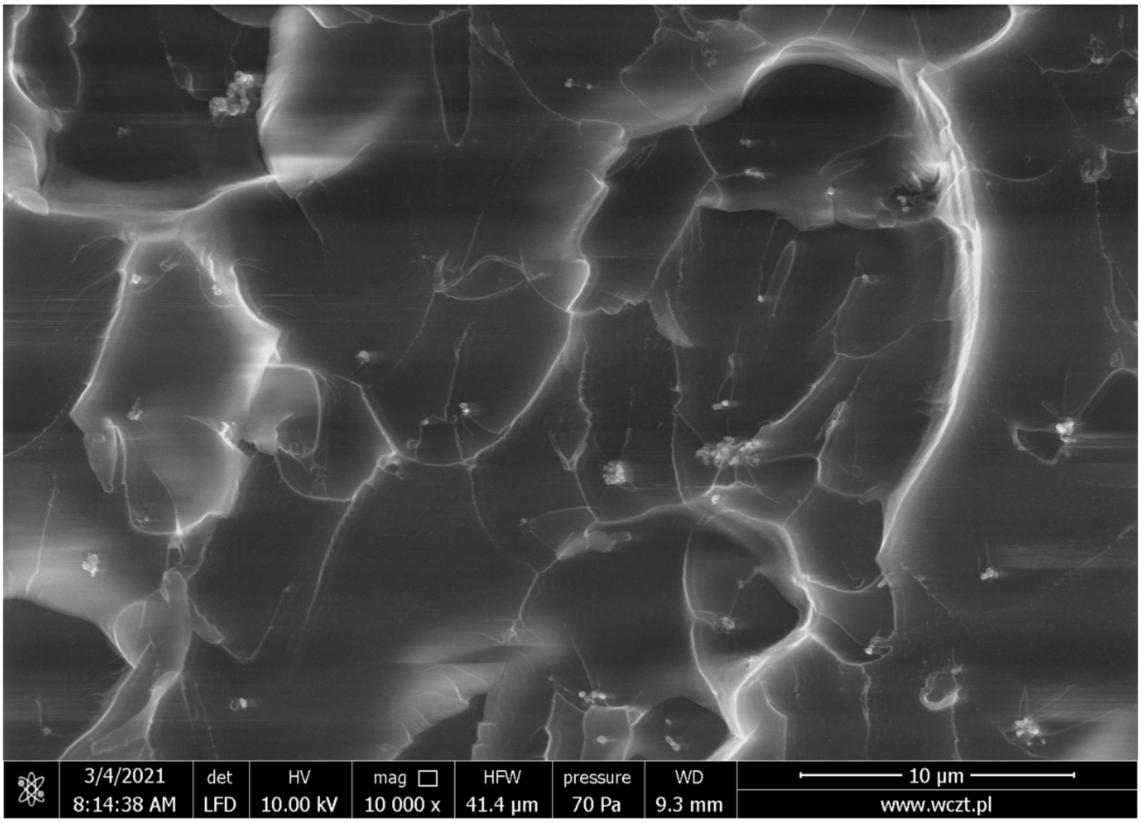
3/4/2021
8:14:38 AM
det
LFD
HV
10.00 kV
mag
10 000 x
HFW
41.4 µm
pressure
70 Pa
WD
9.3 mm
10 µm
www.wczt.pl

1%TiO_2, 0.5% iBu_7SSQ-OEt, mixing pump

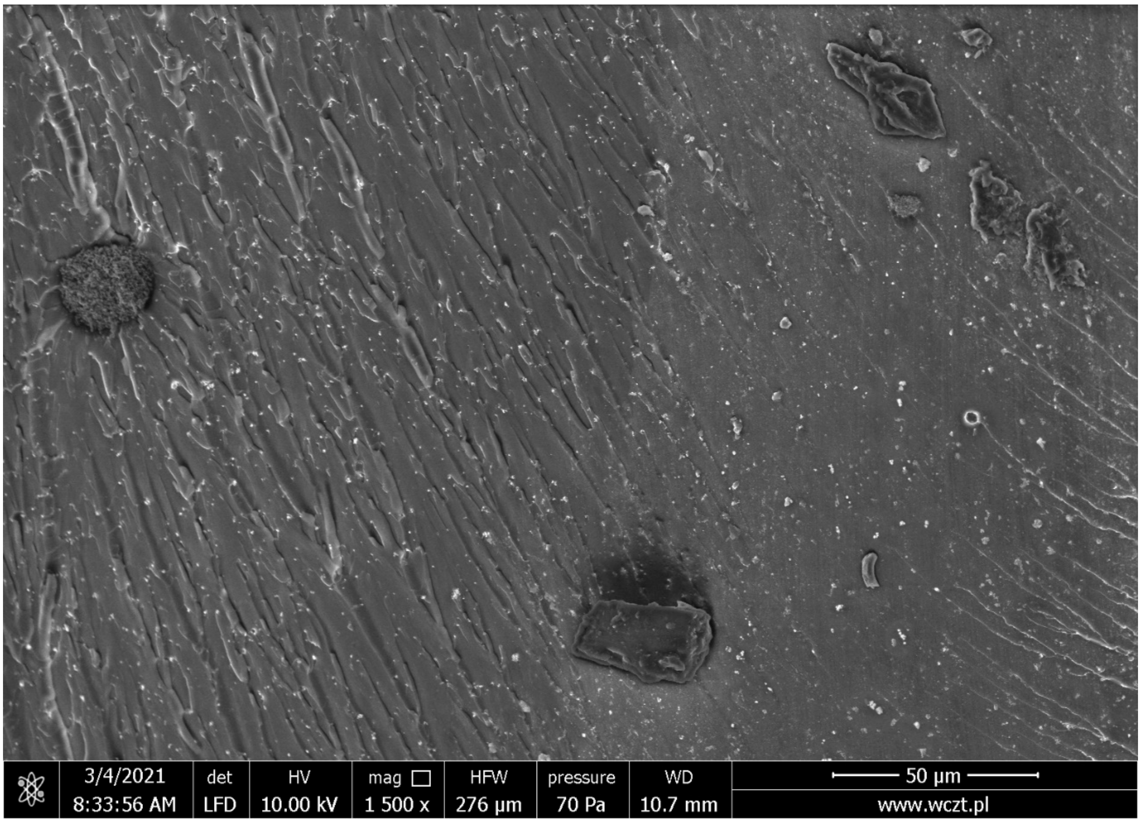

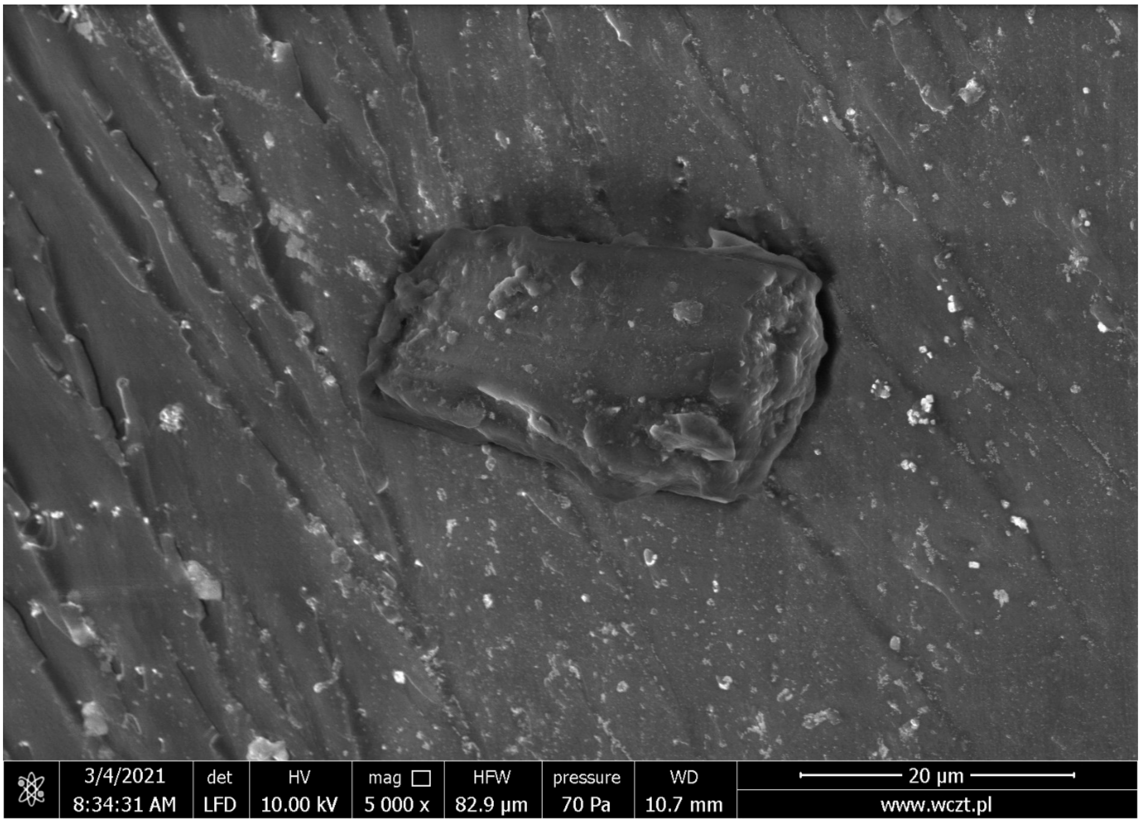

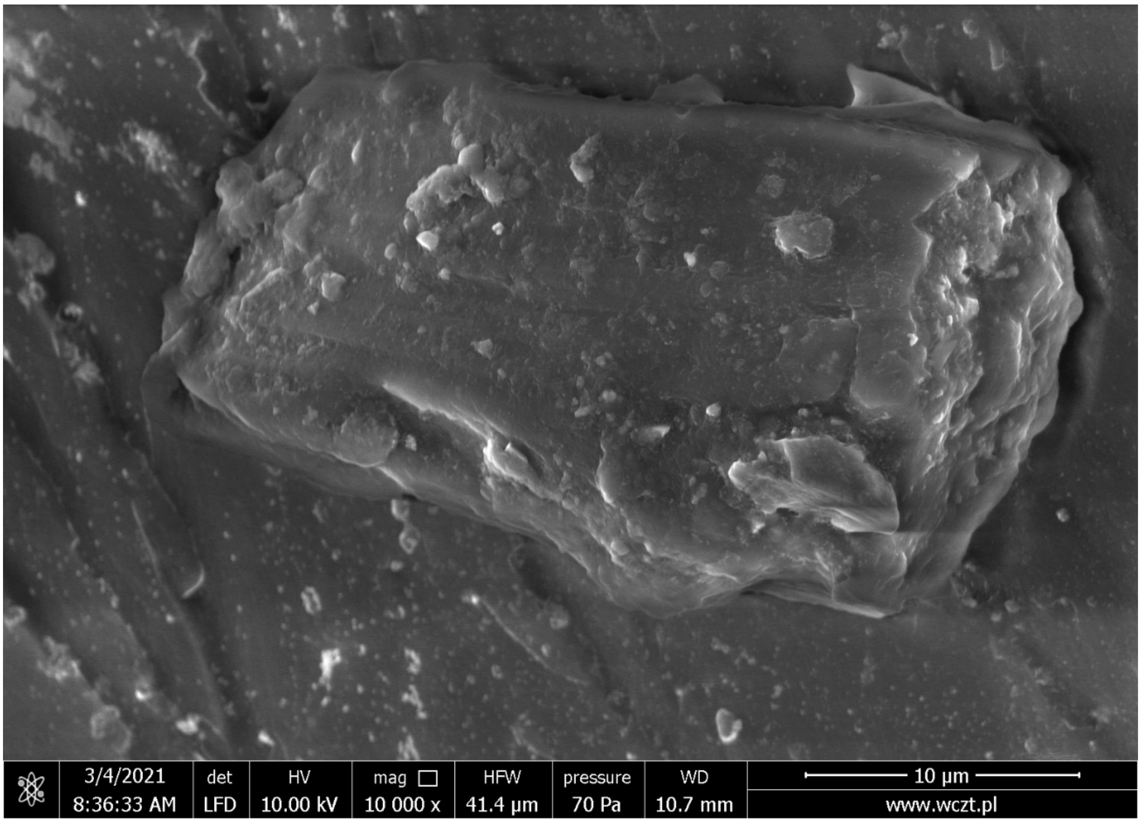
3/4/2021
8:36:33 AM
det
LFD
HV
10.00 kV
mag
10 000 x
HFW
41.4 µm
pressure
70 Pa
WD
10.7 mm
10 µm
www.wczt.pl

2%TiO_2, 0.5% iBu_7SSQ-OEt, mixing pump

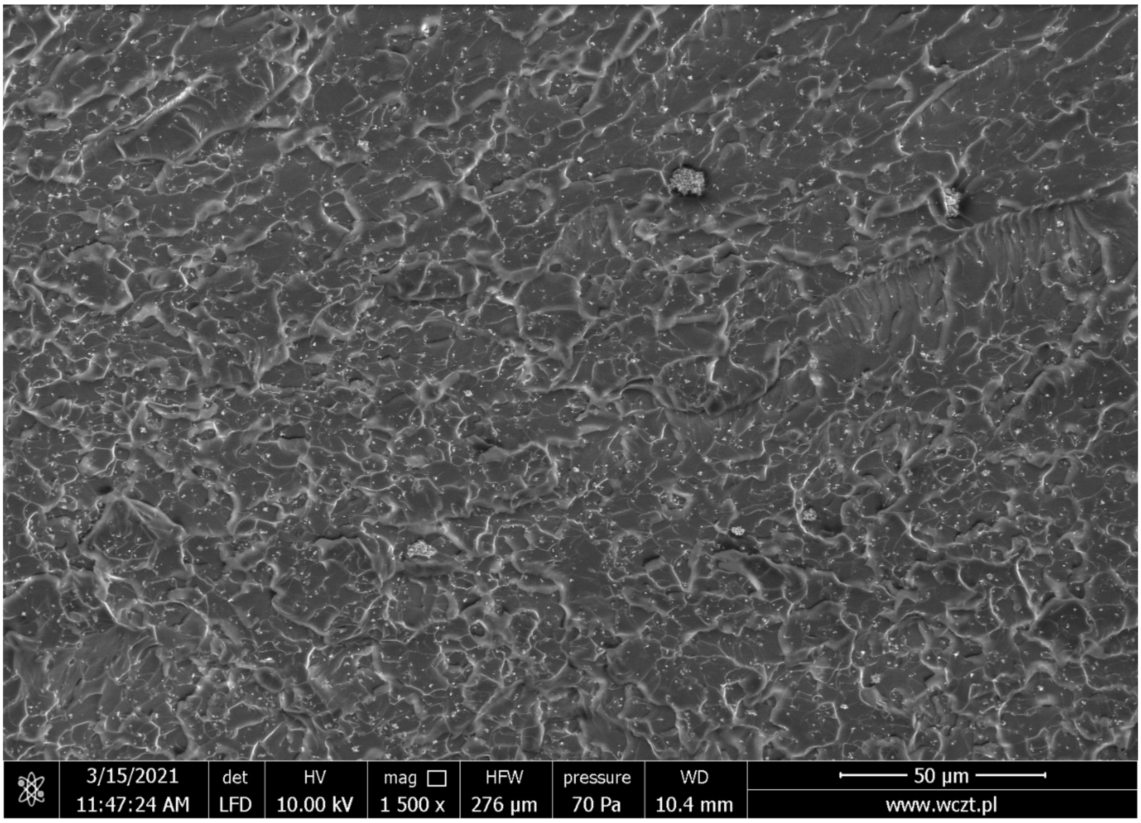

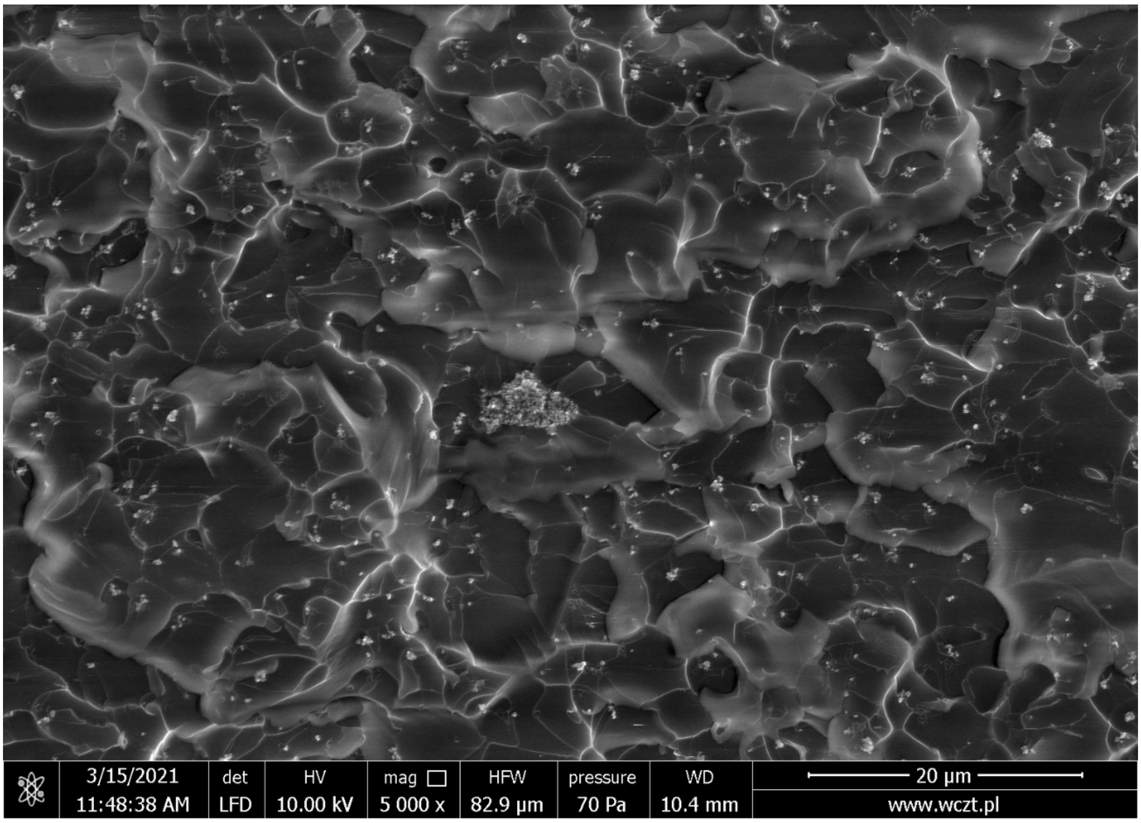

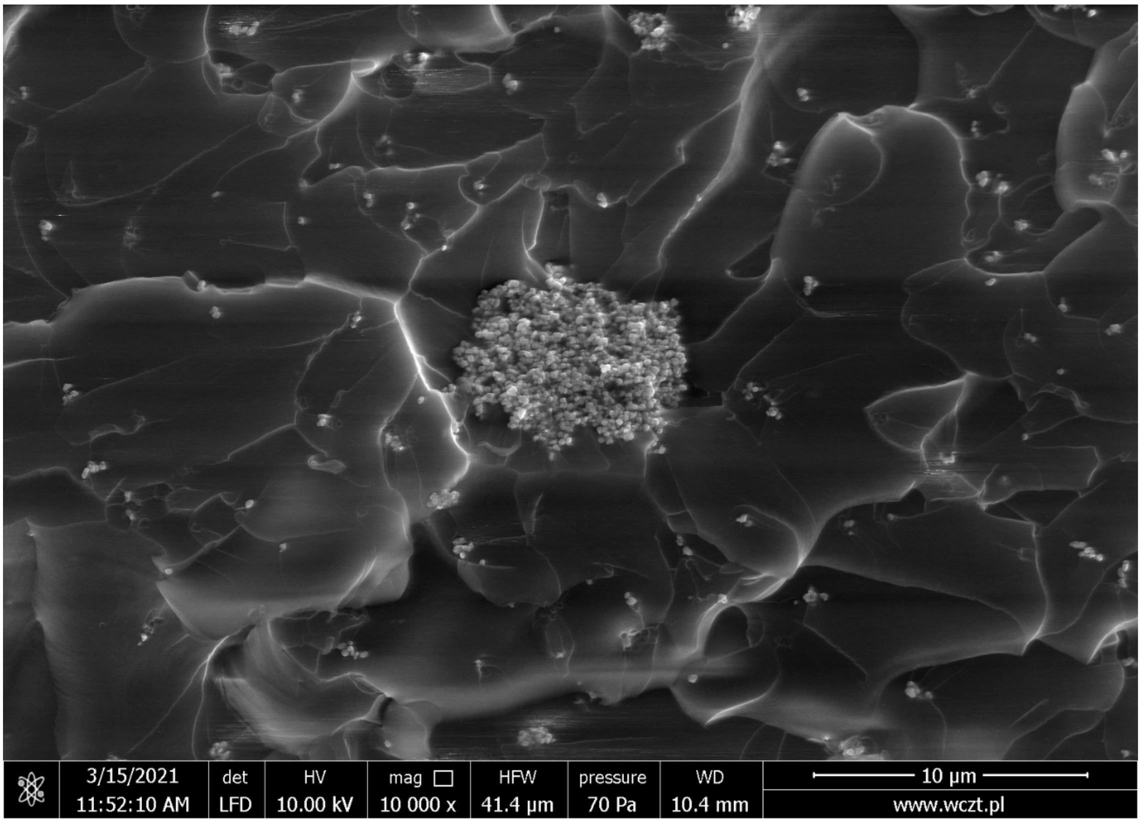
3/15/2021
11:52:10 AM
det
LFD
HV
10.00 kV
mag
10 000 x
HFW
41.4 µm
pressure
70 Pa
WD
10.4 mm
10 µm
www.wczt.pl

1%TiO_2, 1.5% iBu_7SSQ-OEt, mechanical stirrer

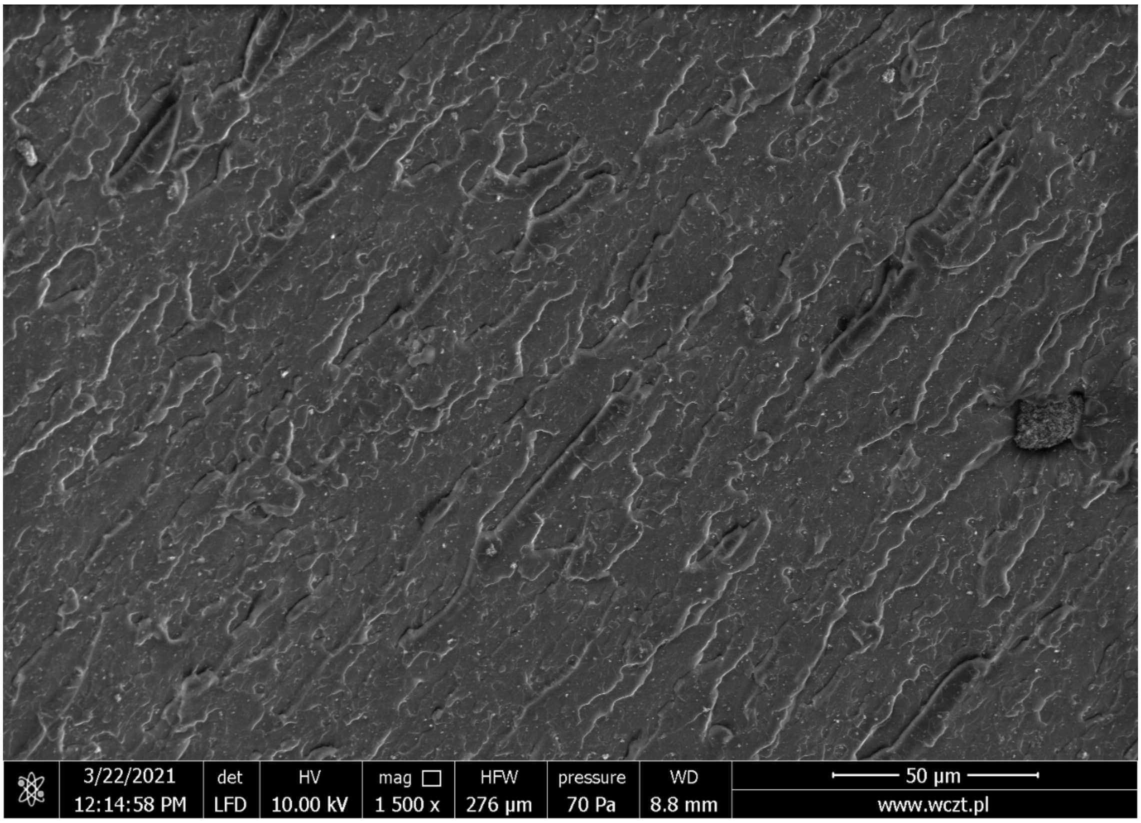

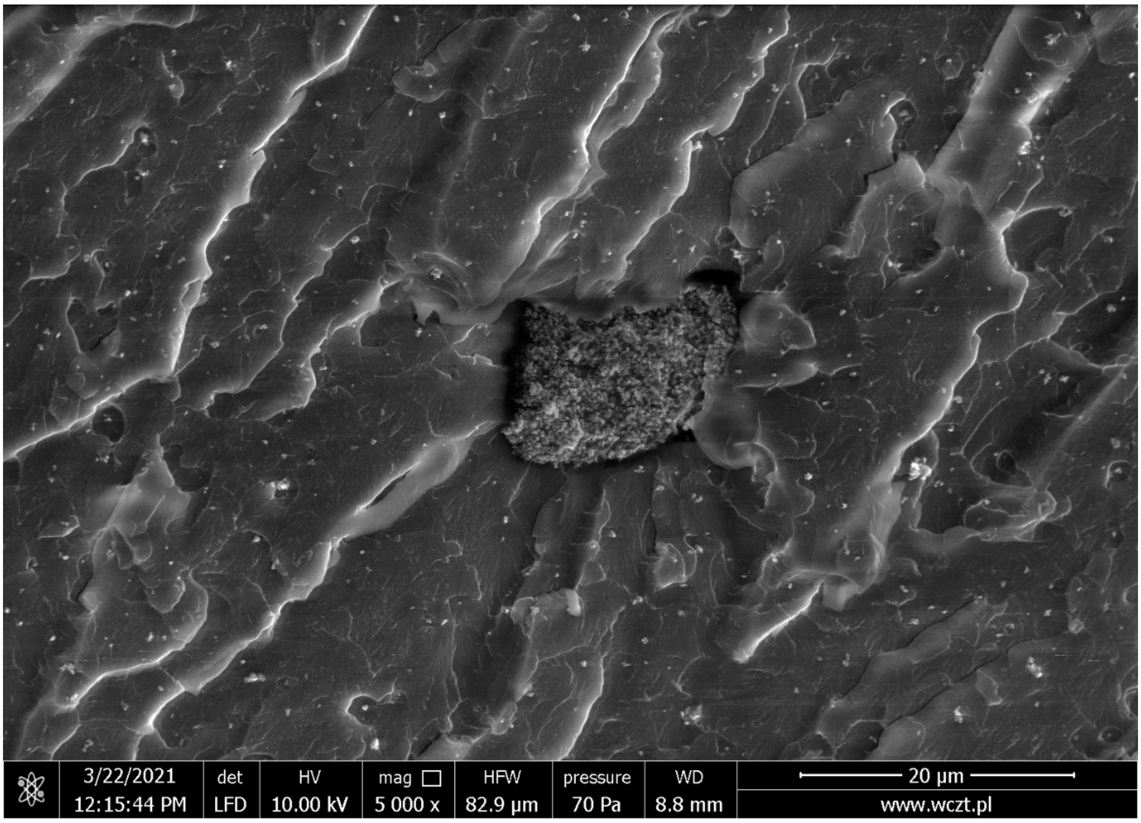

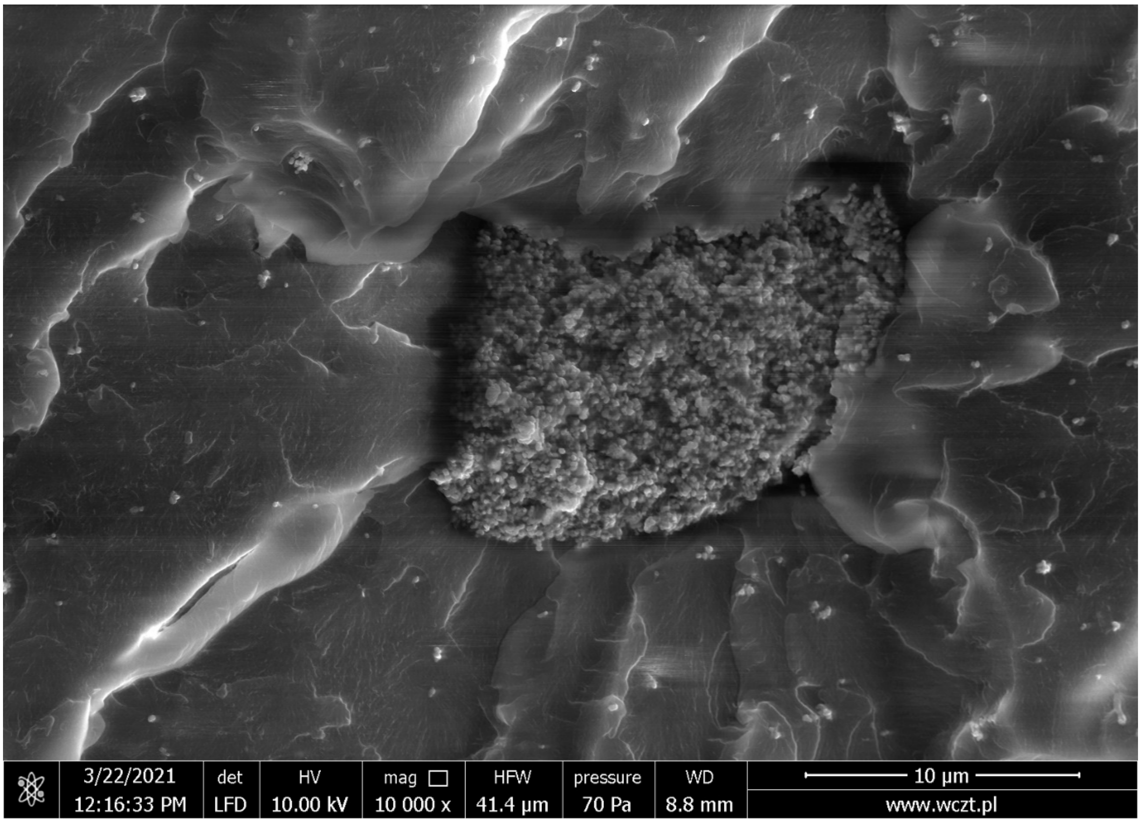
3/22/2021
12:16:33 PM
det
LFD
HV
10.00 kV
mag
10 000 x
HFW
41.4 µm
pressure
70 Pa
WD
8.8 mm
10 µm
www.wczt.pl

1%TiO_2, 1.5% iBu_7SSQ-OEt, mixing pump

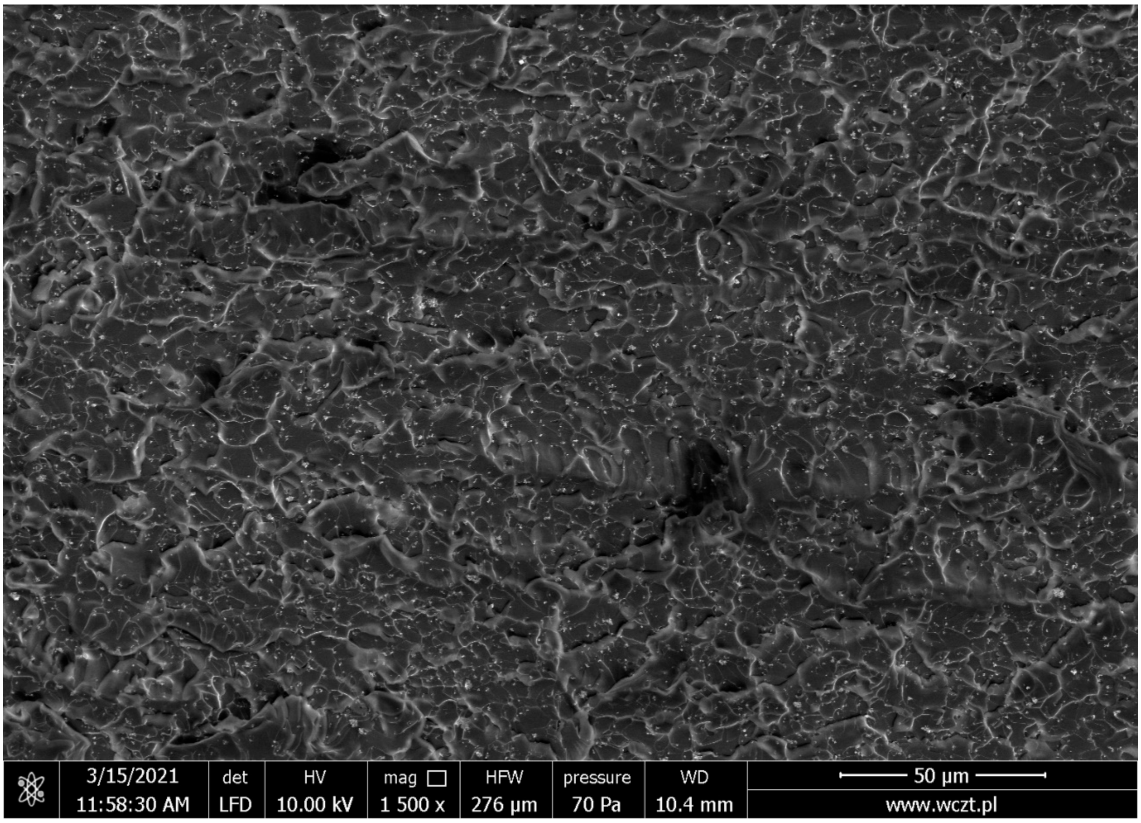

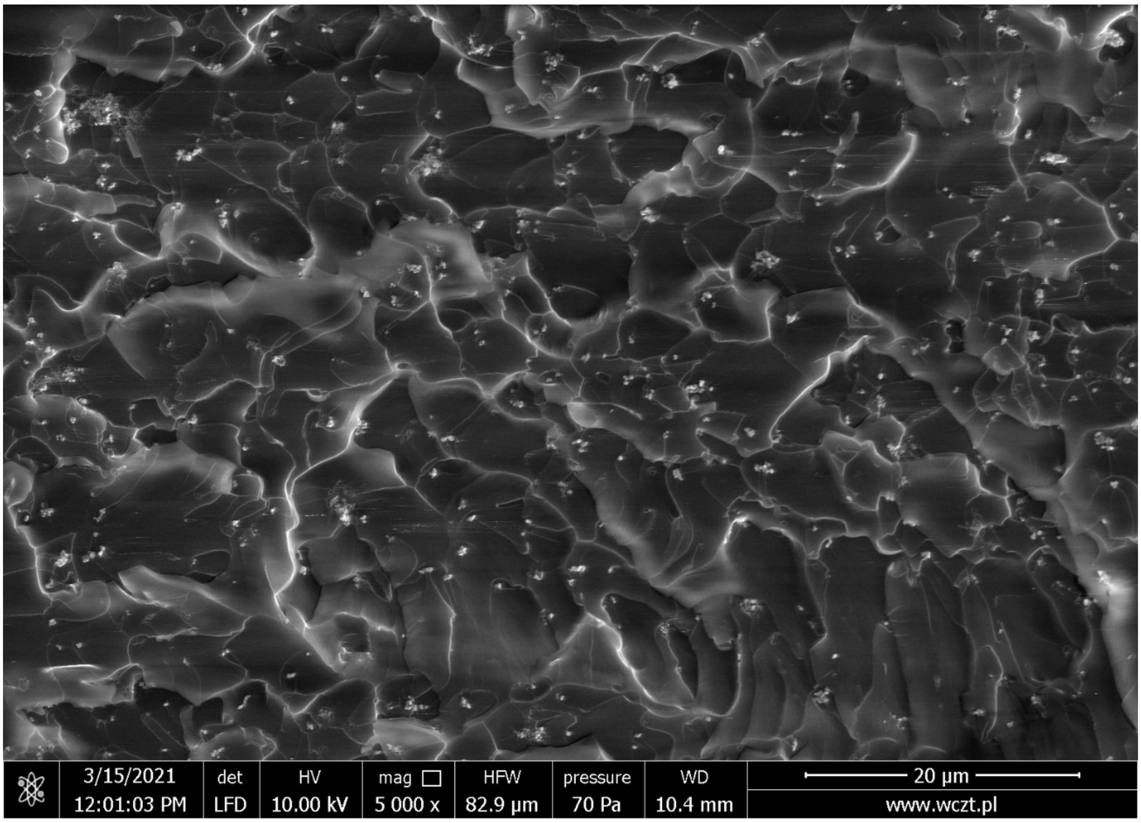

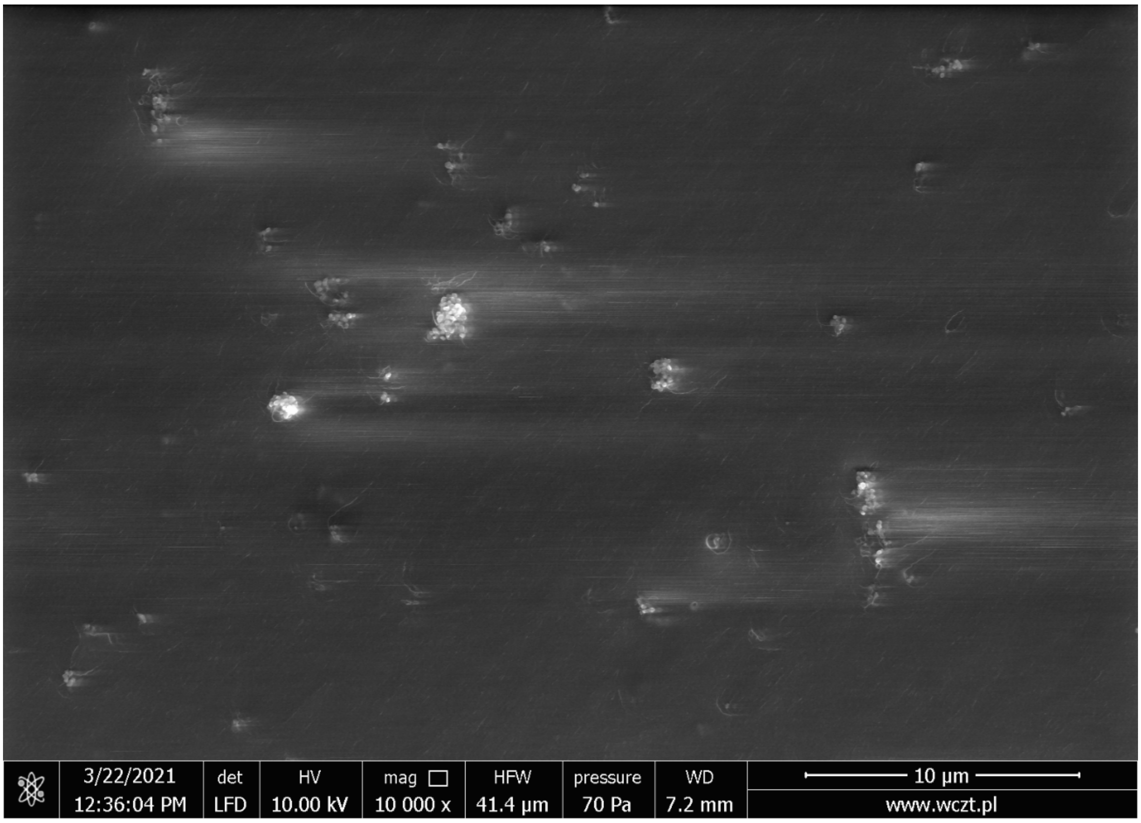
3/22/2021
12:36:04 PM
det
LFD
HV
10.00 kV
mag
10 000 x
HFW
41.4 µm
pressure
70 Pa
WD
7.2 mm
10 µm
www.wczt.pl

2%TiO$_2$, 1.5% iBu$_7$SSQ-OEt, mixing pump

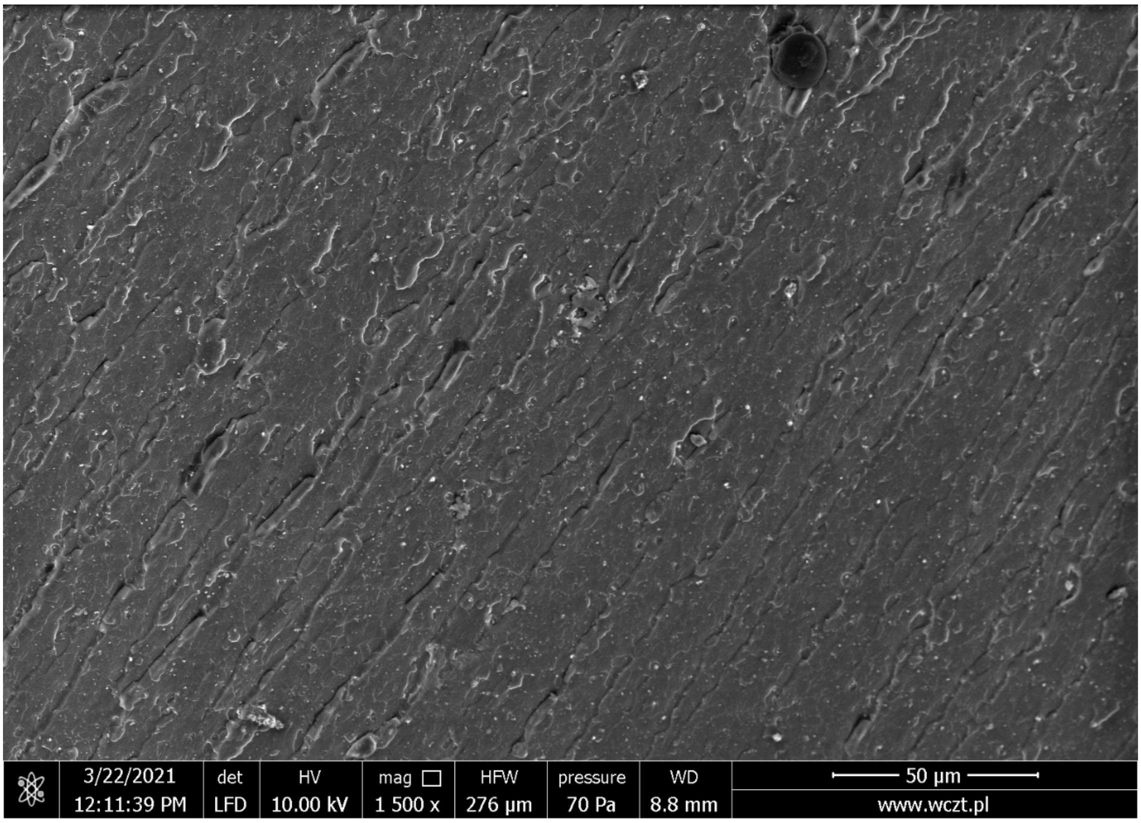

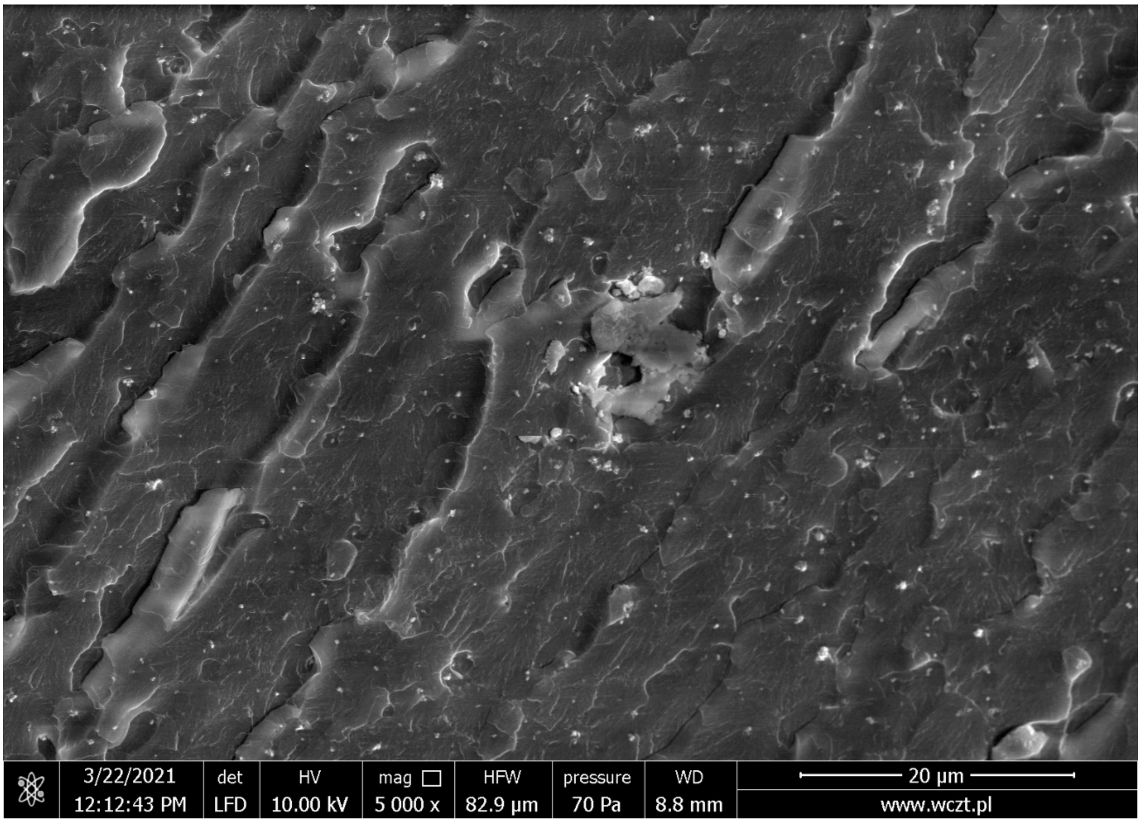

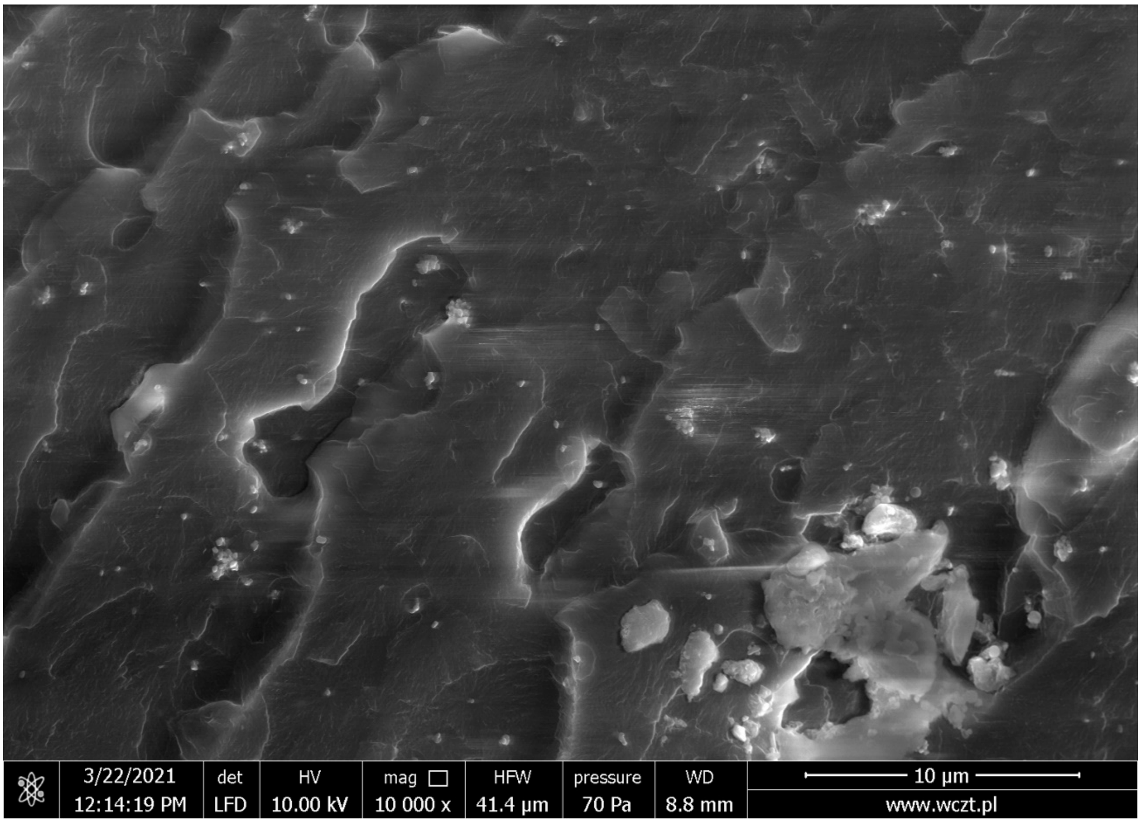
3/22/2021
12:14:19 PM
det
LFD
HV
10.00 kV
mag
10 000 x
HFW
41.4 μm
pressure
70 Pa
WD
8.8 mm
10 μm
www.wczt.pl

1%TiO₂, 0.5% SS-6GP-2TMOS, mixing pump

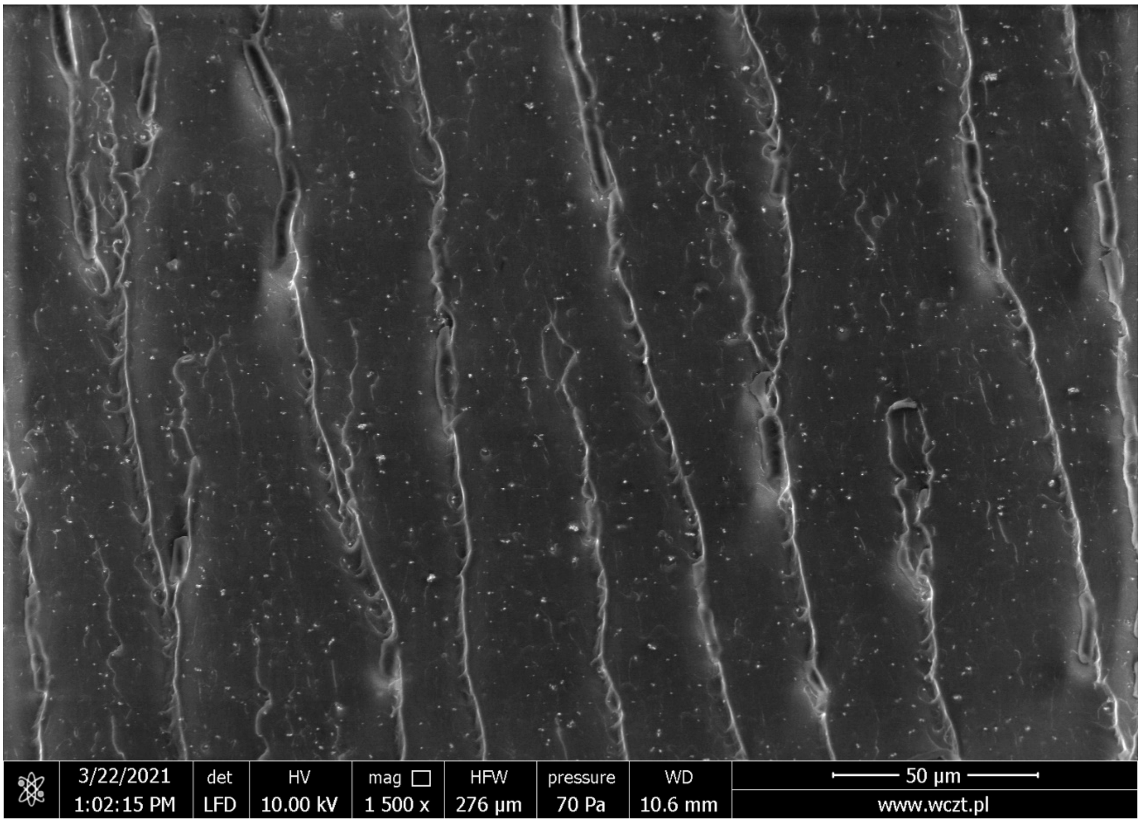

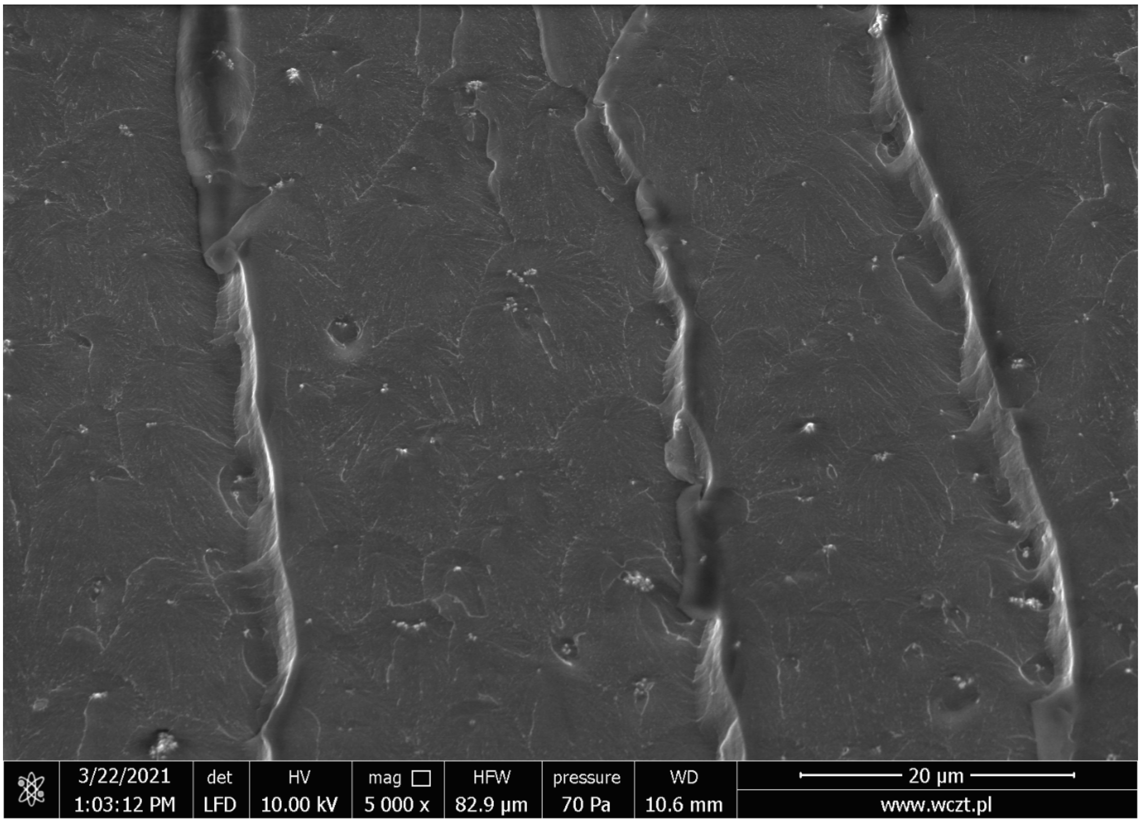

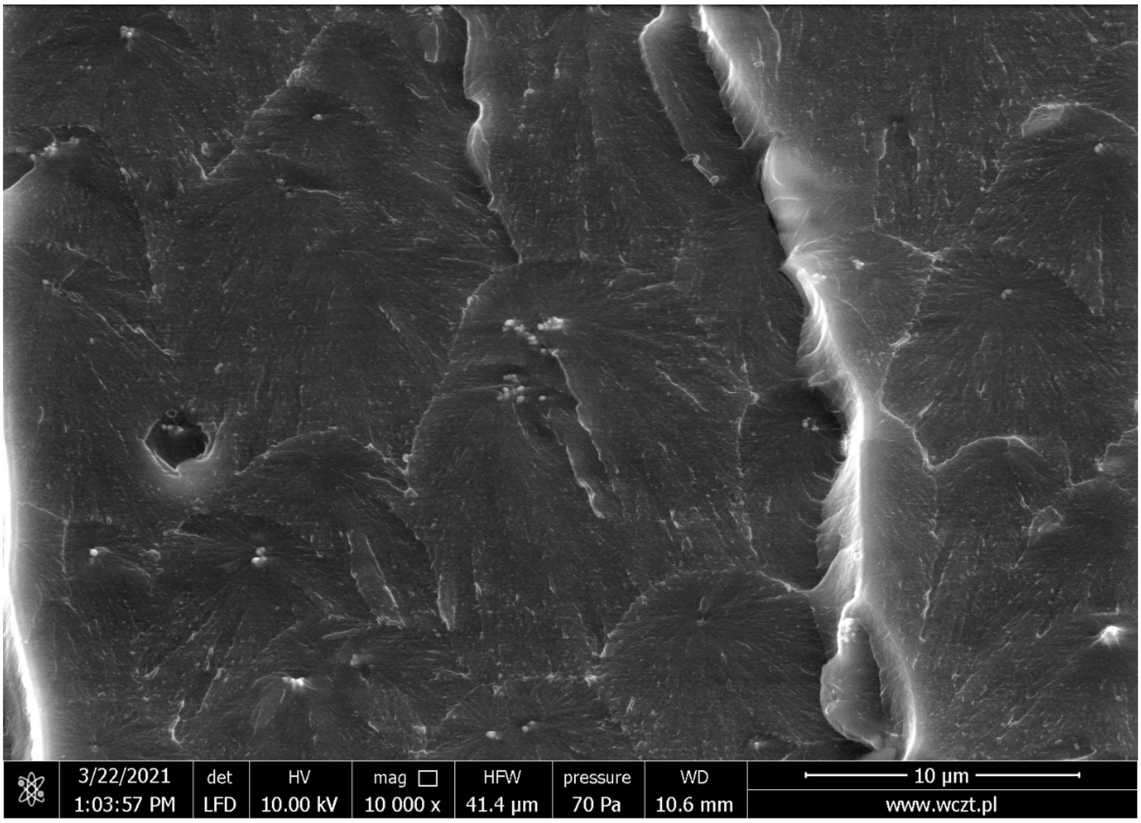
3/22/2021
1:03:57 PM
det
LFD
HV
10.00 kV
mag
10 000 x
HFW
41.4 µm
pressure
70 Pa
WD
10.6 mm
10 µm
www.wczt.pl

2%TiO₂, 0.5% SS-6GP-2TMOS, mixing pump

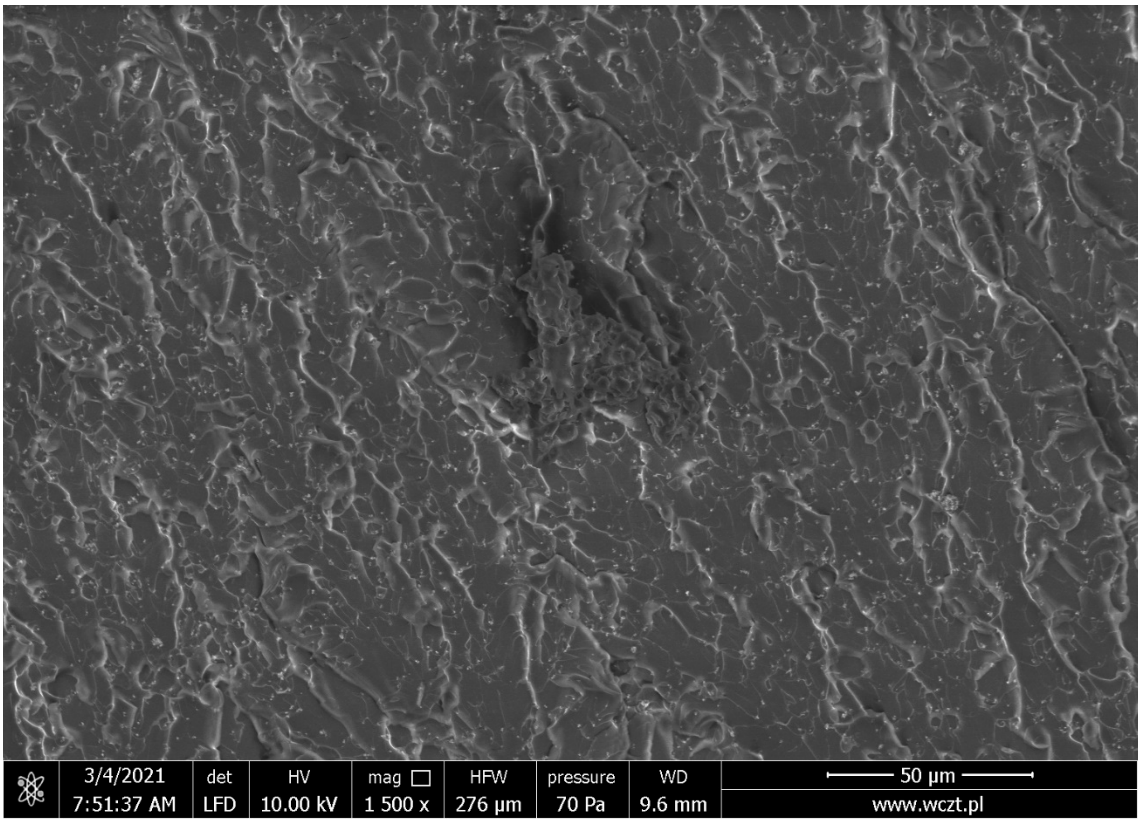

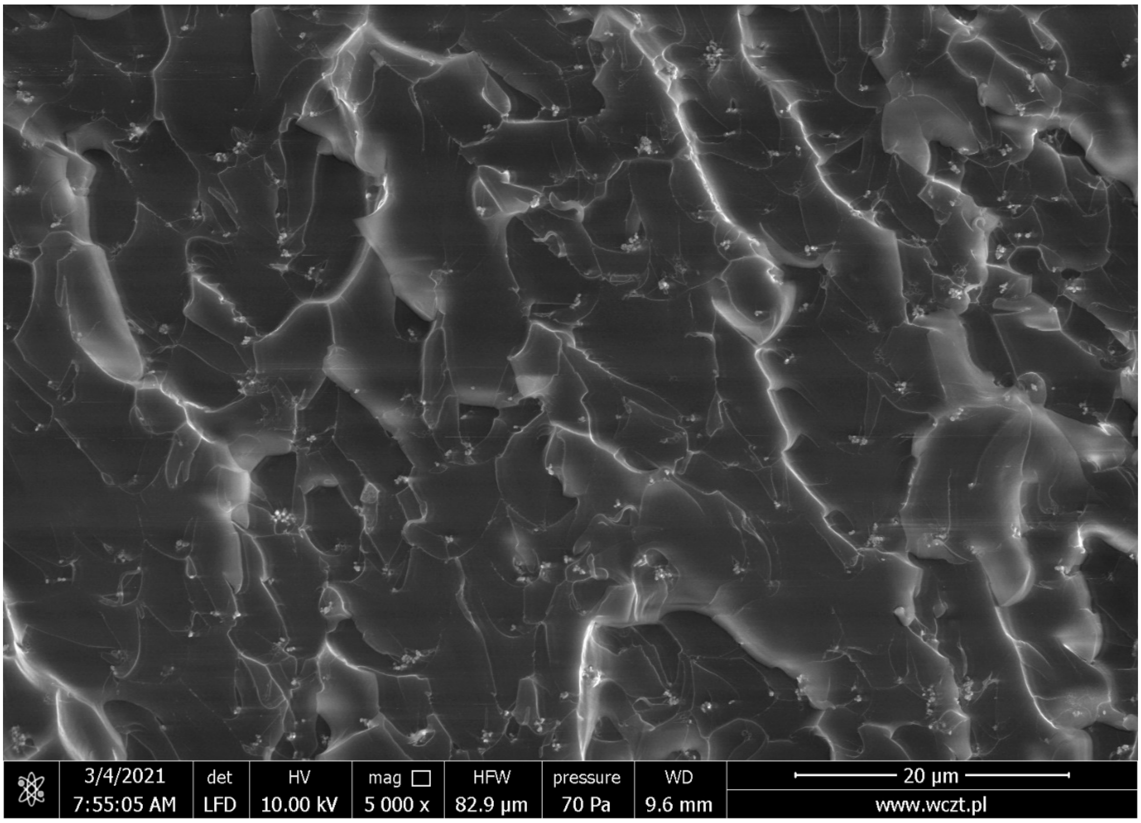

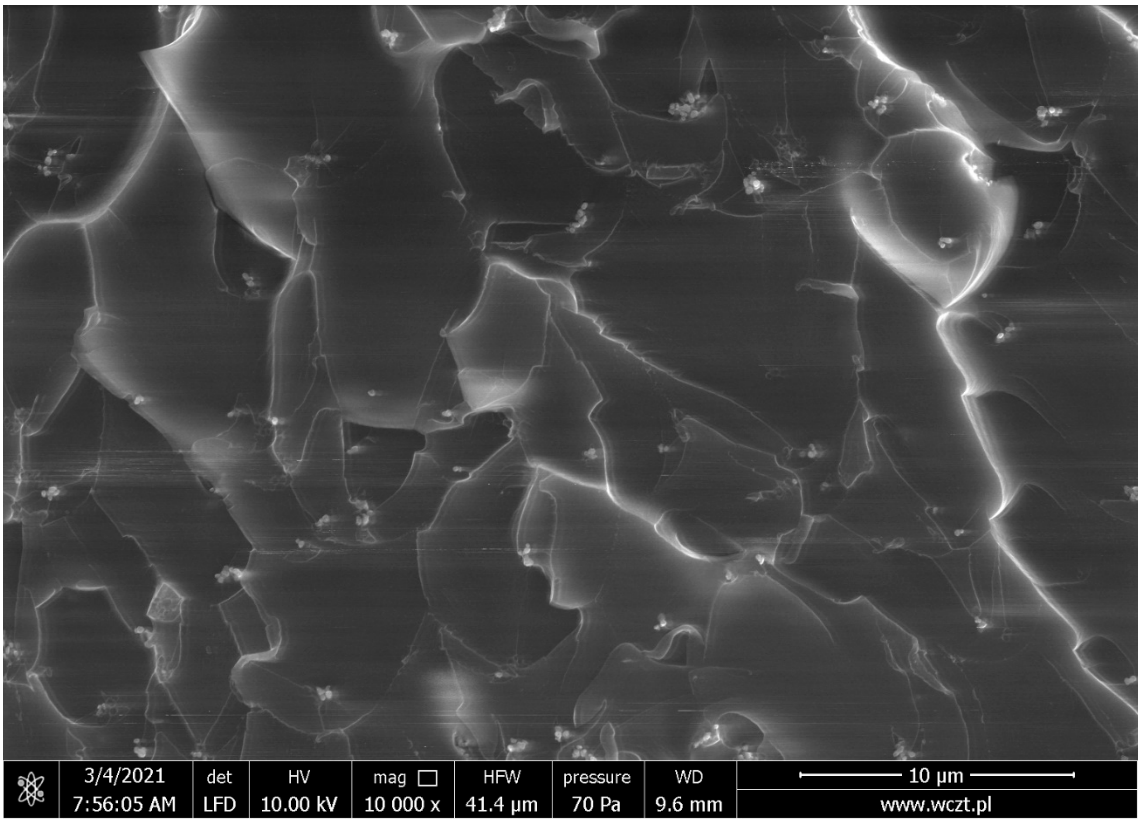
3/4/2021
7:56:05 AM
det
LFD
HV
10.00 kV
mag
10 000 x
HFW
41.4 μm
pressure
70 Pa
WD
9.6 mm
10 μm
www.wczt.pl

1%TiO₂, 1.5% SS-6GP-2TMOS, mechanical stirrer

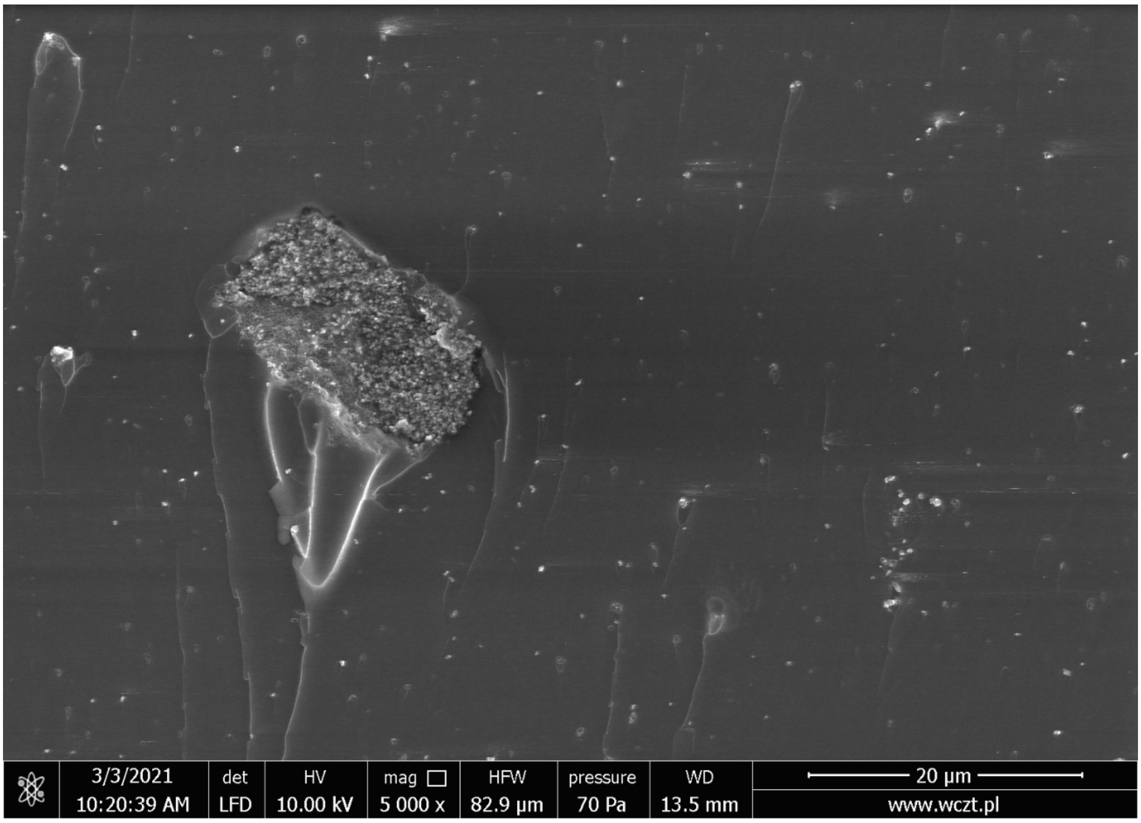

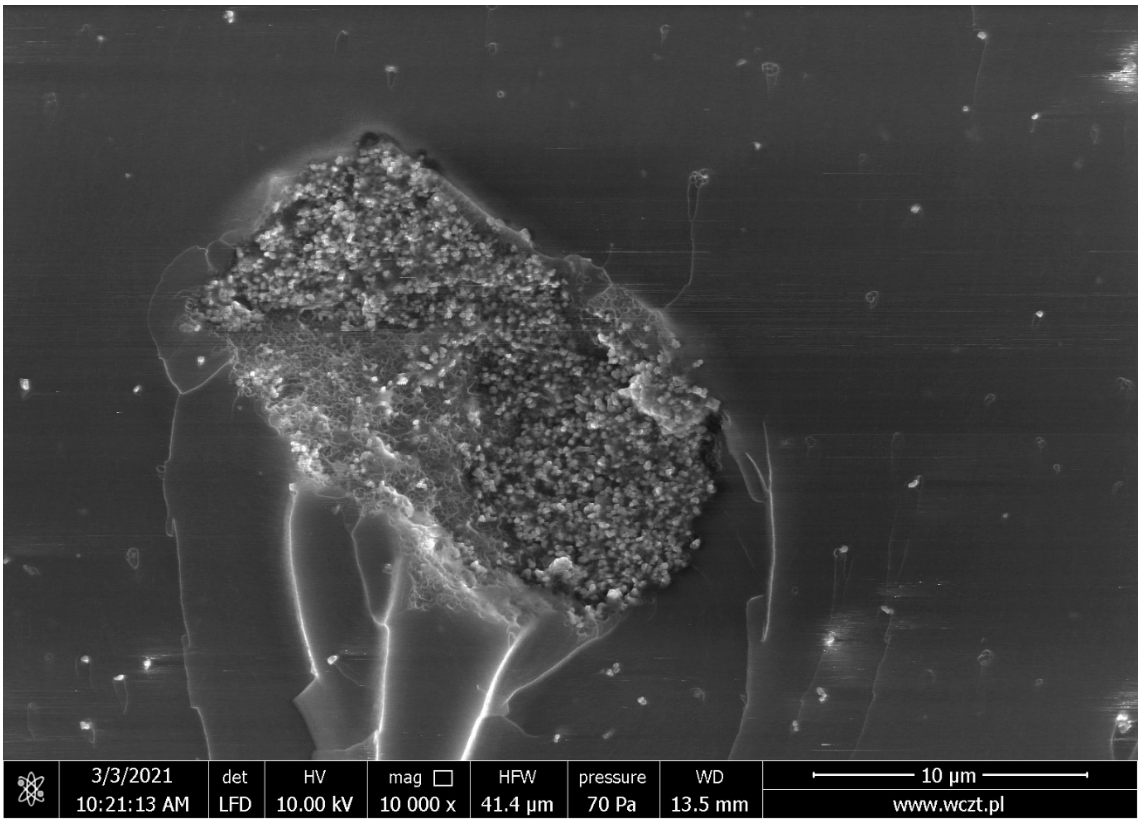

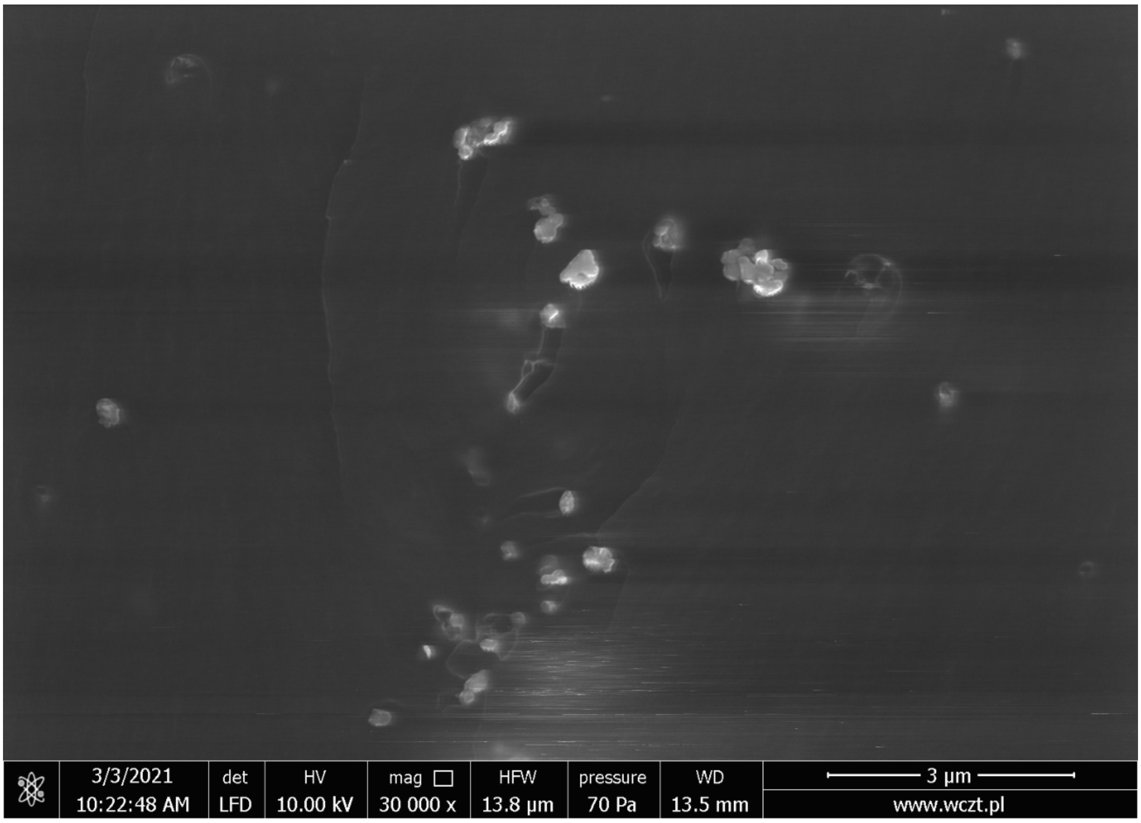
3/3/2021
10:22:48 AM
det
LFD
HV
10.00 kV
mag
30 000 x
HFW
13.8 µm
pressure
70 Pa
WD
13.5 mm
3 µm
www.wczt.pl

1%TiO_2, 1.5% SS-6GP-2TMOS, mixing pump

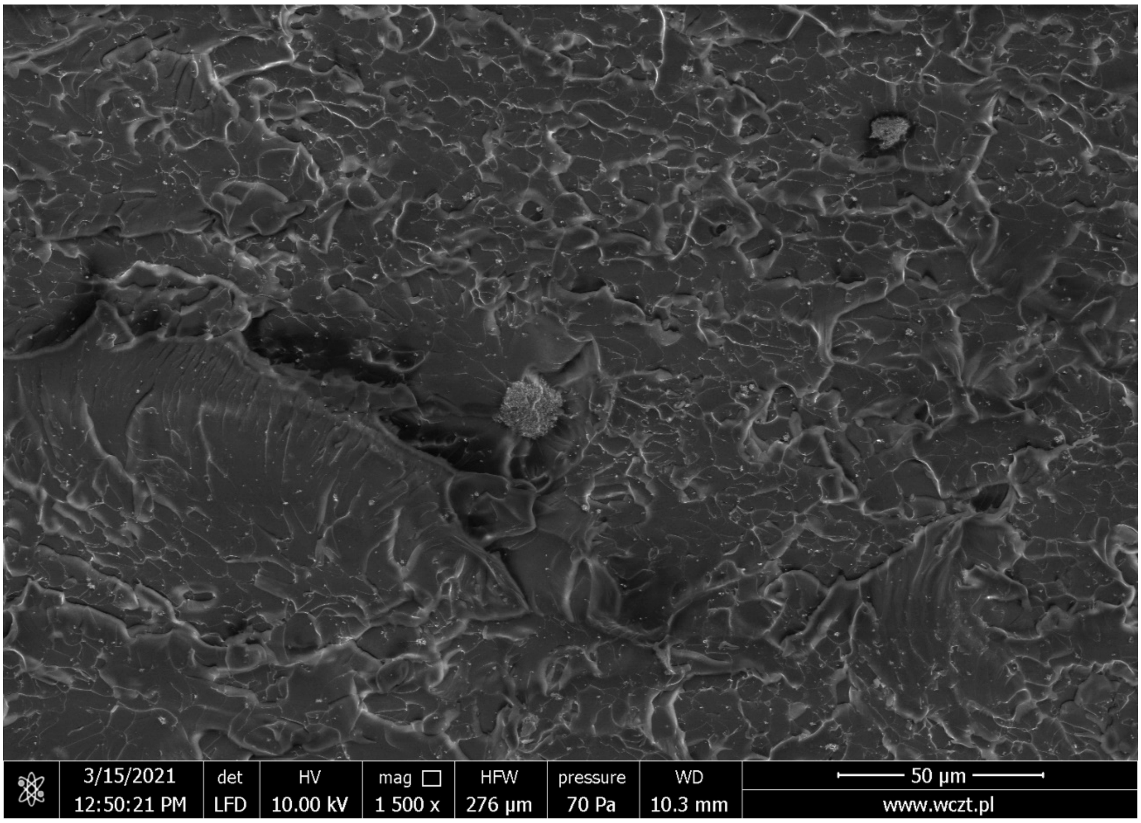

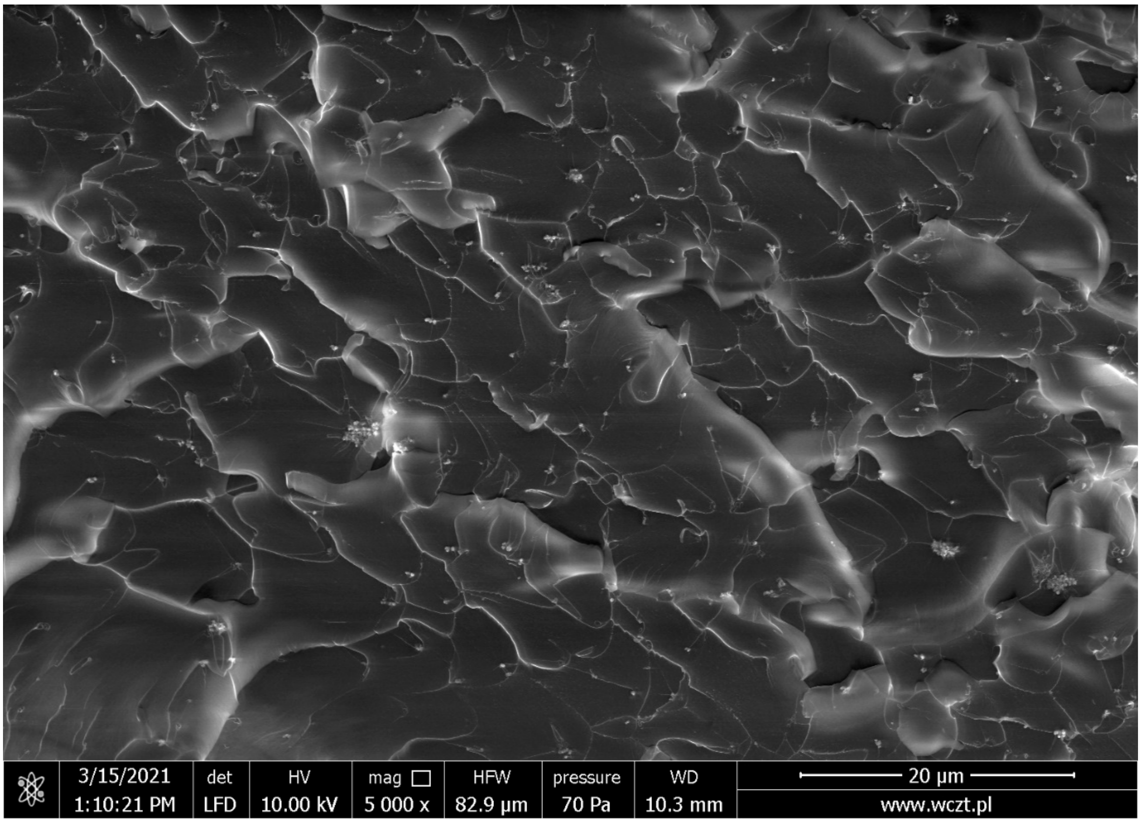

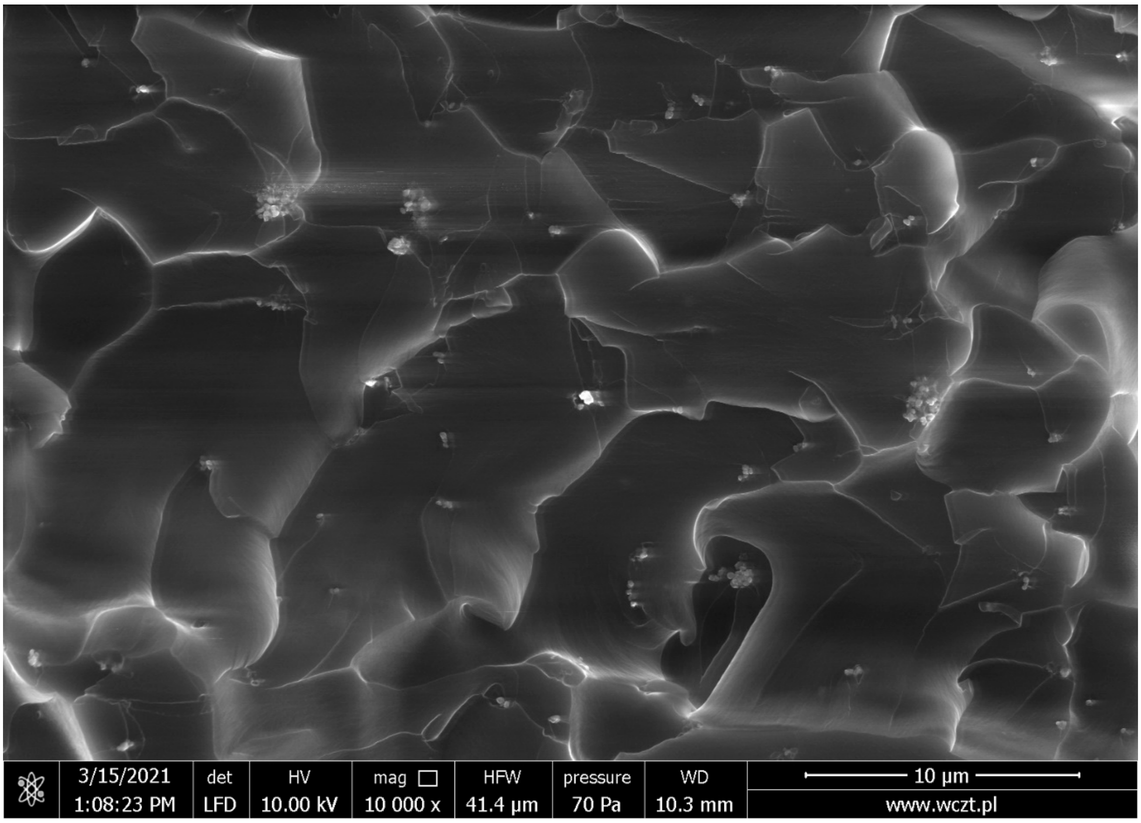
3/15/2021
1:08:23 PM
det
LFD
HV
10.00 kV
mag
10 000 x
HFW
41.4 µm
pressure
70 Pa
WD
10.3 mm
10 µm
www.wczt.pl

2%TiO₂, 1.5% SS-6GP-2TMOS, mixing pump

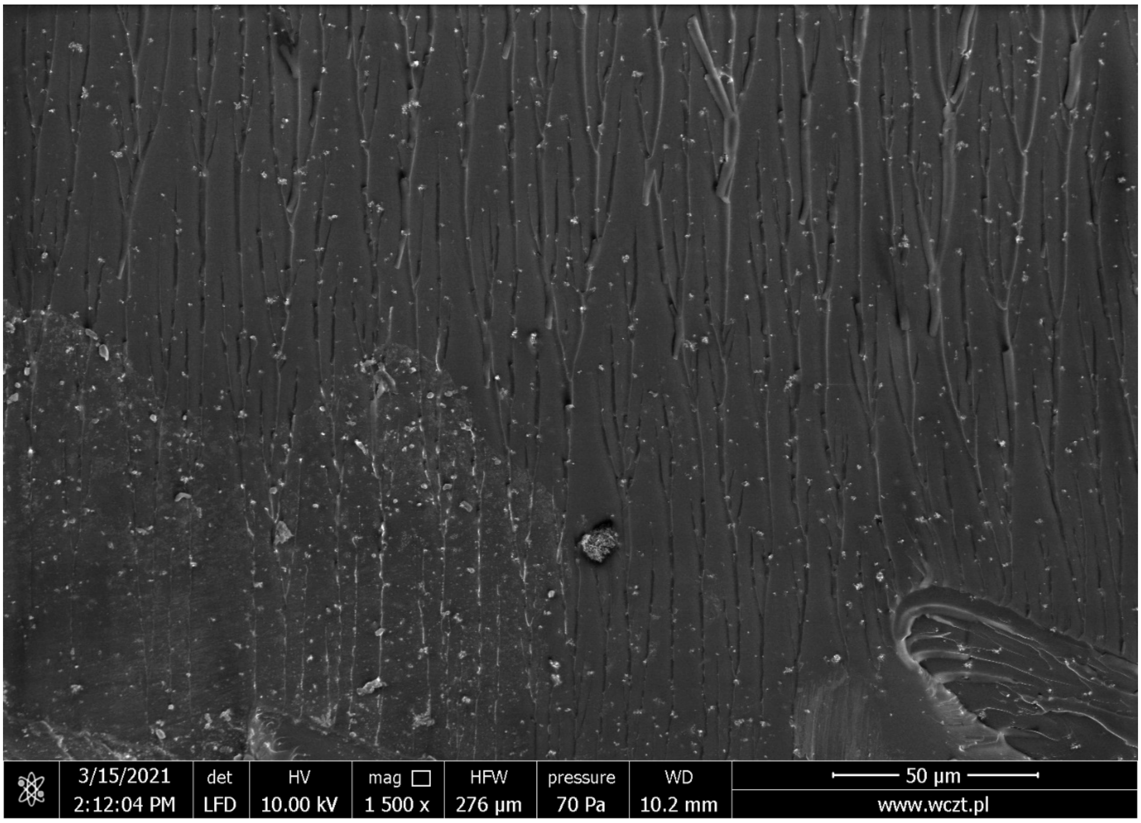

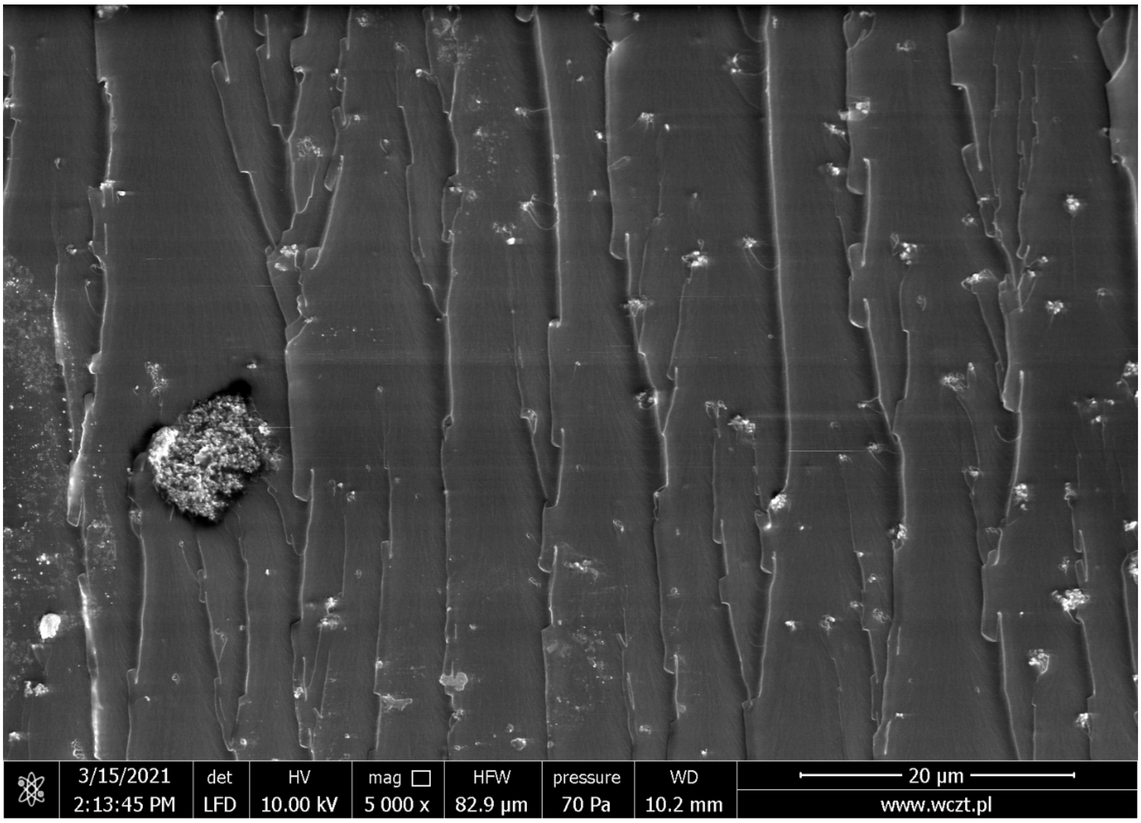

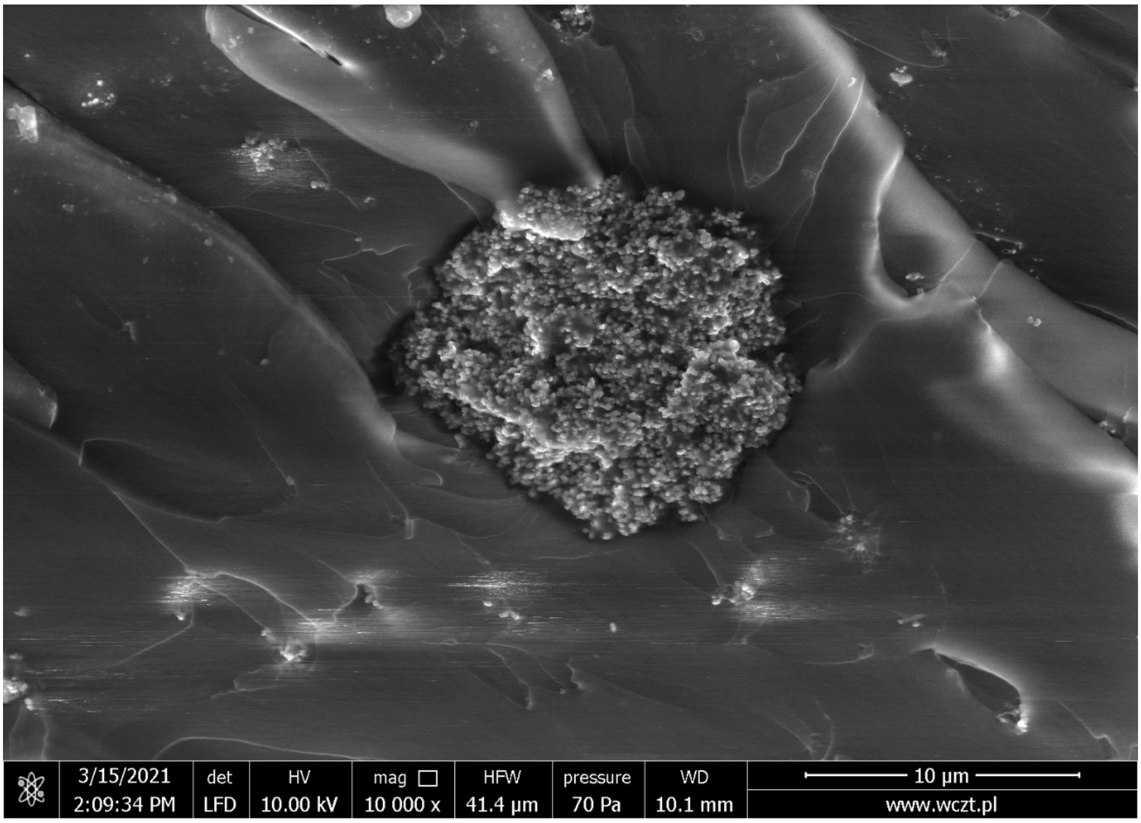
3/15/2021
2:09:34 PM
det
LFD
HV
10.00 kV
mag
10 000 x
HFW
41.4 µm
pressure
70 Pa
WD
10.1 mm
10 µm
www.wczt.pl

1%TiO₂, 0.5% SS-5GP-3TMOS, mixing pump

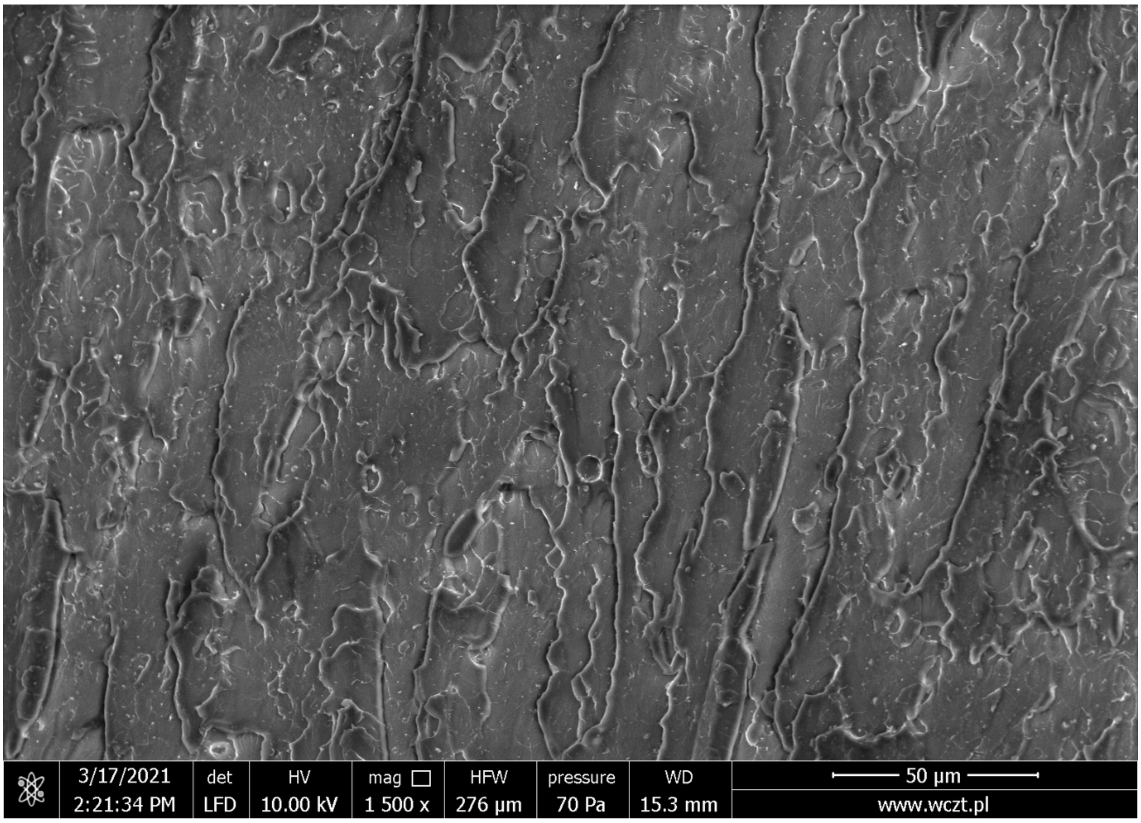

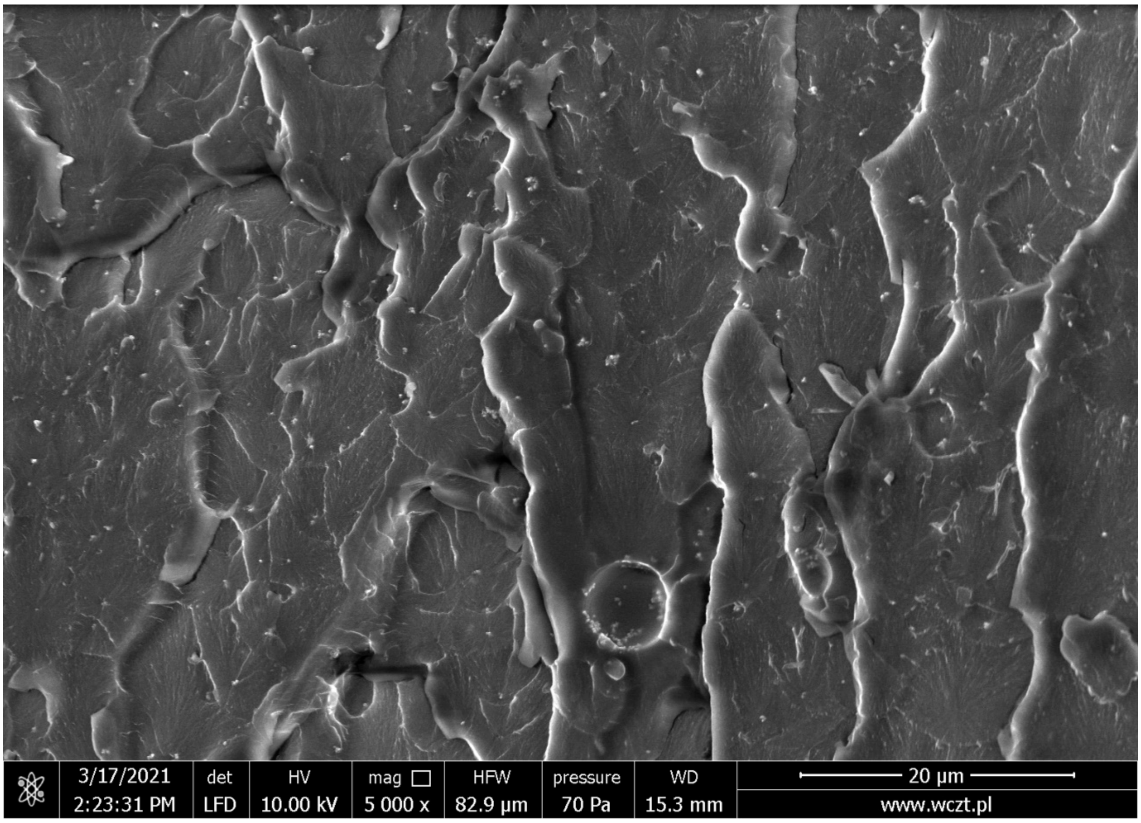

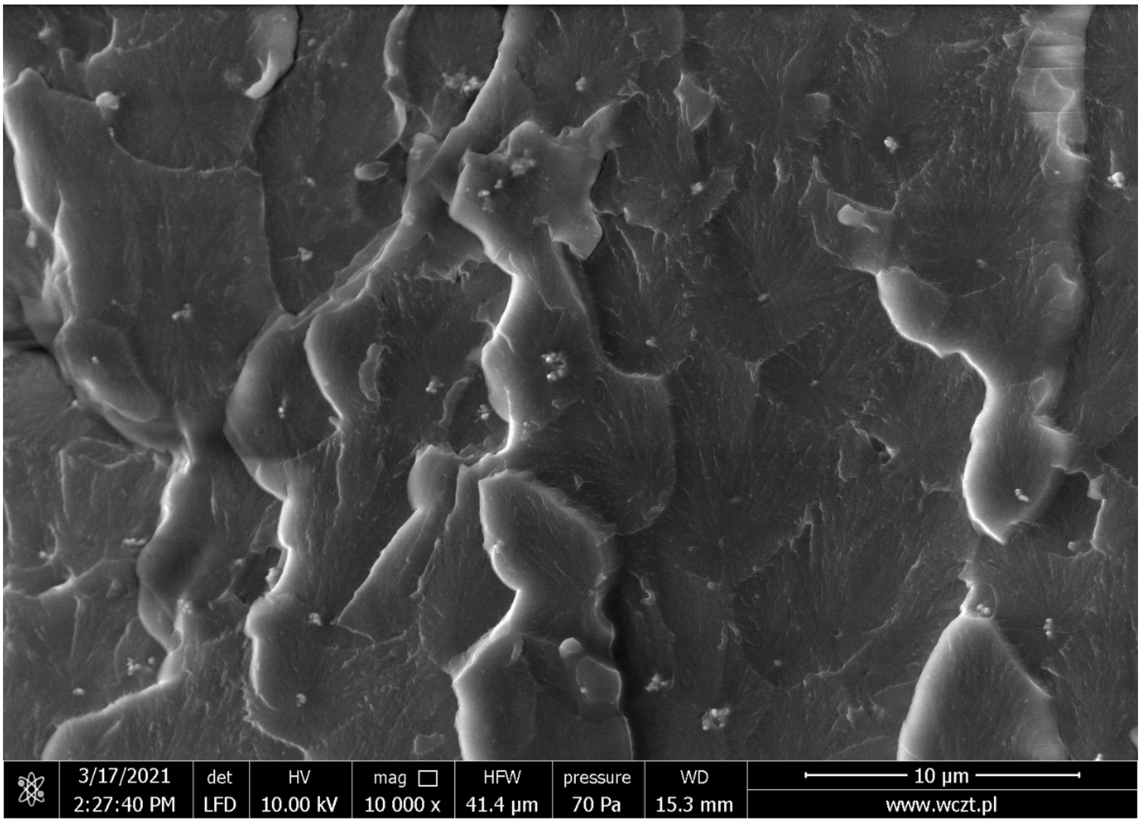
3/17/2021
2:27:40 PM
det
LFD
HV
10.00 kV
mag
10 000 x
HFW
41.4 μm
pressure
70 Pa
WD
15.3 mm
10 μm
www.wczt.pl

2%TiO₂, 0.5% SS-5GP-3TMOS, mixing pump

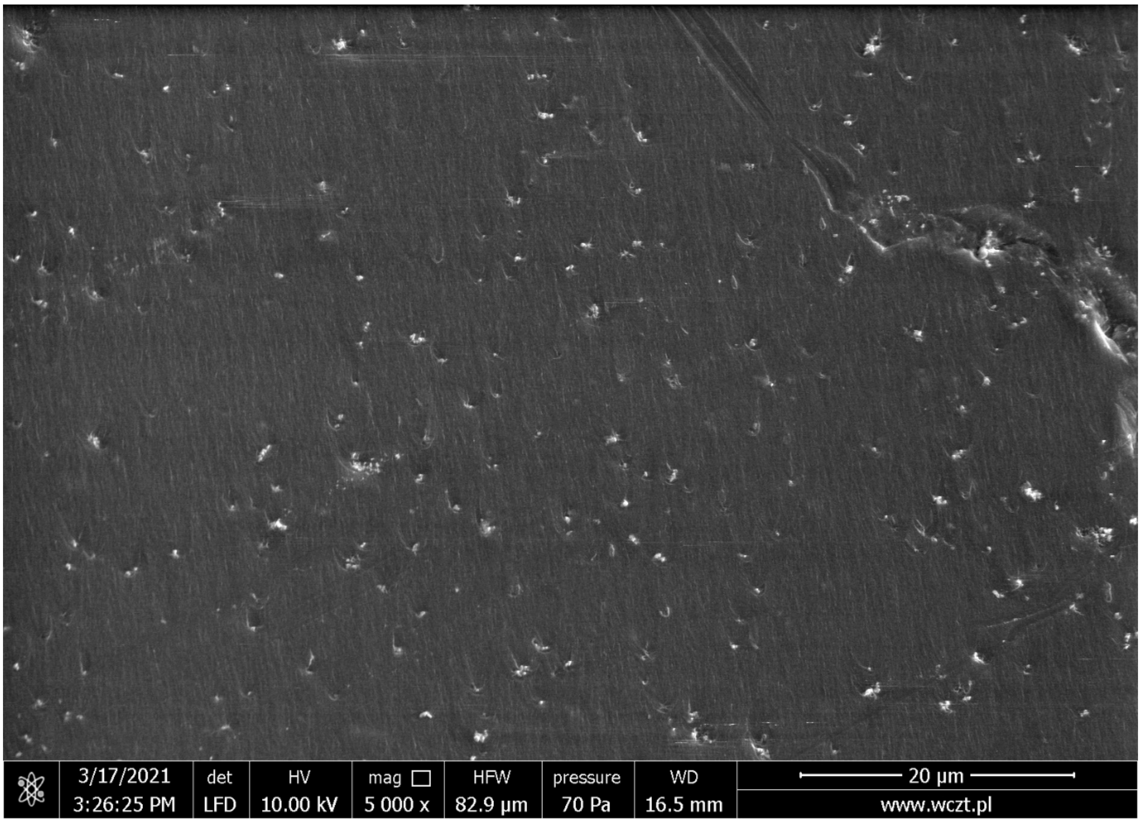

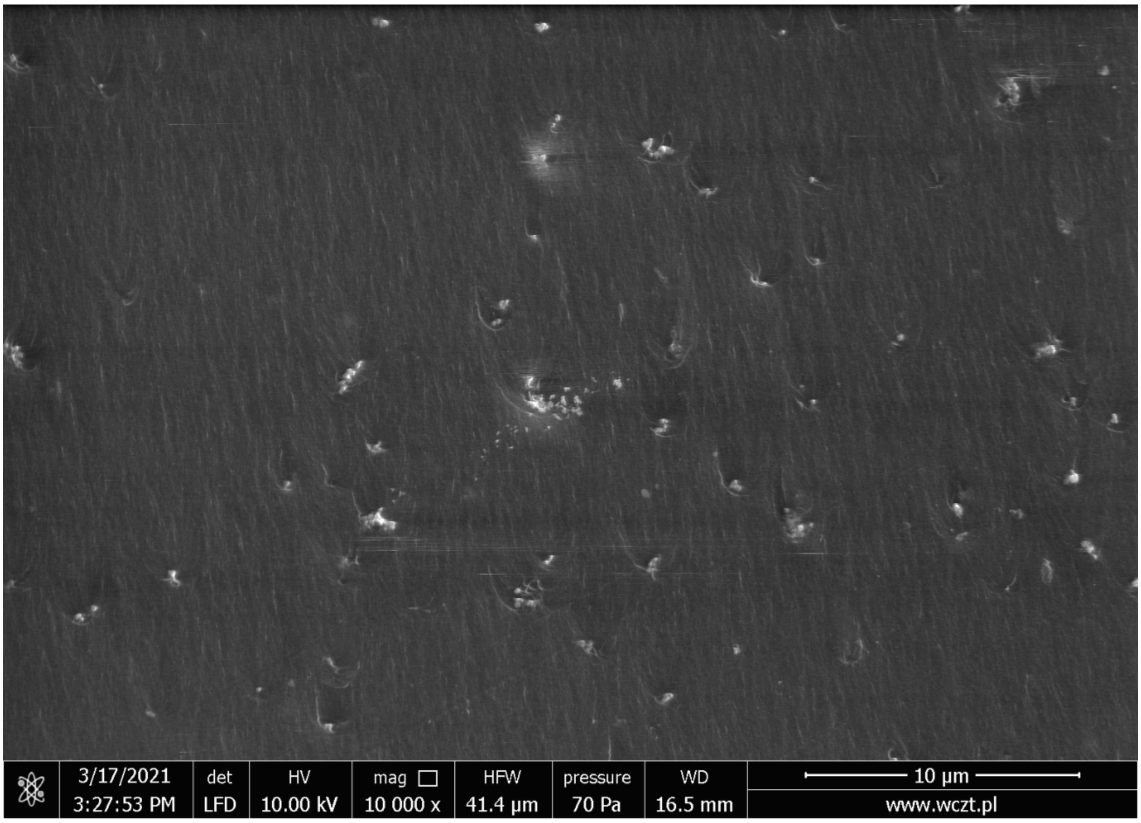

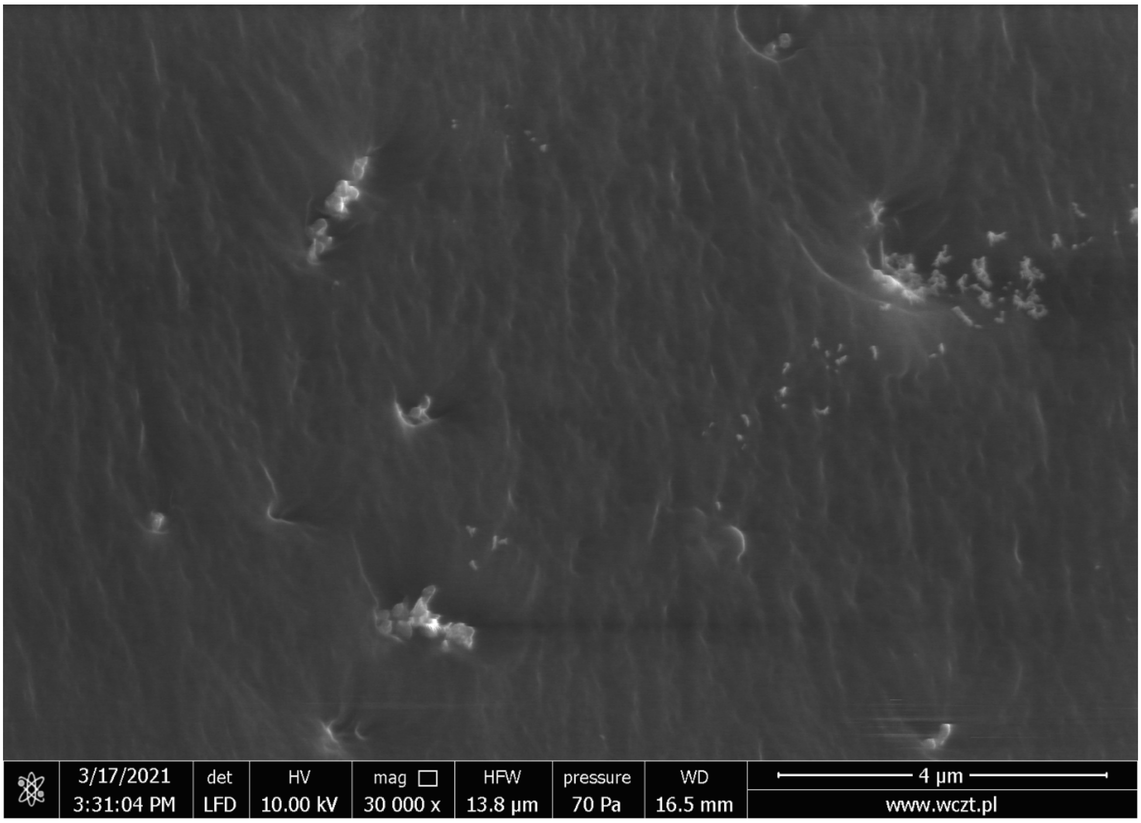
3/17/2021
3:31:04 PM
det
LFD
HV
10.00 kV
mag
30 000 x
HFW
13.8 μm
pressure
70 Pa
WD
16.5 mm
4 μm
www.wczt.pl

1%TiO₂, 1.5% SS-5GP-3TMOS, mixing pump

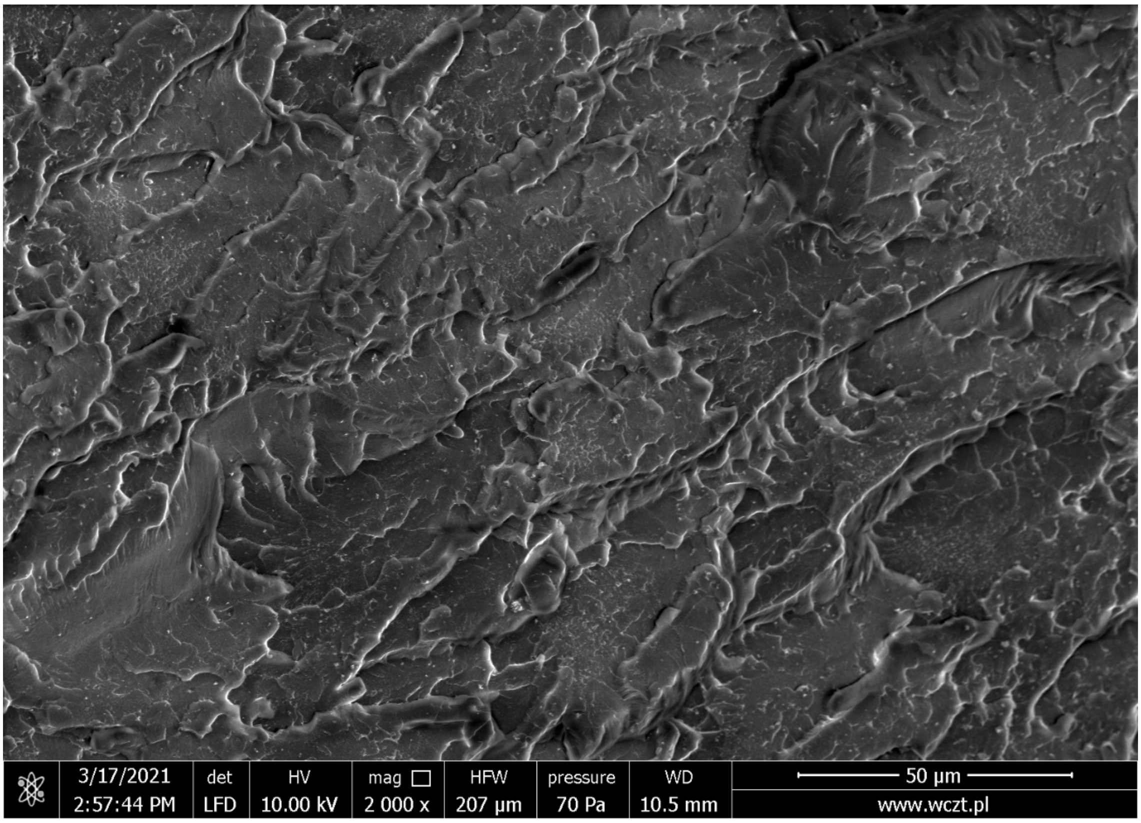

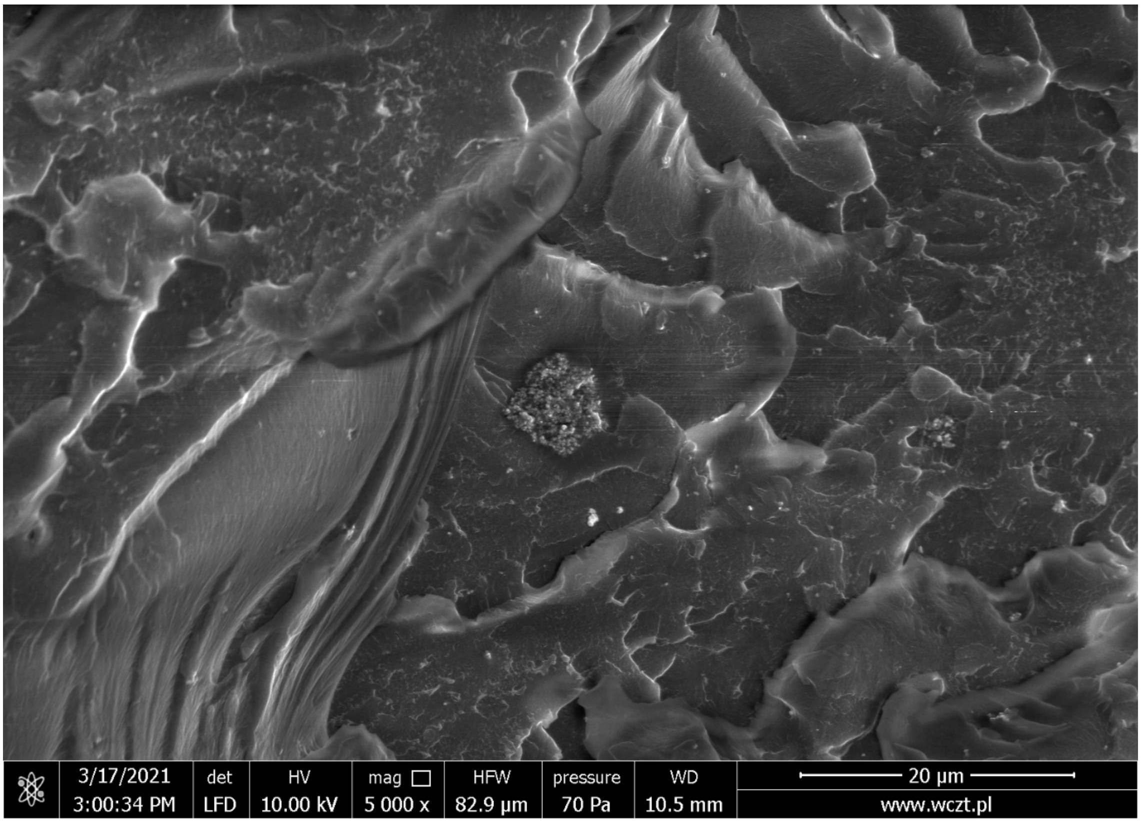

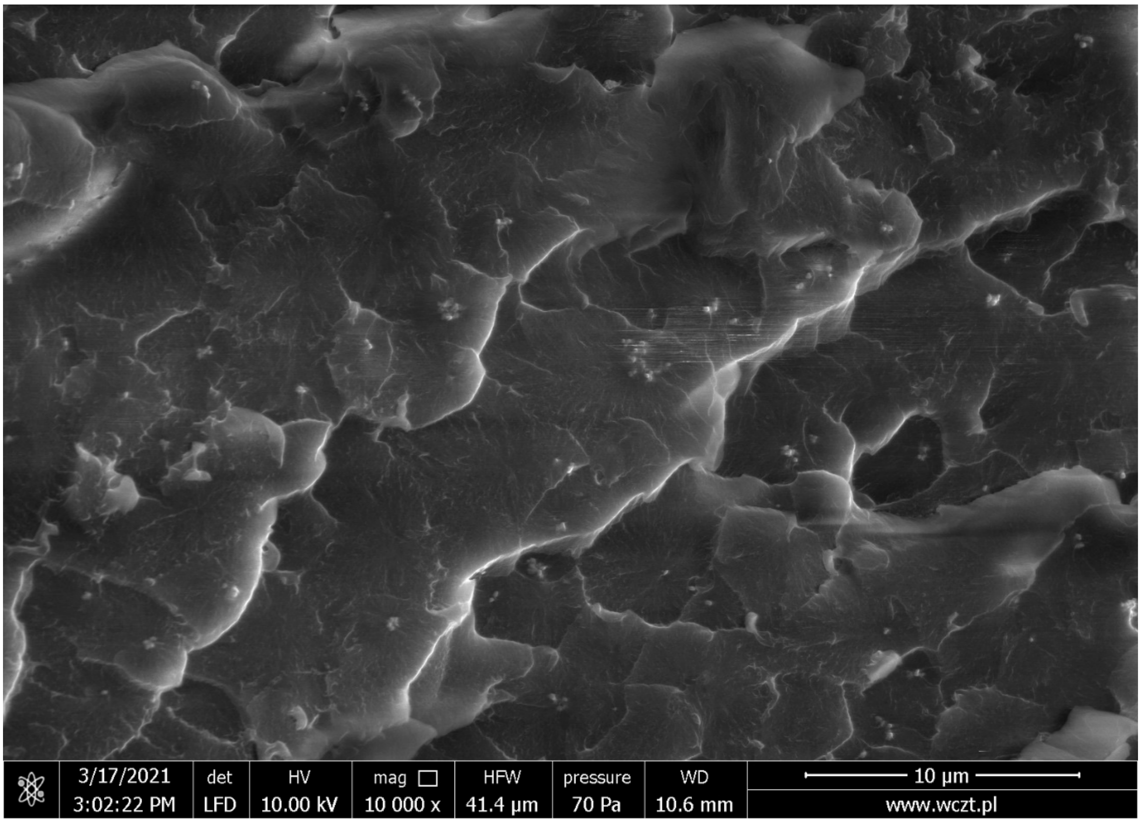
3/17/2021
3:02:22 PM
det
LFD
HV
10.00 kV
mag
10 000 x
HFW
41.4 μm
pressure
70 Pa
WD
10.6 mm
10 μm
www.wczt.pl

2%TiO_2, 1.5% SS-5GP-3TMOS, mixing pump

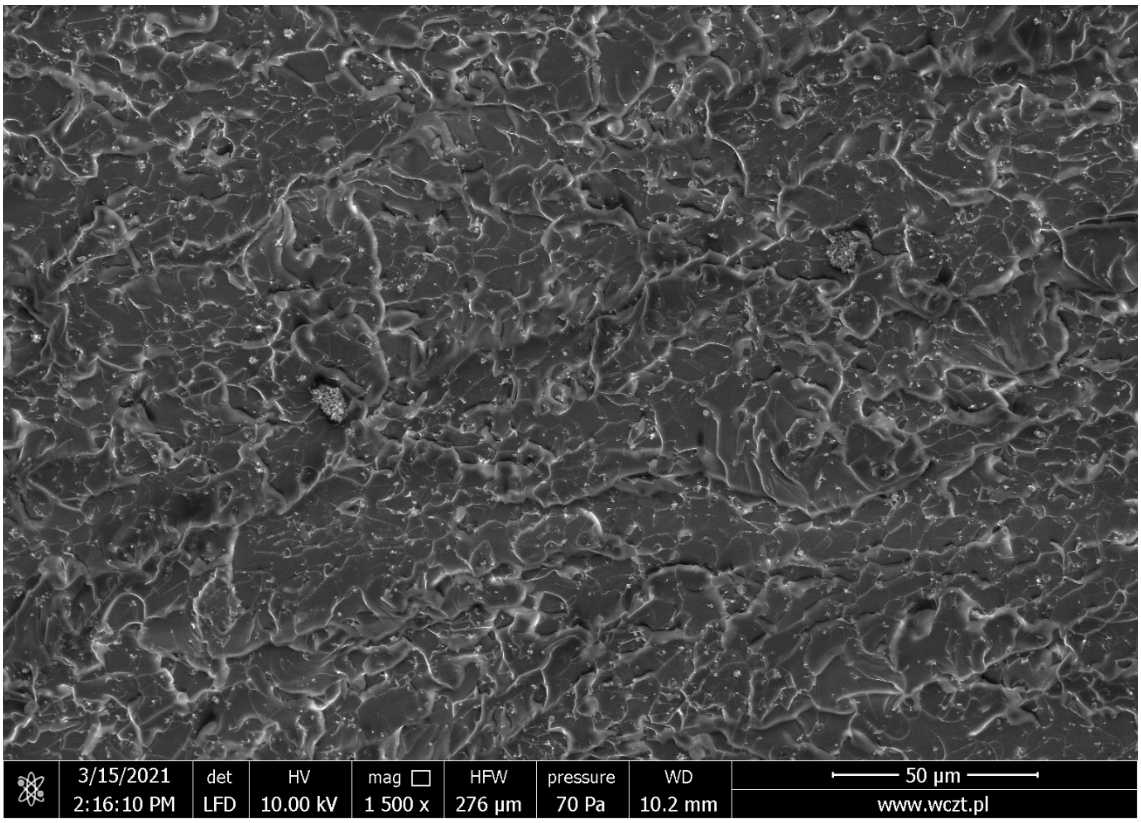

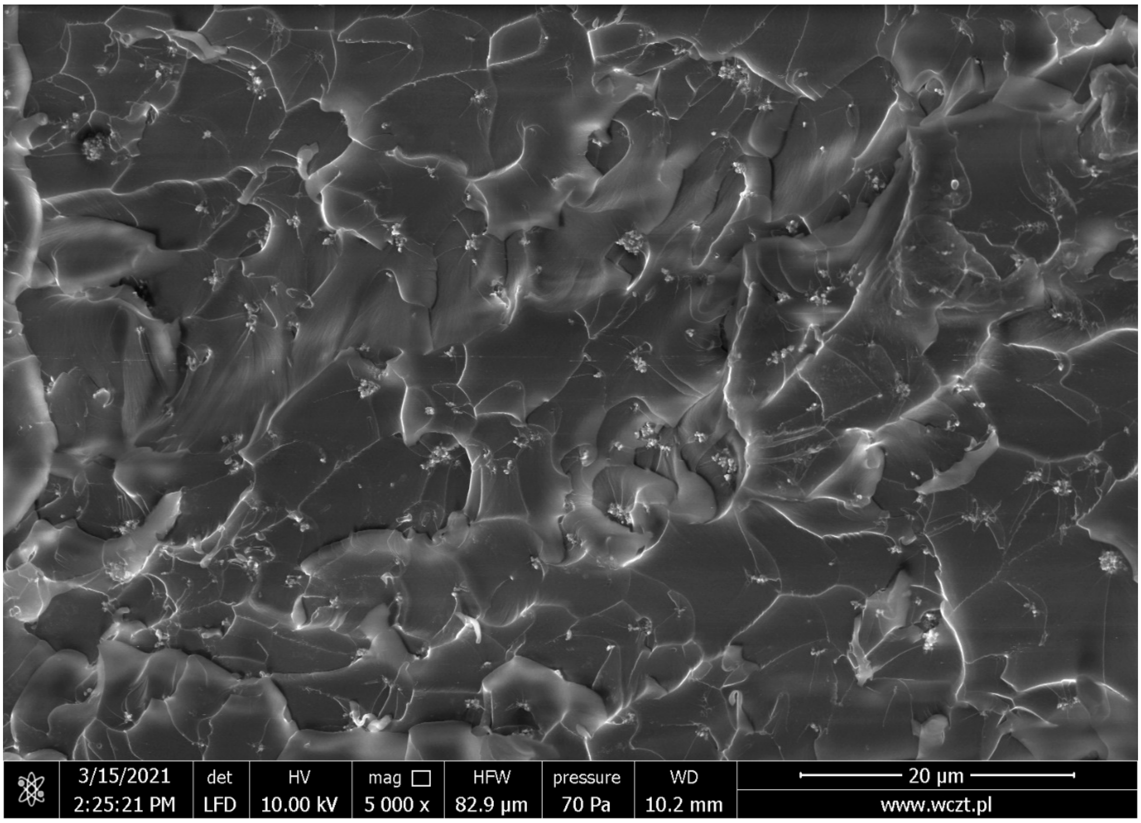

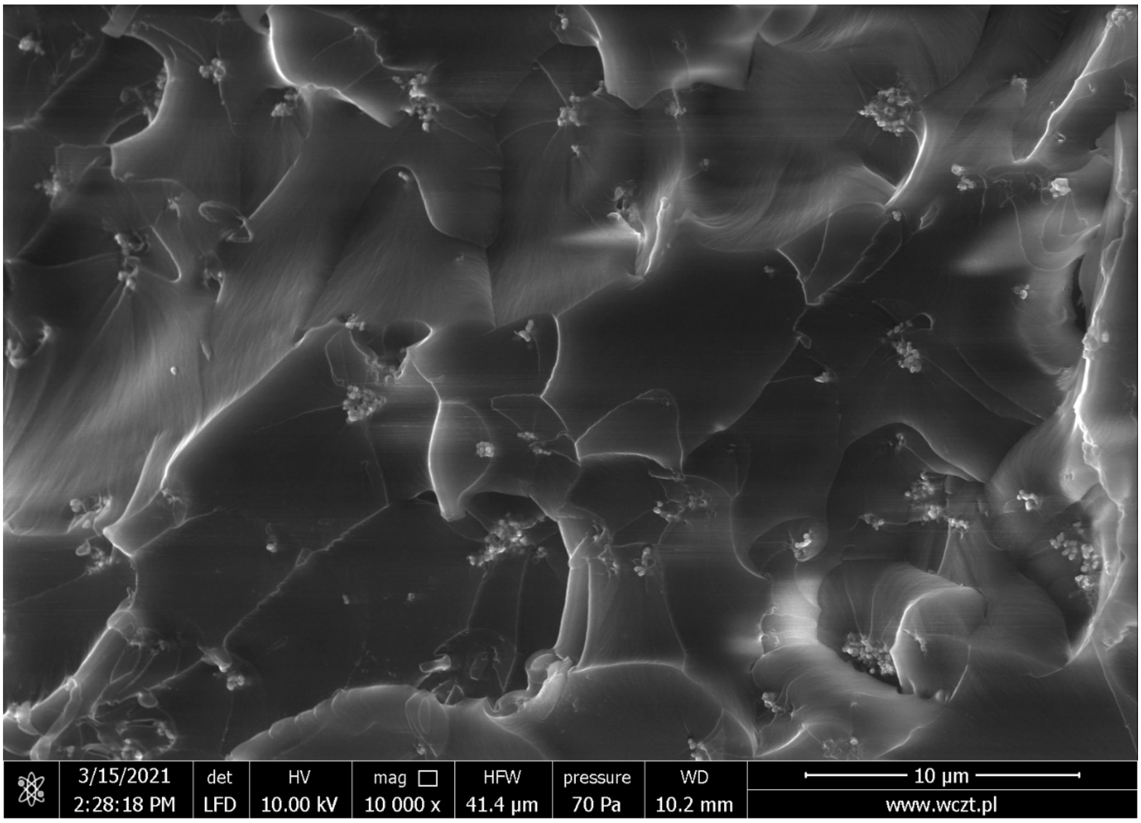
3/15/2021
2:28:18 PM
det
LFD
HV
10.00 kV
mag
10 000 x
HFW
41.4 μm
pressure
70 Pa
WD
10.2 mm
10 μm
www.wczt.pl
